# Exploring the Relationship Between Biofilm Formation and Antibiotic Resistance Genes in Clinically Isolated *Klebsiella pneumoniae*

**DOI:** 10.1155/ijm/3833882

**Published:** 2025-10-16

**Authors:** Hevar N. Abdulqadir, Kochar I. Mahmood

**Affiliations:** Medical Laboratory Science Department, Charmo University, Sulaymaniyah, Iraq

**Keywords:** antibiotic resistance, ARG, biofilm formation, biofilm gene, *K. pneumoniae*, *mrkA*, RT-qPCR

## Abstract

Persistent pathogens pose a significant global health burden, contributing to increased morbidity and mortality rates worldwide. This study investigates the relationship between clinically relevant biofilm-associated and antibiotic resistance genes in *Klebsiella pneumoniae* isolates. Biofilm-forming capabilities of the isolates were evaluated, and their biomass was quantitatively analyzed. The presence of biofilm-associated and resistance genes (*mrkA*, *blaSHV*, *blaTEM*, and *blaKPC*) in the samples was identified using conventional PCR. Gene expression levels were quantified via RT-qPCR under acidic and neutral pH conditions, and the results were analyzed statistically to evaluate significance. All clinical isolates were found to be biofilm formers. PCR analysis revealed that a significant proportion of the isolates harbored the *mrkA*, *blaSHV*, and *blaTEM* genes, with prevalence rates of 78%, 89%, and 63%, respectively. In contrast, the *blaKPC* gene was absent. Statistical analysis revealed a significant (*p* = 0.0357) association between the presence of the *mrkA* gene and elevated expression of the *blaSHV* gene. Strains harboring the *mrkA* gene demonstrated higher resistance gene expression compared to *mrkA*-negative strains, particularly under neutral conditions (pH 7). In conclusion, these findings suggest that biofilm may contribute to antibiotic resistance not just by acting as a physical layer but also by modulating the expression of resistance genes. This observed relationship highlights the importance of designing novel therapies that can target both biofilm and resistance mechanisms to combat persistent infections.

## 1. Background

Despite the transformative impact of penicillin and other antibiotics, bacterial resistance began to emerge as early as the 1950s [1]. By the early 2000s, bacterial infections had resurged as a critical public health threat worldwide [2]. Among the most concerning developments is the rise of pan-drug-resistant (PDR) and extensively drug-resistant (XDR) bacterial strains. These strains resist nearly all antibiotics, rendering treatment exceptionally difficult [3]. *Klebsiella pneumoniae*, a strain strongly associated with hospital-acquired infection, exemplifies this threat [4]. Its biofilm formation shields it from both antibiotics and the host immune system, contributing to high morbidity and mortality rates [5]. It is linked to pneumonia, bloodstream infections, surgical site infections, and meningitis [6]. Antibiotic misuse has exacerbated the emergence of resistant strains in *K. pneumoniae*, particularly in healthcare settings [7]. Consequently, patient mortality has risen, highlighting the urgency of the global crisis.

Biofilms are microbial communities enclosed in a self-produced polymeric matrix [8]. These organized communities attach themselves to surfaces (e.g., hospital equipment and benches), enhancing bacterial survival against external stressors such as antibiotics [9]. Biofilm formation progresses through adhesion, colonization, and maturation: Each stage is crucial in resisting external stress [10]. During this process, bacterial strains communicate using quorum sensing, triggered by shifting microenvironments, such as lower oxygen levels and pH shifts [11]. The biofilm matrix reduces antibiotic penetration, which frequently leads to treatment failure [12]. Studies show that bacterial strains within biofilms are 10 to 1000 times more resistant to antibiotics than planktonic cells [13]. Furthermore, biofilms actively promote the exchange of antibiotic resistance genes across bacterial strains, enhancing community resilience [14]. Despite understanding biofilms, conflicting evidence exists linking biofilm genes to antibiotic resistance genes, with some indicating that they regulate each other and others suggesting that there are no direct links between them [15, 16]. Understanding this gap is critical in developing therapies targeting biofilm-associated infections and reducing mortality rates from biofilm-associated infections.

Prior research on the relationship between biofilm formation genes and antibiotic resistance genes has suggested that resistant strains often form biofilms [15]. However, most studies have relied on statistical correlations rather than direct experimental evidence, limiting the empirical validation of this relationship [17, 18]. According to a recent review, bacterial strains possessing antibiotic resistance genes are often associated with enhanced biofilm formation (e.g., increased biomass and structural density) compared to their antibiotic-sensitive counterparts [19]. Despite these correlations, some studies report findings that suggest no molecular mechanisms linking biofilm and antibiotic resistance genes [16]. These inconsistent findings highlight the importance of understanding the molecular mechanisms linking antibiotic resistance and biofilm genes, enabling adapted therapies for biofilm-associated infections and significantly reducing the global burden of bacterial infections.

Bacterial infections have increasingly become a global threat to public health, causing treatment failure, poor outcomes for patients, and elevated mortality rates [20]. A significant factor contributing to the treatment failure of bacterial infections is the ability of many bacterial strains to form biofilms, which confer enhanced resistance to antimicrobial therapies. The formation of biofilms enables bacteria to persist in hostile environments, complicating treatment regimens and exacerbating the burden on healthcare systems [17]. To address this challenge, this study will investigate the interaction between antibiotic resistance genes and biofilm genes to elucidate their regulatory interplay in biofilm communities and their contribution to treatment failure. With current knowledge of the subject, this research may reveal new insights and contribute to a better understanding of gene regulation. This understanding could enable targeted therapies that may improve patient outcomes and reduce the worldwide burden of bacterial infections.

This research is aimed at investigating the link between antibiotic resistance genes and biofilm genes in clinically isolated *K. pneumoniae*. The *K. pneumoniae* strain is frequently associated with hospital-acquired infections, and its increasing resistance to antibiotics is a cause of concern for public health [21]. To investigate the relationship between biofilm formation and antimicrobial resistance (AMR) in *K. pneumoniae*, the study focused on four genes (*mrkA*, *blaSHV*, *blaTEM*, and *blaKPC*). The *mrkA* gene encodes the major subunit of Type 3 fimbriae, which is critical for biofilm development by promoting adhesion [22]. Previous research has shown that the *mrkABCDF* operon is essential for biofilm formation, with deletion of any of the genes resulting in reduced ability to produce biofilm [23, 24]. Also, the prevalence of the *mrkA* gene among the *K. pneumoniae* strains in Iraq is considered high, making it ideal for investigation [25]. Furthermore, three beta-lactamase genes (*blaSHV*, *blaTEM*, and *blaKPC*) were selected for their high prevalence and clinical impact. These genes encode enzymes that hydrolyze beta-lactam antibiotics, contributing to treatment failure. Previous studies have shown that these genes are widely distributed among biofilm-forming strains, making them ideal candidates for testing [26, 27]. Therefore, this study aims to determine whether biofilm formation influences antibiotic resistance using phenotypic and genotypic methods.

## 2. Methods

### 2.1. Sample Collection and Purification of Clinical Isolates

Between August and October 2024, clinical isolates of *K. pneumoniae* were systematically collected from multiple hospitals in Sulaymaniyah. The isolates were transported under sterile conditions and immediately cultured on MacConkey agar to ensure purity. Following cultivation, the samples were incubated at 37°C for 24 h. After incubation, pure cultures were subcultured in nutrient broth and incubated overnight under the same conditions. Subsequently, 30% glycerol was added to the cultures, and the tubes were stored at −80°C for long-term preservation.

### 2.2. Molecular Identification of *K. pneumoniae*

Genomic DNA from *K. pneumoniae* was extracted using a commercial kit (AddBio, Korea), and the 16S rDNA was amplified using universal primers: 27F (5⁣′-AGAGTTTGATCMTGGCTCAG-3⁣′, where M is A or C) and 1492R (5⁣′-GGYTACCTTGTTACGACTT-3⁣′, where Y is C or T). PCR was performed using a DNA thermocycler (LabTech, Sorisole, Bergamo, Italy) to amplify a ~1500 bp fragment.

The PCR reactions were prepared using AddStart Taq Master, primers, and DNA template in a total volume of 50 *μ*L. The thermal cycler was programmed with the following settings: initial denaturation (95°C, 5 min), 30 cycles of denaturation (95°C, 30 s), annealing (55°C, 30 s), extension (72°C, 30 s), and a final extension (72°C, 5 min). After amplification, the tubes were maintained at 4°C until further processing [28]. PCR products were resolved on 1.5% (*w*/*v*) agarose gel stained with ethidium bromide and visualized under UV light. After gel electrophoresis, the amplified products were packaged and transported to a sequencing facility in Korea. The sequences were analyzed using UGENE software (Version 50.0) [29] and then blasted against the NCBI database. Accession numbers were obtained from GenBank [30].

### 2.3. Antibiotic Susceptibility Testing

Antibiotic sensitivity test was performed for those strains that were identified by the hospitals as strains highly resistant to multiple classes of antibiotics. The sensitivity of these strains was evaluated using automated systems like Phoenix and Vitek 2. Minimal inhibitory concentration (MIC) and qualitative interpretations (susceptible, intermediate, and resistant) were obtained for the following antibiotics: cefepime, ceftriaxone, meropenem, imipenem, amikacin, gentamicin, ciprofloxacin, levofloxacin, and colistin. All tests were performed according to CLSI guidelines [31].

### 2.4. Biofilm Detection Assays

#### 2.4.1. Biofilm Detection Using Congo Red Agar (CRA)

Biofilm-forming strains were initially identified using CRA. The medium was prepared by combining blood agar (40 g/L), glucose (10 g/L), and Congo red dye (0.4 g/L). The dye solution was prepared separately, excluded from the autoclaving process, and incorporated into the medium once it had cooled to 50°C. Each clinical isolate was cultured on the prepared medium, with biofilm-forming strains identified by the production of black crystalline colonies [32].

#### 2.4.2. Quantitative Biofilm Assessment With Crystal Violet Assay (CVA)

Positive biofilm-forming strains (CRA) were quantified using CVA. Overnight, TSB cultures (at 37°C) were diluted to 0.5 McFarland standard (1.5 × 10^8^ CFU/mL) concentration, and aliquots (100 *μ*L) were transferred in triplicate to 96-well plates. Three negative wells were included to establish baseline optical density change (ODc). Plates were then incubated for 24 h (at 37°C). After incubation, the wells were rinsed and dried at room temperature (15 min). Next, 125 *μ*L of methanol was added to each well and incubated for 15 min, then it was removed, and the plates were dried (15 min). Plates were then stained with 125 *μ*L crystal violet and incubated for 15 min. They were rinsed three times with water and dried overnight. Biofilm was dissolved with 125 *μ*L of 30% (*v*/*v*) acetic acid and incubated at room temperature (15 min). OD630 was measured using BioTek ELx808U. Biofilm formation was classified as follows: nonproducer (OD ≤ ODc), weak (ODc < OD ≤ 2 × ODc), moderate (2 × ODc < OD ≤ 4 × ODc), or strong (OD > 4 × ODc) [33].

### 2.5. Molecular Characterization of Biofilm and Antibiotic Resistance Genes

DNA was extracted from 19/51 samples (37.3%) that met the criteria of being multidrug-resistant and biofilm-forming. For these samples, DNA was extracted using colony PCR. Two to three colonies were resuspended in 50 *μ*L of distilled water inside an Eppendorf tube. Then, they were heated at 95°C for 5 min to lyse the cells. The resulting lysate served as the template for the PCR.

#### 2.5.1. Biofilm-Associated Gene Analysis

To further assess biofilm formation capacity, the *mrkA* gene was targeted. Conventional PCR was carried out according to previous protocols with minimal alterations (31). DNA was amplified with the following primers: *mrkA*-F (5⁣′-AAGTTAAAGCGGCAGCAG-3⁣′) and *mrkA*-R (5⁣′-TGTCAGTAGACAGCACCAG-3⁣′). The reaction mixture contained 12.5 *μ*L master mix, 2 *μ*L template, 0.5 *μ*L of each primer, and 9.5 *μ*L dH_2_O. The tubes were then placed in a thermal cycler that had been programmed with the following settings: initial denaturation at 94°C for 2 min, followed by 30 cycles of denaturation at 94°C for 30 s, annealing at 57°C for 30 s, and extension at 72°C for 1 min. The final extension is at 72°C for 5 min. Finally, amplified products were analyzed by 1.5% agarose gel electrophoresis in TAE buffer at 90 V for 45 min [34].

#### 2.5.2. Antibiotic Resistance–Associated Genes Analysis

Antibiotic resistance genes, including *blaSHV*, *blaTEM*, and *blaKPC*, were identified using conventional PCR. The amplification of these genes was performed as follows: Reaction tubes were made as stated in the *mrkA* gene analysis, using the primer set indicated in Table 1. The tubes were then placed in a thermal cycler programmed with the parameters provided in Table 2. The amplified product and DNA ladder were then loaded into an agarose gel (1.5%) with TAE buffer stained with ethidium bromide and run at 90 V for 90 min.

### 2.6. Expression Analysis via Quantitative Reverse Transcription PCR (RT-qPCR)

Bacterial isolates were cultivated in Tryptic Soy Broth with varying pH values (7 and 5) and incubated for 24 h (37°C). pH 5 was chosen to induce biofilm formation, as acidic environments are known to trigger biofilm gene expression as a stress response mechanism. pH 7 served as a control environment. Sample concentrations were standardized to 3.3 McFarland using the corresponding broths. RNA extraction kit (Sangene, China) was then used to extract the total RNA from the samples, according to the manufacturer's protocol.

One-step RT-qPCR was then performed using the SYBR Green Master Mix (AddBio, Korea). Using the aforementioned primers, PCR amplification was performed for *blaSHV*, *blaTEM*, *mrkA*, and *16S rRNA* (internal control). During the amplifications, the Roche LightCycler 96 was used to quantify fluorescence (the thermal cycling program was set according to the manufacturer's protocol). For each gene, contamination was assessed using a no-template control.

Relative gene expression was quantified using 2^−ΔΔCt^ method. First, the raw Ct values for each of the target genes were normalized to the Ct value of the internal control (endogenous control gene 16S rRNA), and the resulting Ct values were used as ΔCt. Then, the ΔCt value of each target gene was normalized to the ΔCt of the control group (pH 7) to determine the ΔΔCt and ultimately the fold change. The ΔCt of *mrkA*-positive and *mrkA*-negative strains was compared to each other in both environmental conditions [38].

### 2.7. Phylogenetic Analysis of Clinical Isolates

To investigate the evolutionary relationships of chosen bacterial strains, a phylogenetic tree was constructed using IQ-Tree (V 2.4.0) and the maximum likelihood (ML) method [39]. Briefly, the 16S rRNA sequences of 17 bacteria (including the five samples sequenced in this study) were aligned using MAFFT (V 7.526) default parameters in the UGENE software [29]. The aligned sequences were manually examined for poorly aligned regions to ensure high-quality data for tree construction, and the poorly aligned end regions were trimmed. The tree was constructed based on the Bayesian information criterion (BIC) model finder within IQ-Tree. GTR + *F* + *R*2 was chosen as the selected model, a bootstrap value of 1000 was used, and the tree was constructed. Finally, the tree was visualized and customized with iTOL applications [40].

### 2.8. Statistical Analysis of Experimental Data

All statistical analyses were performed using R (V 4.4.2) and Python (V 3.12). The statistical significance threshold was set at *p* < 0.05. The results were then summarized in tables and visualized using the R packages ggplot2 and ggstatsplot [41]. The dataset was first assessed for normality using the Shapiro–Wilk test and Q–Q plots, and depending on the distribution of data, parametric or nonparametric tests were used [42]. Comparative statistical analysis included one-way and two-way ANOVA for normally distributed data and the Kruskal–Wallis test for nonnormally distributed data. Fisher's exact test was used to explore the relationship between the categorical columns. The Mann–Whitney *U* test was used to explore the distribution of the data between two groups, while the Wilcoxon signed-rank test was used for in-group comparison. Effect sizes were calculated using rank biserial correlation to provide insights into the practical significance of observed differences. Empirical mode analysis (EMA) was applied to explore patterns and relationships in complex datasets.

## 3. Results

### 3.1. Collected Samples

During the 3-month sampling period, 51 samples were gathered from patients. Of these 51 samples, 20 (39.2%) were from male patients, whereas 31 (60.8%) were collected from female patients. The most common form of infection seen in these patients was urinary tract infection (UTI), accounting for 26 (50.98%) of the 51 cases. The patients' ages ranged from 1 month to 84 years old. The majority of patients in this study were above the age of 40. Among these patients, UTI was more common in females than in males, and most were under 30 years old. Notably, UTI was uncommon in older and senior individuals. Respiratory, wound, and tissue infections were also present among these patients, but no significant relations were found.

### 3.2. Identification of Clinical Isolates

The selected sample sequences were compared to reference sequences from NCBI GenBank. The isolates showed high similarity to the reference genes in the NCBI database, and they were identified as *K. pneumoniae* based on sequence homology. Following sequence comparison, each sequence was submitted to NCBI GenBank and received an accession number (Table 3).

### 3.3. Antibiotic Susceptibility Test Results

**Figure 1:**
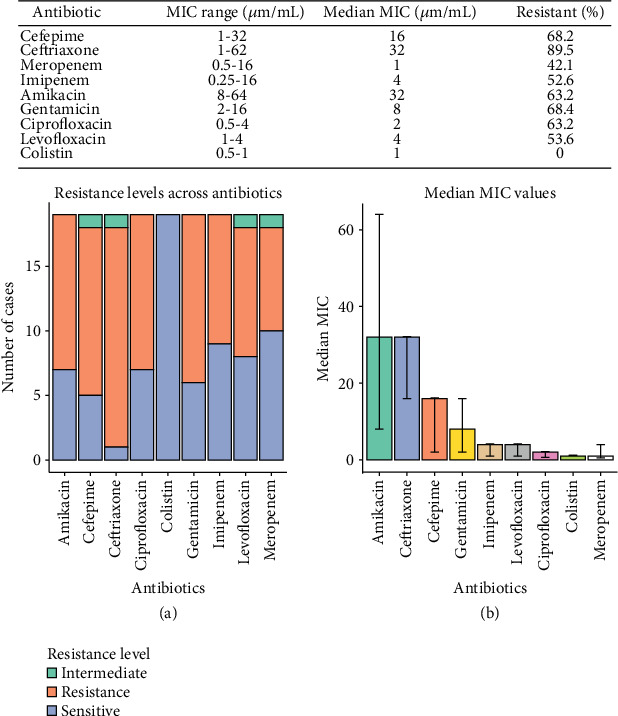
MIC ranges, medians, and resistant percentages for the tested antibiotics. (a) The plot shows the count of resistance (orange), intermediate (green), and sensitive (blue) strains for each antibiotic, and (b) a bar plot illustrates the MIC_50_ values for each antibiotic with error bars representing the interquartile range (IQR).

Based on the initial antibiotic sensitivity test, only resistant bacterial strains (MDR and above) were chosen for further testing, with 19 out of 51 clinical samples selected. The MIC revealed that ceftriaxone exhibited the highest resistance, with 89% of the isolates classified as resistant. This was followed by gentamicin (68.4%), ciprofloxacin (68.2%), and cefepime (68.2%). Colistin was the most effective antibiotic against all the tested isolates, and no resistant strains were present against it. Several noteworthy findings are that the resistance toward *β*-lactam antibiotics is more commonly seen in patients aged over 41, possibly showing a resistance trend seen in the older population. At the same time, this age group has also been prescribed colistin the most. The younger population also showed high resistance toward *β*-lactam antibiotics, but it was lower compared to the older population. Patients aged between 31 and 40 years had the lowest resistant count in all the observed groups. These results imply a resistant trend in the older and younger patients, whereas adults are less affected by this resistant trend. Further research should be carried out to explore this trend and uncover the reasons for it. These results are highlighted in Figure 1.

### 3.4. Biofilm Formation Assessment

All clinically isolated samples were found to be biofilm formers using both CVA and CRA. Twelve (23.5%) out of the 51 samples were categorized as weak biofilm formers, 34 (66.7%) as moderate biofilm formers, and just five (9.8%) as strong biofilm formers. It is also important to note that all the strong biofilm formers were identified exclusively from female patients. The average OD measurement across the samples was 0.183 ± 0.037. Baseline ODc was set at 0.064 ± 0.005; samples with higher readings were considered positive, while lower readings were considered negative for biofilm formation. The significant proportion of moderate biofilm formers warrants additional investigation, implying a role in clinical persistence and pathogenesis, particularly given their likely association with recurrent or chronic infections.

**Figure 2:**
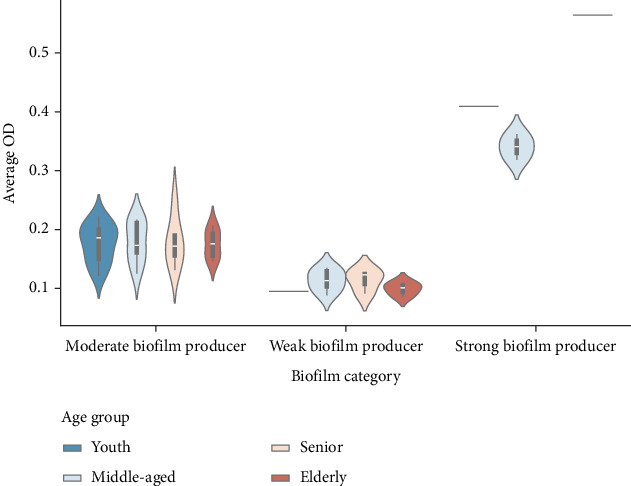
Distribution of biofilm biomass by patient age group. Isolates were grouped based on the patient's age category (youth, middle-aged, senior, and elderly). The violin plots show the distribution of the optical density in each age group that was subgrouped by the biofilm category. The width of the violin plots indicates the frequency of the isolates at that OD value. The black bar inside the violin plots represents interquartile ranges, and the white dots are the median value for that age group. Single lines indicate one sample per that age group.

As illustrated in the violin plot (Figure 2), the distribution of OD in moderate biofilm formers varies substantially between age groups, with the maximum variability reported in the youth and senior population. This could imply heterogenicity in the strains' biofilm-forming capacities, indicating either diverse bacterial strains or patient-specific factors. Another notable discovery was the absence of weak biofilm formers among individuals under the age of 30, which could reflect the rarity of these strains in younger populations. Strong biofilm producers, although limited in number, have highly focused and elevated OD values, distinguishing them as a unique phenotypic trait with little overlap with the moderate or weak categories. These findings highlight the need for further research into the age-related factors that contribute to and influence biofilm formation.

#### 3.4.1. Prevalence of the *mrkA* Gene Among Biofilm-Forming Isolates

**Figure 3:**
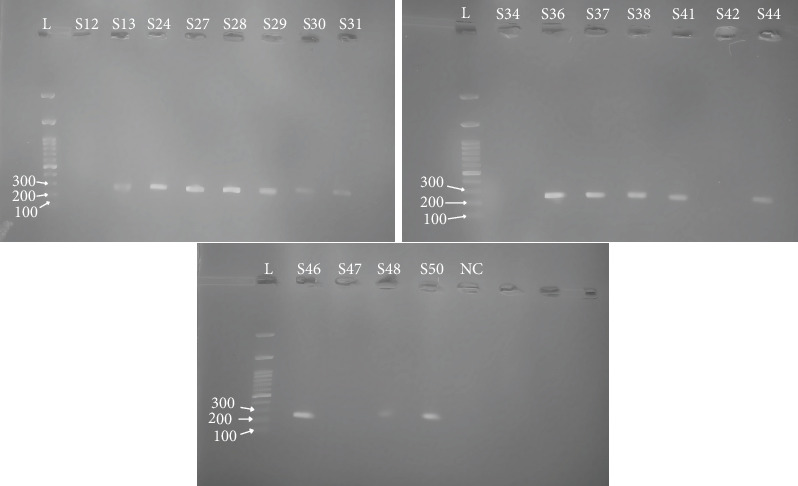
The gel image of the *mrkA* PCR product shows distinct bands at the expected band size of 289 bp; the gel also contains a 100 bp DNA ladder and a negative control (NC). (Full unprocessed gel images are available in the supporting information.)

**Figure 4:**
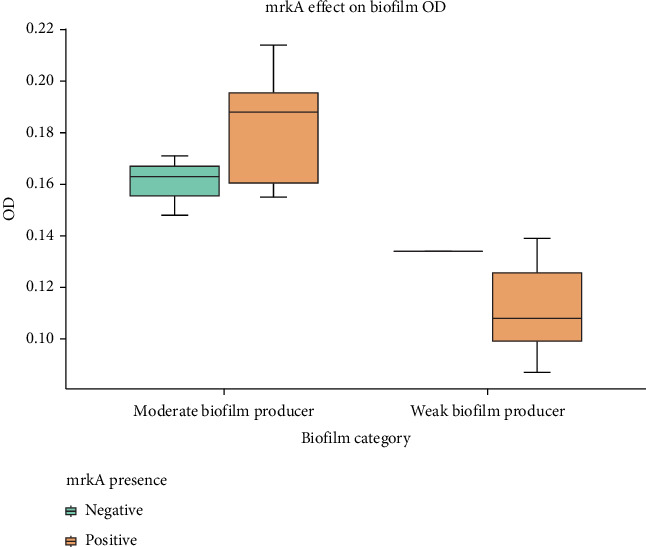
Box plot depicting the optical density (OD) distribution of moderate and weak biofilm producers. Categorized by the *mrkA* gene presence (positive/negative). Orange box plots show strains with the *mrkA* gene, whereas green box plots show *mrkA*-negative strains; one individual data point is shown as a line.

Of the selected samples, 15 (78.9%) were positive for *mrkA* genes, while 4 (21.1%) were negative (Table 4), as validated by the gel electrophoresis, showing a clear band at the expected band size of 289 bp (Figure 3). The majority of the samples that tested positive for the *mrkA* gene were moderate biofilm formers, with only five classified as weak biofilm formers (Table 5). However, as shown in the boxplot (Figure 4), isolates that tested positive for the *mrkA* gene had much greater OD readings than those that tested negative. Strains carrying *mrkA* genes had a median OD reading of 0.18, while those lacking *mrkA* had a median OD value of 0.16 (Figure 4). The box plot also shows a minimal overlap between *mrkA*-positive and *mrkA*-negative strains within the “Moderate biofilm producer” category, demonstrating distinct phenotypic traits associated with the presence of *mrkA*. These data imply that the presence of the *mrkA* gene is associated with a unique phenotype.

### 3.5. Prevalence of the *blaSHV*, *blaTEM*, and *blaKPC* Genes Among the Clinical Isolates

**Figure 5:**
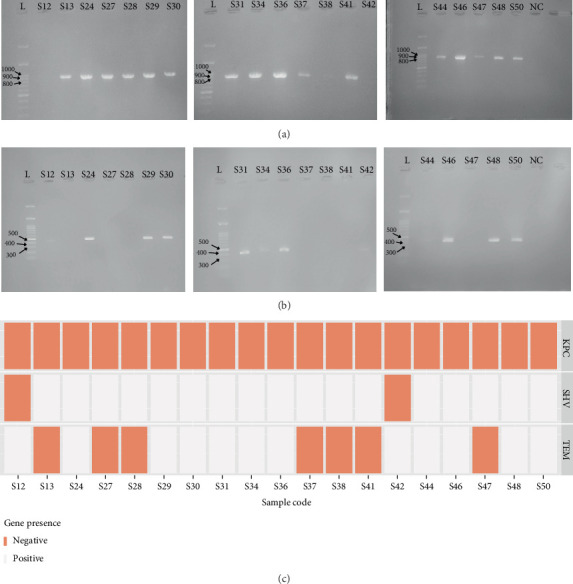
Gel electrophoresis results show PCR amplification of (a) *blaSHV* and (b) *blaTEM* with a 100 bp DNA ladder. Both *blaSHV* and *blaTEM* show clear bands at expected band sizes of 856 and 403 bp. (c) The presence of the genes among the 19 clinical isolates: White bars indicate positive and orange bars correspond to strains lacking the gene; on the *x*-axis, letter S represent sample; and the number corresponds to the sample code. (Full unprocessed gel images are available in the supporting information.)

The gel electrophoresis of *blaSHV* and *blaTEM* produces distinct bands at expected sizes of 865 and 403 bp, respectively, while *blaKPC* gel images show no visible bands, indicating the absence of the gene among the isolates. Eighty-nine percent (17 out of 19) had *blaSHV*, and sixty-three percent (12 out of 19) had *blaTEM* (Figure 5). As shown previously with the AST results, the high resistance cases for beta-lactam antibiotics might be explained by the high prevalence of the *blaTEM* and *blaSHV* genes.

### 3.6. Real-Time PCR

The expression of biofilm and antibiotic resistance genes was evaluated in four samples under two different environmental conditions. Based on the results, both the antibiotic and biofilm genes are more expressed at pH 5 compared to pH 7. Overall, bacterial strains with the *mrkA* gene express more antibiotic resistance genes than strains without the gene. The expression of *blaSHV* in *mrkA*-positive strains exhibited an average ΔCt value of −0.07 in acidic settings and 3.72 in neutral pH. This was higher than the *mrkA*-negative strains, which had an average ΔCt value of 5.85 in acidic conditions and a mean value of 20.93 in normal conditions.

Furthermore, the expression of *blaTEM* in bacterial strains carrying the *mrkA* gene had a ΔCt value of −2.72 in the acidic medium, whereas it was expressed at a lower level at normal pH with a ΔCt value of −0.35. When compared to *mrkA*-negative bacteria, a similar pattern of *blaSHV* gene expression is seen. At pH 5, it had a ΔCt value of 12.46, whereas at pH 7, the ΔCt value was 24.59 (not expressed).

**Figure 6:**
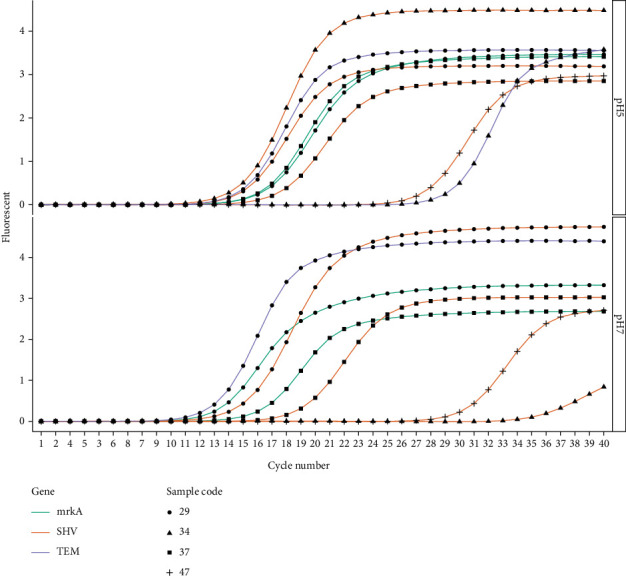
Representative amplification plots from the RT-qPCR analysis. It shows fluorescence on the *y*-axis and the cycle number on the *x*-axis. The plot is faceted by pH conditions. Each gene has a distinct color: *mrkA* (green), *blaSHV* (orange), and *blaTEM* (blue), and the samples are labeled with different shapes: Sample 29 (circle), Sample 34 (a triangle), Sample 37 (a rectangle), and Sample 47 (a plus sign).

**Figure 7:**
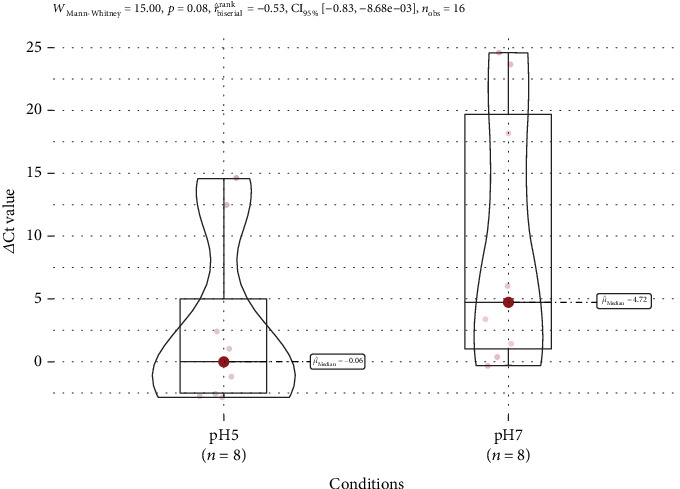
Box and violin plot comparison of ΔCt values between two pH conditions. The individual data points are shown in a light beige color. The box plots show the interquartile range, and the red dot indicates the median value. The width of the violin plots depicts the frequency of the ΔCt values within each pH condition.

These results imply that the presence of the *mrkA* gene may result in a higher expression of *blaSHV* and *blaTEM*. They also demonstrate that both antibiotics and biofilm genes are more expressed in acidic environments than in normal pH. Tables 6 and 7, as well as Figures 6 and 7, provide more illustrations of these findings.

### 3.7. Phylogenetic Analysis of Clinical *K. pneumoniae* Strains

**Figure 8:**
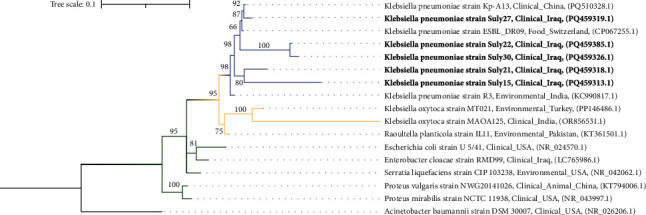
Phylogenetic tree analysis for the sequenced samples. Each branch is shown with its bootstrap value, with the scale indicated above the branches. The strains used to construct the tree are grouped by their families, and the study samples are highlighted in bold. *Acinetobacter baumannii* was used to root the tree.

The phylogenetic tree was constructed using the IQ-Tree and iTOL programs, using the ML method. Based on BIC values, the model GTR + *F* + *R*2 was employed to construct the tree; the findings demonstrate a clear grouping of the *K. pneumoniae*, indicating genetic similarities. The five clinical isolates evaluated in this study (Suly15, Suly21, Suly22, Suly27, and Suly30) share genetic similarities with other *K. pneumoniae* strains from various geographical areas, including China, Switzerland, and India. This similarity is statistically supported by strong bootstrap values, as illustrated in Figure 8. The tree also includes other reference genomes, such as *E. coli* and *Serratia*, which provide a more comprehensive evolutionary background for the investigation.

### 3.8. Statistical Analysis

**Figure 9:**
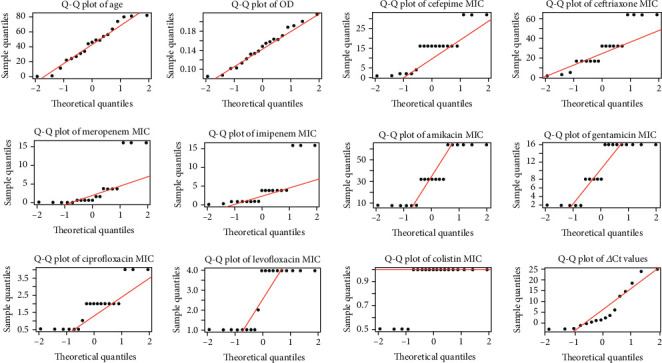
Quantile–quantile plots. These plots compare the distribution of the sample against theoretical quantities. The *x*-axis shows the theoretical quantiles, while the *y*-axis shows sample quantiles. The solid red line is the reference line of identity; data points that fall closely along this line suggest a normal distribution.

To verify the dataset's normality assumption, the Shapiro–Wilk normality test and Q–Q plot were performed (Table 8). Most of the *p* values were less than 0.05, indicating a rejection of the null hypothesis and suggesting a nonnormal distribution, except for the columns “Age” and “OD,” where the *p* values are above the 0.05 threshold. Normally, numerical values beyond this level are deemed normal in this situation, due to the small sample size. The accuracy of the normality test is lower, and the final decision about normality was based on the Q–Q plots given in Figure 9. The graphs clearly show a nonnormal distribution pattern for the “Age” column, while OD values show a normal distribution; hence, the dataset was assumed to be nonnormal except for the OD values. Based on this conclusion, nonparametric and parametric tests were used to further analyze the dataset.

Kruskal–Wallis's rank sum test showed that there was no statistically significant difference in the age of the patients and the type of infections (Table 9). There was also no significant difference between the patients' age and their gender.

The findings from Kruskal–Wallis's test (Table 10) suggest that there is a nonsignificant difference in the MIC values of the antibiotics across gender and infection type. Age also had no significant difference across the groups (Table 9). This suggests that the resistance profiles of the species in the study are relatively consistent across the groups, and it implies that the strains might have a common origin. Also, the MIC values of antibiotics do not differ much when *blaSHV* and *blaTEM* genes are present or not. This suggests that resistance to *β*-lactam antibiotics may also be influenced by other factors, like other resistance genes that were not tested in this study.

That being said, the *p* value for ciprofloxacin when compared across *blaSHV* presence is marginally significant (*p* = 0.063). This is also the same case for gentamicin. MIC variation when compared across *blaTEM* presence is marginally significant (*p* = 0.068). Although they are not statistically significant, this implies that these genes might have an impact on the resistance of bacteria toward these antibiotics specifically, either directly or indirectly. Further studies and a larger sample size could clear this confusion.

Based on the results of the ANOVA test (Table 11), there seems to be no significant effect on OD when its values are compared among the different groups in the dataset. However, this does not align with the previous finding that suggests that strains with *mrkA* genes tend to have higher OD. For this purpose, the data was further analyzed with two-way ANOVA, as shown below in Table 12.

The results of the two-way ANOVA suggest that the biofilm category OD differs greatly from each other; for this purpose, Tukey's test was carried out. It revealed that moderate biofilm formers have, on average, higher OD with a value of 0.0618 when compared to weak biofilm formers; this was also statistically significant with a *p* value of 5.8e − 06. The model also suggests that the *mrkA* effect on the biofilm category is marginally significant, so for this, estimated marginal means (EMMs) and pairwise contrasts were used to further explain this variation. The adjusted OD mean in moderate biofilm formers revealed that *mrkA*-negative strains have a mean OD of 0.162, while *mrkA*-positive strains have a mean OD of 0.180. This results in a 0.018 difference in the OD mean between the two categories, which is not statistically significant.

**Figure 10:**
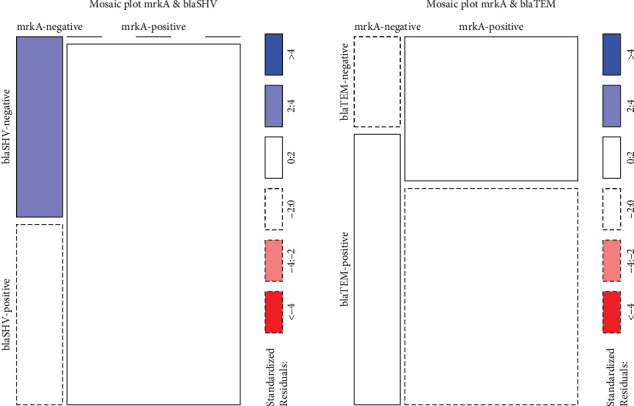
Mosaic plot that explores the relationship between *mrkA*, *blaSHV*, and *blaTEM* genes. The areas of each tile represent the number of cases in each category. Darker shading indicates stronger evidence against independence.

Fisher's exact test was conducted to examine the associations between the antibiotic resistance genes and the *mrkA* gene. The results suggest a borderline significant (*p* = 0.03509) association between *mrkA* and *blaSHV* genes. This suggests that the presence of *mrkA* is not independent of the *blaSHV* gene; specifically, strains with the *mrkA* gene are more likely to have the *blaSHV* gene and vice versa. A larger sample size is needed for confirmation and to conclusively determine if these relations are real or due to random chance. On the other hand, there was no statistically significant difference between *mrkA* and *blaTEM* genes. These findings suggest a potential co-occurrence or linkage between *mrkA* and *blaSHV* and no direct linkage between *mrkA* and *blaTEM* genes (Table 13 and Figure 10). This finding may provide preliminary support for the hypothesis that beta-lactamase genes are more widely distributed among biofilm-forming strains.

The results of the Mann–Whitney *U* test showed several significant findings; it showed that the ΔCt value for the antibiotic resistance genes in strains with *mrkA* (median: −1.18) was significantly lower than that of strains lacking *mrkA* (median: 12.46). This result was found to be statistically significant (*W* = 0, *p* = 0.03571, *r* = 0). The reason for the complete separation in the values reflects minimal or undetectable levels of gene expression among the strains lacking the *mrkA* gene under neutral conditions. In contrast, the results at pH 5 were found to be nonstatistically significant (*W* = 5, *p* = 0.5714, *r* = −0.2635) between strains having the *mrkA* gene and strains lacking it. The small effect size indicates no meaningful changes between the two groups.

Although the results for the Mann–Whitney *U* were not significant for the expression levels at pH 5, there is a clear trend in the data that shows that strains with the *mrkA* gene have higher expression compared to strains without *mrkA*; this statistical test might be significant with a larger sample size (Table 14).

Lastly, expression of the genes was evaluated across the different pH levels. Wilcoxon's signed-rank test showed significant findings (*z* = −2.0926, *p* = 0.03639, *r* = −0.7398). This further confirms that both antibiotic and biofilm genes are more greatly expressed in an acidic environment compared to natural conditions (Table 14), further strengthening the findings of Figure 8.

**Figure 11:**
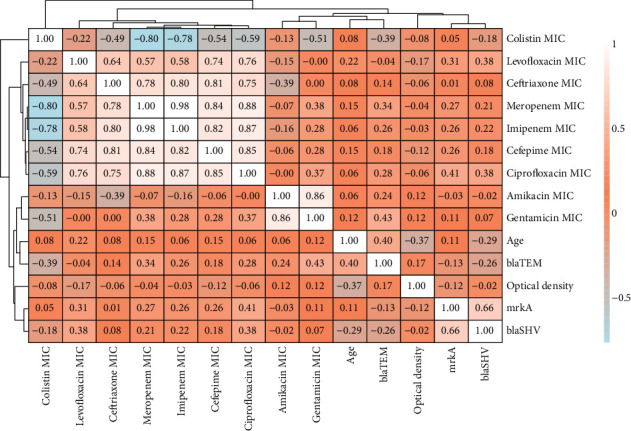
Spearman's correlation matrix shows the correlation strength for numeric columns. The presence of resistance genes and biofilm genes was converted into binary (0 and 1) for matrix formation. Color gradients represent the correlation coefficient, ranging from −1 to +1. Lighter colors (light beige and blue) indicate stronger relations, while darker orange colors show weaker relations.

The heat map (Figure 11) illustrates several key findings, a strong positive association (*r* > 0.80) between *β*-lactams such as cefepime, ceftriaxone, meropenem, and imipenem. This positive relationship supports the existence of extended-spectrum beta-lactamases (ESBL), although it could also be related to the presence of carbapenems. Ciprofloxacin and levofloxacin have a positive relationship with *β*-lactams, ranging from 0.57 to 0.88. This could indicate cross-resistance between these antibiotic classes.

Colistin has a negative connection with practically all the studied antibiotics, which is understandable given that it is a last-resort antibiotic. This is consistent with its distinct mechanism of action and reserved use. Interestingly, age has a weak positive link with some antibiotics, such as levofloxacin (*r* = 0.22) and cefepime (*r* = 0.15), which may indicate age-related trends in antibiotic resistance or usage patterns. These findings warrant further exploration to elucidate their clinical implications.

Furthermore, the resistance gene *blaTEM* has a weak negative correlation with colistin (*r* = −0.39). This might suggest that the presence of the gene has a potential inverse relation with colistin. Also, biofilm formation seems to have a weak negative correlation with levofloxacin (*r* = −0.17) and a weak positive correlation with the *blaTEM* gene (*r* = 0.17). The *blaSHV* and *mrkA* genes have a positive relation (*r* = 0.66); this might indicate that strains with one of these genes may also carry the other gene, further supporting the findings of the Fisher test. There are also many other relations in the heat map, but due to the limited sample size, these relations need to be confirmed with further research and larger sample sizes.

**Figure 12:**
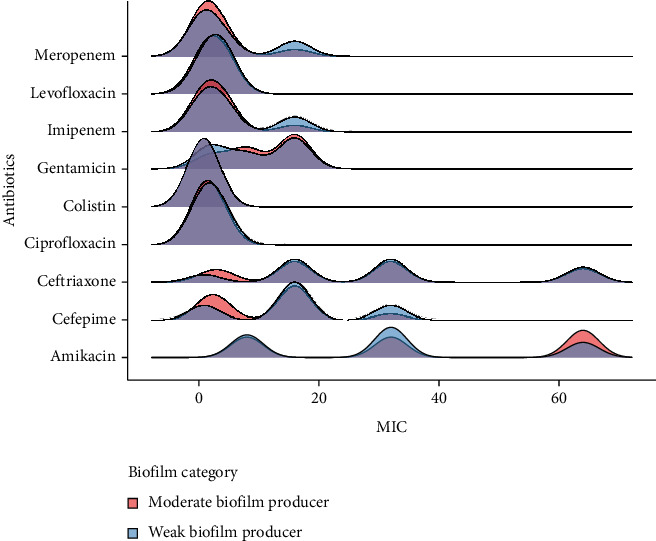
Distribution of MIC values across antibiotics in moderate and weak biofilm producers. The ridge density plot displays moderate biofilm formers in orange and weak biofilm formers in blue. Areas with purple ridges are an overlay of both categories.

As shown in Figure 11 (correlation heat map), the resistance level of amikacin and gentamicin showed a strong positive correlation (*r* = 0.84). To further investigate this relationship, a ridge density plot was created. The results indicate no significant association between biofilm-forming capabilities and antibiotic resistance. However, in the case of gentamicin and amikacin strains that exhibit higher MIC values, they tend to be moderate biofilm formers (Figure 12).

Conversely, for other antibiotics like meropenem and imipenem, there seems to be a negative correlation, as higher resistance has been seen in strains that are weak biofilm formers. These findings suggest that biofilm production might provide specific resistance to some antibiotics, while others are not as impacted. This might be due to the antibiotic mechanism of action. Alternatively, it also might be due to a limited sample size.

## 4. Discussion

This study investigated the relationship between the biofilm-associated gene (*mrkA*) and the antibiotic resistance genes (*blaSHV* and *blaTEM*) in clinically isolated *K. pneumoniae*. The isolates were systematically analyzed for their biofilm-forming ability, as well as the presence of antibiotic resistance and biofilm-related genes. Statistical analysis revealed several key associations, notably a potential trend toward higher prevalence of antibiotic resistance genes in *mrkA*-positive strains (*p* = 0.03509, *r* = 0.66). This potential association may suggest that antibiotic resistance genes are more widely spread in biofilm communities compared to solo roaming cells. These outcomes provide valuable insights into the linkage between biofilm-associated genetic factors and AMR, emphasizing the need for further research to elucidate the underlying molecular mechanisms.

The antibiotic resistance profile of the isolated samples reveals a concerning prevalence of MDR, XDR, and PDR strains. Significant resistances were seen against *β*-lactam antibiotics such as ceftriaxone, gentamicin, ciprofloxacin, and cefepime, possibly indicating the high prevalence of ESBL strains in the isolated samples. The presence of ESBL-producing strains is further supported by the high correlation value (*r* > 0.80) between the *β*-lactam MIC values. Comparable findings have been observed by Müller-Schulte et al. [43], who reported a high prevalence of ESBL-producing strains in hospital environments, highlighting the growing burden of multidrug-resistant pathogens in clinical settings.

Colistin resistance was not observed in any of the isolated samples, which is consistent with the findings of Sharma et al. [44]. This aligns with current knowledge regarding colistin's unique mechanism of action, which involves disrupting the outer membrane of Gram-negative bacteria and its classification as a last-resort antibiotic [44]. The absence of colistin resistance in this study may reflect its restricted use in clinical practice, which helps preserve its efficacy. As shown in the correlation matrix, colistin shares an inverse relationship (−0.13 < *r* < −0.80) with all the other antibiotics, further supporting this claim. However, continued surveillance is essential, given the emerging reports of colistin-resistant strains due to the acquisition of *mcr* genes, which pose a significant threat to antibiotic stewardship efforts.

The observed weak positive correlation between patient age and resistance to antibiotics such as cefepime and levofloxacin suggests a potential trend of increasing antibiotic resistance among older populations. This observation aligns with findings from Theodorakis et al. [45], which reported higher resistance to *β*-lactam antibiotics in elderly patients. Several factors may contribute to this trend, including immunosenescence, the natural decline in immune function with age, and the increased prevalence of comorbidities among the elderly, leading to more frequent healthcare interactions and antibiotic exposures [46]. These factors collectively heighten the risk of infections caused by multidrug-resistant organisms in older patients [46]. Additionally, the elderly often reside in long-term care facilities, environments that can facilitate the spread of resistant bacteria due to close living quarters and communal activities [47]. Therefore, the association between age and increased antibiotic resistance underscores the need for targeted antimicrobial stewardship and infection control measures within this vulnerable population.

All the studied isolates exhibited biofilm-forming capability, suggesting a potential role in biofilm-associated infection within the studied population. Notably, the majority of strains (66.7%) were categorized as moderate biofilm formers, a finding that aligns with the study by Guerra et al. [48], which reported a high prevalence of biofilm-forming clinical isolates. This suggests that biofilm formation might be a common trait of bacterial strains isolated from clinical environments. An intriguing observation was that all strong biofilm-forming (9.8%) isolates were exclusively recovered from female patients with UTIs. This finding raises the possibility of a gender-related influence on biofilm formation, which may be mediated by differences in the host microbiota, immune system responses, or hormonal regulation, as suggested by Hammouda et al. [49].

Additionally, correlation analysis revealed a weak negative association between patient age and OD values (*r* = −0.37), suggesting that younger individuals are more likely to harbor moderate and strong biofilm-forming strains. The observed results may be attributed to age-related differences in host–pathogen interactions, immune responses, or bacterial adaptation strategies [46]. Aging leads to a decline in immune system function, known as immunosenescence, which compromises both innate and adaptive responses, including impairments in phagocytosis and decreased production, activation, and function of T and B lymphocytes [45]. Bacteria exploit immunosenescence by using various virulence factors to evade the host's defenses, leading to severe and often life-threatening infections [45]. Additionally, age-related changes in the microbiota can influence host–pathogen interactions, potentially affecting bacterial adaptation and biofilm production [50].

Seventy-nine percent of the clinical isolates tested positive for the *mrkA* gene. Strains harboring this gene exhibited higher OD values (${\bar{x}}^{\text{pos}}=0.180$) compared to those lacking it (${\bar{x}}^{\text{Ngs}}=0.164$), which is in line with findings from existing studies [51]. This observation demonstrates the role of the *mrkA* gene, which encodes a major subunit of Type 3 fimbriae, important in promoting biofilm formation. Type 3 fimbriae are known to facilitate bacterial adherence to surfaces, a critical step in biofilm development and the persistence of infections [51]. These results further highlight the significance of the *mrkA* gene in the pathogenicity and clinical relevance of biofilm-forming bacterial strains.

Although statistical analysis (two-way ANOVA and EMMs) did not reveal significant differences between *mrkA*-positive and *mrkA*-negative strains, the mean OD values between the two groups suggest a potential distinction otherwise. The data shows that *mrkA*-positive strains exhibited an average OD of 0.18, while the *mrkA*-negative strains had a mean OD of 0.16, indicating a higher biofilm-forming potential in strains with the *mrkA* gene. This highlights a possible functional difference between the two groups, despite the lack of statistical significance. Moreover, a weak positive correlation (*r* > 0.27) was observed between the presence of the *mrkA* gene and the MIC values for levofloxacin, ciprofloxacin, imipenem, and meropenem. This suggests that strains harboring the *mrkA* gene may have improved survival capabilities, possibly due to enhanced biofilm formation. These findings are consistent with the relationship reported by Gual-de-Torrella et al. [52], which also highlighted the role of *mrkA* in improving bacterial survival under antibiotic stress.

The *mrkA* gene encodes the major structural subunit of Type 3 fimbriae in *K. pneumoniae*, which are hair-like appendages extending from the bacterial surface [53]. These fimbriae play a crucial role in the initial adhesion of bacteria to host tissues and abiotic surfaces, facilitating the formation of biofilms. Studies have demonstrated that the expression of Type 3 fimbriae, including the *mrkA* protein, is essential for efficient biofilm formation in *K. pneumoniae* strains [53]. Furthermore, the regulation of *mrkA* expression is influenced by various factors, including the presence of cyclic-di-GMP, a secondary messenger molecule that modulates biofilm formation in many bacteria. Research has shown that the transcriptional activator *mrkH*, which binds to the *mrkA* promoter region in the presence of cyclic-di-GMP, enhances the expression of Type 3 fimbriae and promotes biofilm formation collectively [54, 55]. These findings demonstrate the significant role of the *mrkA* gene and its encoded fimbrial subunit in the biofilm-forming capacity of *K. pneumoniae*, suggesting its potential as a target for therapeutic interventions aimed at disrupting biofilm-associated infections.

Molecular identification of resistance genes revealed a high prevalence of *blaSHV*, which was present in 89% of the isolates, while *blaTEM* was identified in 63% of the isolates. Interestingly, the *blaKPC* gene was absent in all the examined samples, a finding consistent with those reported by Ojdana et al. [56]. Recent surveillance studies conducted in Iraq and neighboring countries have shown that the dominant carbapenemases in *K. pneumoniae* are *blaOXA-48* and *blaNDM*, with *blaKPC* being detected rarely, if at all [57]. For instance, a study conducted in Baghdad hospitals showed that only 20% of the isolates of *K. pneumoniae* carried the *blaKPC* gene, and a higher prevalence rate was seen for *blaOXA-48* and *blaVIM* [58]. Similar studies that were conducted in Kashan, Iran, reported that only 11% of the isolated *K. pneumoniae* carried the *blaKPC* gene [59]. While isolates from Egypt show a much higher prevalence of the *blaKPC* gene, it was observed that 70% of the isolates carried the gene [60]. Collectively, these findings are consistent with the absence of the *blaKPC* gene in this study, and they suggest that while *blaKPC* is present in Iraq and neighboring countries, it is present at lower frequencies compared to other *β*-lactamase genes. Furthermore, while carbapenem resistance was detected in 54% of the isolates, this study indicates that the resistance is associated with the presence of the *blaNDM* gene [61]. This highlights the growing concern over carbapenem resistance and underscores the importance of monitoring resistance patterns in clinical isolates to inform treatment strategies.

Although Kruskal–Wallis's test revealed no significant correlation between the resistance gene and the tested antibiotics, there was a mild significance (*p* = 0.063) with ciprofloxacin for *blaSHV*. This is further illustrated in the correlation matrix, which shows that some antibiotics, such as ciprofloxacin, have a weak positive connection (*r* = 0.38) with the *blaSHV* gene. This suggests that the presence of *blaSHV* causes greater resistance to ciprofloxacin [62]. The presence of *mrkA*, *blaSHV*, and *blaTEM* genes in most isolates indicates a connection between biofilm formation and *β*-lactamase resistance. A positive correlation between *mrkA* and *blaSHV* (*r* = 0.66) suggests that biofilm-forming bacteria are more likely to harbor resistance genes, an association that approached statistical significance (*p* = 0.03509) in the Fisher exact test. However, the modest negative correlation with *blaTEM* may indicate different resistance mechanisms. This finding is concerning, as biofilm-associated bacteria can protect and propagate resistance genes, facilitating the spread of antibiotic resistance in clinical settings. Effective infection control and biofilm disruption strategies are crucial to combat the spread of resistant strains [63].

RT-qPCR analysis has yielded significant insights into the expression patterns of *β*-lactamase genes, particularly *blaSHV* and *blaTEM*, concerning the presence of the *mrkA* gene and environmental pH conditions. Specifically, in *mrkA*-positive bacterial strains, there is a notably higher expression level of both *blaSHV* and *blaTEM* genes. Conversely, in *mrkA*-negative strains, both *blaSHV* and *blaTEM* are less expressed. The normalized ΔCt values showed that in *mrkA*-positive strains, *blaSHV* has an average ΔCt value of −0.07 at pH 5 and 3.72 at pH 7. When compared to *mrkA*-negative strains (5.85 at pH 5 and 20.93 at pH 7), expressions are much higher with *mrkA*-positive strains.

The difference in expression level among *mrkA*-negative strains may be explained by a wider stress response. Both genes are activated in response to environmental stress, such as pH imbalances, to improve survival [64]. Under natural conditions, due to the lack of external stress, the genes are expressed at low levels or not at all. These patterns are presumably driven by differential control of gene promoters, global regulators such as two-component systems, and the adaptive benefits of adjusting *β*-lactamase expression to the environment [65]. These findings suggest that *mrkA* might have a role in modulating *β*-lactamase expression and highlight the influence of environmental factors on antibiotic resistance mechanisms, warranting further investigation into the regulatory pathways governing these adaptations.

Further investigation reveals that with both strains, all the studied genes are expressed at a higher level at pH 5 rather than neutral conditions. These findings are consistent with existing literature that underscores the influence of environmental factors, such as pH, on the expression of *β*-lactamase genes and biofilm genes [64]. Furthermore, it has been observed that often *K. pneumoniae* infections occur in urine, where pH values are often around 6.2. Experimental models conducted on mice have shown that acidifying urine to around 5.0–5.5 pH results in higher growth and colonization of the bacteria; this increases the colonization of the kidney and systemic inflammation [66]. Thus, simulating growth at pH 5 in vitro captures conditions reflective of both the acidic intracellular environment in macrophages and acidic urinary niches during UTIs. The observed differential expression patterns concerning pH and *mrkA* status suggest complex regulatory mechanisms governing *β*-lactamase gene expression [67].

Furthermore, statistical analysis reveals several significant findings. The Mann–Whitney *U* test showed a significant (*p* = 0.037) distribution in the ΔCt value at pH 7 between *mrkA*-positive and *mrkA*-negative strains. This finding is due to the lack of resistance gene expression among the *mrkA*-negative strains, which was further supported by the complete separation of the data (*W* = 0). Also, the Wilcoxon signed-rank test showed that expression levels are much higher in acidic conditions when compared to natural pH levels (*z* = −2.0926, *p* = 0.03639, *r* = −0.7398). These findings further support the theory that under environmental stress (low pH), resistance genes might be upregulated to increase survival rate. Overall, the study data imply that the *mrkA* gene may influence the expression of *β*-lactamase genes, resulting in greater expression of the resistance gene under environmental stress.

The ML phylogeny, rooted with *Acinetobacter baumannii* (outgroup), revealed several key findings. The tree shows a clear clustering of the *K. pneumoniae* strains (BS > 66%). It shows that the studied strains share genetic similarities with other *K. pneumoniae* strains from Iran, China, Switzerland, and India. Particularly, strain Suly27 shows close clustering with other *K. pneumoniae* strains from China and Switzerland. The tree also highlights close grouping of the studied strains under the same leaves, further supporting the Kruskal–Wallis test results, which found no significant variation among the strains, possibly indicating a common origin. The tree also includes species from the Enterobacteriaceae family to illustrate the genetic relationship and evolutionary history.

While this study provides valuable insights into the relationship between biofilm and antibiotic resistance genes, certain limitations should be acknowledged. Notably, the relatively small sample size may restrict the generalizability of the findings by limiting the statistical power and increasing the potential for Type II error. Therefore, the conclusions drawn from these experiments, particularly those approaching statistical significance, should be interpreted with caution. We propose that our findings serve as an important preliminary data that warrants future, larger scale studies.

Furthermore, the study only focused on particular genes; other antibiotic resistance genes and biofilm genes could also play a role in this regulation. Future research should focus on exploring other resistance and biofilm genes and regulatory mechanisms between these genes using transcriptomics and proteomics approaches. Additionally, understanding how environmental factors affect quorum sensing and, in turn, modulate the expression of the genes could lead to new therapeutic targets.

## 5. Conclusion

This research shed light on the relationship between biofilm and antibiotic resistance genes. The findings of the study imply that biofilms not only serve as a stress response mechanism to antibiotics and environmental stress, but they may also regulate antibiotic resistance genes. The implications of this study are twofold. First, it provides information on the relationship between antibiotic resistance and biofilm genes. Second, it stresses the importance of developing novel target therapies that can target these regulatory processes to battle biofilm-associated infections.

Despite the study's contributions, it had several limitations, most notably the number of genes studied and the small sample size. Future research should include a broader examination of biofilm and resistance genes, as well as a larger sample size to generalize the findings to a greater extent. Furthermore, the efficacy of antibiofilm agents in combination with existing antibiotic therapy should be investigated, particularly in clinically isolated strains. Finally, this work emphasizes the relevance and urgency of developing innovative therapies that can target these regulatory pathways. By focusing on the interactions between biofilm and resistance genes, it may be possible to develop more effective treatment approaches for multidrug-resistant infections.

## Figures and Tables

**Table 1:** Primer set used for antibiotic resistance gene characterization.

**Gene**	**Forward and reverse**	**Primers for antibiotic resistance genes (ARGs)**	
		**Sequence**	**Reference**
*blaSHV*	Forward	5⁣′-ATGCGTTATATTCGCCTGTG-3⁣′	[35]
	Reverse	5⁣′-GTTAGCGTTGCCAGTGCTCG-3⁣′	

*blaTEM*	Forward	5⁣′-TTTCGTGTCGCCCTTATTCC-3⁣′	[36]
	Reverse	5⁣′-ATCGTTGTCAGAAGTAAGTTGG-3⁣′	

*blaKPC*	Forward	5⁣′-ATGTCACTGTATCGCCGTCT-3⁣′	[37]
	Reverse	5⁣′-GCTGTGCTTGTCATCCTTGT-3⁣′	

**Table 2:** Thermal cycler settings used for amplification of antibiotic resistance genes.

**Gene**	**Thermal cycler program for the ARGs**			
	**Steps**	**Temperature (°C)**	**Time**	**No. of cycles**
*blaSHV*	Initial denaturation	94	5 min	1
	Denaturation	94	15 s	30
	Annealing	60	30 s	30
	Extension	72	1 min	30
	Final extension	72	5 min	1

*blaTEM*	Initial denaturation	95	5 min	1
	Denaturation	94	30 s	30
	Annealing	53	45 s	30
	Extension	72	45 s	30
	Final extension	72	10 min	1

*blaKPC*	Initial denaturation	95	5 min	1
	Denaturation	95	1 min	30
	Annealing	53	30 s	30
	Extension	72	90 s	30
	Final extension	72	10 min	1

**Table 3:** Sample accession numbers and their similarities to reference genes.

**Sample ID**	**NCBI GenBank accession numbers for *K. pneumoniae* isolates**	
	**Similarities to reference genes**	**Accession number**
KP15	87.33%	PQ459313
KP21	94.48%	PQ459318
KP22	96.74%	PQ459385
KP27	99.29%	PQ459319
KP30	91.29%	PQ459326

**Table 4:** Prevalence of the *mrkA* gene among the clinical isolates.

***mrkA***	**Biofilm category**	
	**Weak biofilm formers,** **n** **(%)**	**Moderate biofilm formers,** **n** **(%)**
Present (*n* = 15)	5 (33.33)	10 (66.66)
Absent (*n* = 4)	1 (25)	3 (75)

**Table 5:** Biofilm formation categorization across clinical isolates. Strains that formed biofilms were grouped into strong, moderate, and weak biofilm formers. The prevalence of the groups is shown in numbers and percentages.

	**Biofilm producers**			**Nonbiofilm producers**
	**Weak,** **n** **(%)**	**Moderate,** **n** **(%)**	**Strong,** **n** **(%)**	**Nonproducers,** **n** **(%)**
*K. pneumoniae*	12 (23.5)	34 (66.7)	5 (9.8)	0 (0)
Clinical specimen				
UTI (*n* = 26)	5 (19.23)	19 (73.07)	2 (7.69)	0 (0)
Body fluids (*n* = 3)	1 (33.33)	2 (66.66)	0 (0)	0 (0)
Wound and tissue samples (*n* = 9)	5 (55.55)	3 (33.33)	1 (11.11)	0 (0)
Respiratory samples (*n* = 8)	0 (0)	6 (75)	2 (25)	0 (0)
Swabs and cultures (*n* = 5)	1 (20)	4 (80)	0 (0)	0 (0)

**Table 6:** Gene expression quantification for the tested samples. Detectable threshold for expression was set at Ct < 35, and samples above it were labeled with NA to indicate nondetectable expression. Values for ΔCt are presented as the mean ± standard deviation (SD), and the 95% confidence interval is provided for the final fold change values.

**Sample ID**	**Gene expression quantification**					
	**Gene**	Δ**Ctvalue (pH 5)**	Δ**Ctvalue (pH 7)**	ΔΔ**Ct**	**Fold change**	**95% CI**
K29	*mrkA*	−1.18 ± 0.44	0.42 ± 0.47	−1.6	3.0	1.94–4.74
	*blaSHV*	−2.55 ± 0.24	1.41 ± 0.64	−3.96	15.6	9.70–24.97
	*blaTEM*	−2.72 ± 0.53	−0.35 ± 0.39	−2.37	5.2	2.90–9.22

K34	*blaTEM*	12.46 ± 0.27	24.59 ± 0.16 (NA)	—	—	—
	*blaSHV*	−2.86 ± 0.73	23.66 ± 0.48 (NA)	—	—	—

K37	*mrkA*	1.06 ± 0.82	3.43 ± 0.14	−2.37	5.2	3.28–8.15
	*blaSHV*	2.41 ± 0.43	6.02 ± 0.77	−3.61	12.2	6.60–22.59

K47	*blaSHV*	14.56 ± 0.25	18.19 ± 0.33	3.63	12.4	9.31–16.46

**Table 7:** Mean ΔCt values for the groups across the two environmental conditions. It highlights that *mrkA*-positive strains have higher gene expression in both environmental conditions.

	**Mean**Δ**Ctvalue (pH 5)**	**Mean**Δ**Ctvalue (pH 7)**
**Gene**	***mrkA* positive**	
*blaSHV*	−0.07	3.72
	***mrkA* negative**	
*blaSHV*	5.85	20.93

**Table 8:** The *p* value and *W* of the Shapiro–Wilk normality test for the individual columns. *p* values above 0.05 were considered nonnormal, and those with a *p* value below 0.05 were further confirmed for their normality with a Q–Q plot.

**Columns**	**Shapiro–Wilk normality test**	
	**W**	**p** **value**
Age	0.95389	0.4589
OD	0.97035	0.7835
Cefepime MIC	0.82096	0.002342
Ceftriaxone MIC	0.85352	0.007657
Meropenem MIC	0.62489	8.03e − 06
Imipenem MIC	0.65812	1.835e − 05
Amikacin MIC	0.80206	0.001224
Gentamicin MIC	0.76439	0.0003631
Ciprofloxacin MIC	0.80632	0.001414
Levofloxacin MIC	0.66787	2.359e − 05
Colistin MIC	0.50718	5.816e − 07
Gene expression	0.80055	<2.2e − 16

**Table 9:** Kruskal–Wallis's test results for comparing age values to infection type and gender; both are nonsignificant, as highlighted by the *p* value.

**Variables**	**Kruskal–Wallis's rank sum test**		
	**Chi-squared (** **χ** ^2^ **)**	**Degrees of freedom (** **d** **f** **)**	**p** **value**
Age & infection type	23.017	19	0.237
Age & gender	1.027	1	0.311

**Table 10:** Kruskal–Wallis's test results for comparing antibiotics' MIC values across the categorical columns. No significant difference in the MIC values was detected across the groups.

	**Kruskal–Wallis's rank sum test**								
	**CFP**	**CTX**	**MEM**	**IPM**	**AMK**	**GEN**	**CIP**	**LEVO**	**COL**
	**Gender & antibiotics**								
Chi-squared (**χ**^2^)	0.41	1.85	0.68	0.96	0.0	0.83	0.07	0.02	2.8
df	1	1	1	1	1	1	1	1	1
*p* value	0.52	0.17	0.41	0.32	1	0.36	0.78	0.89	0.09
	**Infection type & antibiotics**								
Chi-squared (**χ**^2^)	9.36	10.4	9.28	9.76	6.91	7.87	9.81	7.89	12.9
df	8	8	8	8	8	8	8	8	8
*p* value	0.31	0.23	0.32	0.28	0.54	0.44	0.27	0.44	0.11
	***blaSHV* & antibiotics**								
Chi-squared (**χ**^2^)	0.422	0.019	1.50	1.26	0.0	0.08	3.47	2.73	0.56
df	1	1	1	1	1	1	1	1	1
*p* value	0.516	0.89	0.22	0.26	1.0	0.77	0.063	0.098	0.452
	***blaTEM* & antibiotics**								
Chi-squared (**χ**^2^)	0.76	0.376	0.827	0.127	1.357	3.323	1.097	0.020	2.80
df	1	1	1	1	1	1	1	1	1
*p* value	0.38	0.54	0.363	0.721	0.244	0.068	0.294	0.885	0.094

**Table 11:** One-way ANOVA results for the comparison of OD across multiple groups. None of the *p* values are below the 0.05 threshold, indicating that OD does not differ significantly across the groups.

**O** **D** **& variables**	**One-way ANOVA**				
	**d** **f**	**Sum Sq**	**Mean Sq**	**F** **value**	**Pr**(>**F**)
Gender	1	0.000022	0.0000221	0.015	0.905
Infection type	8	0.01083	0.001354	0.928	0.533
*mrkA* gene	1	0.000337	0.0003372	0.229	0.639
Cefepime	2	0.000967	0.0004837	0.316	0.733
Ceftriaxone	2	0.001715	0.0008575	0.579	0.572
Meropenem	2	0.000006	0.000003	0.002	0.998
Imipenem	1	0.000088	0.000088	0.059	0.811
Amikacin	1	0.000811	0.0008114	0.561	0.464
Gentamicin	1	0.001162	0.001161	0.814	0.38
Ciprofloxacin	1	0.000179	0.0001787	0.12	0.733
Levofloxacin	2	0.004545	0.002272	1.742	0.207

**Table 12:** Results of two-way ANOVA for the effect of *mrkA*, biofilm category, and age on OD. Significant *p* values are indicated by asterisks (⁣^∗∗∗^*p* < 0.001 and *p* < 0.1).

**Interactions**	**Two-way ANOVA with interaction**					
	**d** **f**	**Sum Sq**	**Mean Sq**	**F** **value**	**Pr**(>**F**)	**Significance**
*mrkA*	1	0.000337	0.000337	1.116	0.3115	
Biofilm category	1	0.017906	0.017906	59.286	5.55e − 06	⁣^∗∗∗^
Age	1	0.001188	0.001188	3.935	0.0706	.
*mrkA*: Biofilm category	1	0.001328	0.001328	4.396	0.0579	.
*mrkA*: Age	1	0.001023	0.001023	3.388	0.0905	.
Biofilm category: Age	1	0.000012	0.000012	0.040	0.8454	
Residuals	12	0.003624	0.000302			

**Table 13:** Fisher's exact test for the association between *blaSHV*, *blaTEM*, and *mrkA* genes. It shows the *p* value, odds ratio, and CI for the tested genes.

**Groups**	**Fisher's exact test**		
	**p** **value**	**Odds ratio**	**95% confidence interval**
*mrkA* & *blaSHV*	0.03509	Inf	0.8102
*mrkA* & *blaTEM*	1	0.5174	[0.00819, 8.42104]

**Table 14:** The Mann–Whitney *U* test and the Wilcoxon signed-rank test; for both tests, *W*/*Z*, *p* value, and effect size (*r*) are given. The data overall suggest higher expression among *mrkA*-positive strains and also a higher expression in acidic conditions for both groups.

**Groups**	**Mann–Whitney** **U** **test**		
	**W**/**Z**	**p** **value**	**r**
	**At pH 5**		
*mrkA*+ & *mrkA*−	5	0.5714	−0.2635
	**At pH 7**		
*mrkA*+ & *mrkA*−	0	0.03571	0
	**Wilcoxon's signed-rank test**		
pH 5 & pH 7	−2.0926	0.03639	−0.7398

## Data Availability

The datasets generated during the current study, along with the analysis code, are available in the supplementary information files associated with this article. The raw data are provided in CSV files, each corresponding to the Results section. The code for the data analysis is also provided in both R and Python. Furthermore, the nucleotide sequence data for the isolates sequenced in this study have been deposited in the NCBI GenBank database under the following accession numbers (PQ459313, PQ459318, PQ459385, PQ459319, and PQ459326).

## Supporting Information

Supporting Information Additional supporting information can be found online in the Supporting Information section. The following supporting information is provided to support the findings and reproducibility of this research. (1) README: instructions on how to use the data and run the analysis code. (2) Folder1_Data: contains the raw data in CSV format. (3) Folder2_Scipts: contains the code for the analysis. (4) Folder3_Outputs: figures and plot generated for the study.

## References

[1] Urban-Chmiel R., Marek A., Stępień-Pyśniak D. (2022). Antibiotic Resistance in Bacteria—A Review. *Antibiotics*.

[2] Uddin T. M., Chakraborty A. J., Khusro A. (2021). Antibiotic Resistance in Microbes: History, Mechanisms, Therapeutic Strategies and Future Prospects. *Journal of Infection and Public Health*.

[3] Cosentino F., Viale P., Giannella M. (2023). MDR/XDR/PDR or DTR? Which Definition Best Fits the Resistance Profile of *Pseudomonas aeruginosa*?. *Current Opinion in Infectious Diseases*.

[4] Li Y., Kumar S., Zhang L., Wu H. (2022). *Klebsiella pneumonia* and Its Antibiotic Resistance: A Bibliometric Analysis. *BioMed Research International*.

[5] Li Y., Kumar S., Zhang L., Wu H., Wu H. (2023). Characteristics of Antibiotic Resistance Mechanisms and Genes of *Klebsiella pneumoniae*. *Open Medicine*.

[6] Kot B., Piechota M., Szweda P. (2023). Virulence Analysis and Antibiotic Resistance of *Klebsiella pneumoniae* Isolates From Hospitalised Patients in Poland. *Scientific Reports*.

[7] Byrne M. K., Miellet S., McGlinn A. (2019). The Drivers of Antibiotic Use and Misuse: The Development and Investigation of a Theory Driven Community Measure. *BMC Public Health*.

[8] Sharma S., Mohler J., Mahajan S. D., Schwartz S. A., Bruggemann L., Aalinkeel R. (2023). Microbial Biofilm: A Review on Formation, Infection, Antibiotic Resistance, Control Measures, and Innovative Treatment. *Microorganisms*.

[9] Høiby N., Bjarnsholt T., Givskov M., Molin S., Ciofu O. (2010). Antibiotic Resistance of Bacterial Biofilms. *International Journal of Antimicrobial Agents*.

[10] Zhao A., Sun J., Liu Y. (2023). Understanding Bacterial Biofilms: From Definition to Treatment Strategies. *Frontiers in Cellular and Infection Microbiology*.

[11] Bo L., Sun H., Li Y.-D. (2024). Combating Antimicrobial Resistance: The Silent War. *Frontiers in Pharmacology*.

[12] Vestby L. K., Grønseth T., Simm R., Nesse L. L. (2020). Bacterial Biofilm and Its Role in the Pathogenesis of Disease. *Antibiotics*.

[13] Hussein S., Ahmed S. K., Mohammed S. M. (2025). Recent Developments in Antibiotic Resistance: An Increasing Threat to Public Health–A Review. *Annals of Animal Science*.

[14] Shree P., Singh C. K., Sodhi K. K., Surya J. N., Singh D. K. (2023). Biofilms: Understanding the Structure and Contribution Towards Bacterial Resistance in Antibiotics. *Medicine in Microecology*.

[15] Salmani A., Shakerimoghaddam A., Pirouzi A., Delkhosh Y., Eshraghi M. (2020). Correlation Between Biofilm Formation and Antibiotic Susceptibility Pattern in Acinetobacter baumannii MDR Isolates Retrieved From Burn Patients. *Gene Reports*.

[16] Qi L., Li H., Zhang C. (2016). Relationship Between Antibiotic Resistance, Biofilm Formation, and Biofilm-Specific Resistance in Acinetobacter baumannii. *Frontiers in Microbiology*.

[17] Li L., Gao X., Li M. (2024). Relationship Between Biofilm Formation and Antibiotic Resistance of *Klebsiella pneumoniae* and Updates on Antibiofilm Therapeutic Strategies. *Frontiers in Cellular and Infection Microbiology*.

[18] Pajohesh R., Tajbakhsh E., Momtaz H., Rahimi E. (2022). Relationship Between Biofilm Formation and Antibiotic Resistance and Adherence Genes in Staphylococcus aureus Strains Isolated From Raw Cow Milk in Shahrekord, Iran. *International Journal of Microbiology*.

[19] Alshaikh S. A., El-banna T., Sonbol F., Farghali M. H. (2024). Correlation Between Antimicrobial Resistance, Biofilm Formation, and Virulence Determinants in Uropathogenic *Escherichia coli* From Egyptian Hospital. *Annals of Clinical Microbiology and Antimicrobials*.

[20] Lessa F. C., Sievert D. M. (2023). Antibiotic Resistance: A Global Problem and the Need to Do More. *Clinical Infectious Diseases*.

[21] Caneiras C., Lito L., Melo-Cristino J., Duarte A. (2019). Community- and Hospital-Acquired *Klebsiella pneumoniae* Urinary Tract Infections in Portugal: Virulence and Antibiotic Resistance. *Microorganisms*.

[22] Li Y., Ni M. (2023). Regulation of Biofilm Formation in *Klebsiella pneumoniae*. *Frontiers in Microbiology*.

[23] Sameni F., Hajikhani B., Hashemi A., Owlia P., Niakan M., Dadashi M. (2023). The Relationship Between the Biofilm Genes and Antibiotic Resistance in Stenotrophomonas maltophilia. *International Journal of Microbiology*.

[24] Huang Y. J., Liao H. W., Wu C. C., Peng H. L. (2009). MrkF Is a Component of Type 3 Fimbriae in *Klebsiella pneumoniae*. *Research in Microbiology*.

[25] Alwan A. H., Alwan A. H., Abass S. M. (2017). The Effects of U. V Light on mrkA, mrkD Genes in Local Isolates of *Klebsiella pneumoniae*. *Al-Mustansiriyah Journal of Science*.

[26] Uruén C., Chopo-Escuin G., Tommassen J., Mainar-Jaime R. C., Arenas J. (2021). Biofilms as Promoters of Bacterial Antibiotic Resistance and Tolerance. *Antibiotics*.

[27] Lin X. C., Li C. L., Zhang S. Y., Yang X. F., Jiang M. (2024). The Global and Regional Prevalence of Hospital-Acquired Carbapenem-Resistant *Klebsiella pneumoniae* Infection: A Systematic Review and Meta-Analysis. *Open Forum Infectious Diseases*.

[28] Hou Q., Bai X., Li W. (2018). Design of Primers for Evaluation of Lactic Acid Bacteria Populations in Complex Biological Samples. *Frontiers in Microbiology*.

[29] Okonechnikov K., Golosova O., Fursov M. (2012). Unipro UGENE: A Unified Bioinformatics Toolkit. *Bioinformatics*.

[30] Mahmood K. I., Najmuldeen H. H. R., Rachid S. K. (2022). Physiological Regulation for Enhancing Biosynthesis of Biofilm-Inhibiting Secondary Metabolites in Streptomyces Cellulosae. *Cellular and Molecular Biology*.

[31] Clinical and Laboratory Standards Institute (CLSI) (2023). *Performance Standards for Antimicrobial Susceptibility Testing*.

[32] Mori S., Yamada A., Kawai K. (2024). Evaluation of the Biofilm Detection Capacity of the Congo Red Agar Method for Bovine Mastitis-Causing Bacteria. *Japanese Journal of Veterinary Research*.

[33] Sabença C., Costa E., Sousa S. (2023). Evaluation of the Ability to Form Biofilms in KPC-Producing and ESBL-Producing *Klebsiella pneumoniae* Isolated From Clinical Samples. *Antibiotics*.

[34] Raheema R. H., Hadi S. H., Chabuck Z. A. G. (2024). Study of Some Virulence Genes From Uropathogenic *Klebsiella pneumoniae* and *Pseudomonas aeruginosa* Isolated in Wasit Province, Iraq. *Medical Journal of Babylon*.

[35] Babini G. S., Livermore D. M. (2000). Are SHV *β*-Lactamases Universal in*Klebsiella pneumoniae*?. *Antimicrobial Agents and Chemotherapy*.

[36] Bali E. B., Açık L., Sultan N. (2010). Phenotypic and Molecular Characterization of SHV, TEM, CTX-M and Extended-Spectrum-Lactamase Produced by *Escherichia coli*, *Acinobacter baumannii* and Klebsiella Isolates in a Turkish Hospital. *African Journal of Microbiology Research*.

[37] Ghasemnejad A., Doudi M., Amirmozafari N. (2019). The Role of the bla (KPC) Gene in Antimicrobial Resistance of *Klebsiella pneumoniae*. *Iranian Journal of Microbiology*.

[38] Ponchel F., Toomes C., Bransfield K. (2003). Real-Time PCR Based on SYBR-Green I Fluorescence: An Alternative to the TaqMan Assay for a Relative Quantification of Gene Rearrangements, Gene Amplifications and Micro Gene Deletions. *BMC Biotechnology*.

[39] Minh B. Q., Schmidt H. A., Chernomor O. (2020). IQ-TREE 2: New Models and Efficient Methods for Phylogenetic Inference in the Genomic Era. *Molecular Biology and Evolution*.

[40] Letunic I., Bork P. (2024). Interactive Tree of Life (iTOL) v6: Recent Updates to the Phylogenetic Tree Display and Annotation Tool. *Nucleic Acids Research*.

[41] Patil I. (2021). Visualizations With Statistical Details: The “ggstatsplot” Approach. *Journal of Open Source Software*.

[42] Zhongzheng M., Chen J.-C., Ma Z.-Z. (2024). A Nanocarrier-Mediated dsRNA Oral Delivery Enhances RNAi Efficiency in Thrips. *Entomologia Generalis*.

[43] Müller-Schulte E., Tuo M. N., Akoua-Koffi C., Schaumburg F., Becker S. L. (2020). High Prevalence of ESBL-Producing *Klebsiella pneumoniae* in Clinical Samples From Central Côte D’ivoire. *International Journal of Infectious Diseases*.

[44] Sharma J., Sharma D., Singh A., Sunita K. (2022). Colistin Resistance and Management of Drug Resistant Infections. *Canadian Journal of Infectious Diseases and Medical Microbiology*.

[45] Theodorakis N., Feretzakis G., Hitas C. (2024). Immunosenescence: How Aging Increases Susceptibility to Bacterial Infections and Virulence Factors. *Microorganisms*.

[46] Theodorakis N., Feretzakis G., Hitas C. (2024). Antibiotic Resistance in the Elderly: Mechanisms, Risk Factors, and Solutions. *Microorganisms*.

[47] Alves J., Prendki V., Chedid M. (2024). Challenges of Antimicrobial Stewardship Among Older Adults. *European Journal of Internal Medicine*.

[48] Guerra M. E. S., Destro G., Vieira B. (2022). *Klebsiella pneumoniae* Biofilms and Their Role in Disease Pathogenesis. *Frontiers in Cellular and Infection Microbiology*.

[49] Hammouda Z. K., Wasfi R., Abdeltawab N. F. (2023). Hormonal Drugs: Influence on Growth, Biofilm Formation, and Adherence of Selected Gut Microbiota. *Frontiers in Cellular and Infection Microbiology*.

[50] Aleman F. D. D., Valenzano D. R. (2019). Microbiome Evolution During Host Aging. *PLoS Pathogens*.

[51] Bakhtiari R., Javadi A., Aminzadeh M., Molaee-Aghaee E., Shaffaghat Z. (2021). Association Between Presence of RmpA, MrkA and MrkD Genes and Antibiotic Resistance in Clinical *Klebsiella pneumoniae* Isolates From Hospitals in Tehran, Iran. *Iranian Journal of Public Health*.

[52] Gual-de-Torrella A., Delgado-Valverde M., Pérez-Palacios P. (2022). Prevalence of the Fimbrial Operon mrkABCD, mrkA Expression, Biofilm Formation and Effect of Biocides on Biofilm Formation in Carbapenemase-Producing *Klebsiella pneumoniae* Isolates Belonging or Not Belonging to High-Risk Clones. *International Journal of Antimicrobial Agents*.

[53] Langstraat J., Bohse M., Clegg S. (2001). Type 3 Fimbrial Shaft (MrkA) of *Klebsiella pneumoniae*, but Not the Fimbrial Adhesin (MrkD), Facilitates Biofilm Formation. *Infection and Immunity*.

[54] Wilksch J. J., Yang J., Clements A. (2011). MrkH, a Novel c-di-GMP-Dependent Transcriptional Activator, Controls *Klebsiella pneumoniae* Biofilm Formation by Regulating Type 3 Fimbriae Expression. *PLoS Pathogens*.

[55] Ashwath P., Deekshit V. K., Rohit A. (2022). Biofilm Formation and Associated Gene Expression in Multidrug-Resistant *Klebsiella pneumoniae* Isolated From Clinical Specimens. *Current Microbiology*.

[56] Ojdana D., Sacha P., Wieczorek P. (2014). The Occurrence of *bla*_CTX-M_, *bla*_SHV_, and *bla*_TEM_ Genes in Extended-Spectrum *β*-Lactamase-Positive Strains of *Klebsiella pneumoniae*, *Escherichia coli*, and *Proteus mirabilis* in Poland. *International Journal of Antibiotics*.

[57] Babani S., Raoof W., Rachid S. (2022). A Systematic Review: The Current Status of Carbapenem Resistance in Iraq. *World Bulletin of Public Health*.

[58] Hamad S. T., Ghaim K. K. (2022). Prevalence of Carbapenemase Genes in *Klebsiella pneumoniae* Isolates From Patients With Urinary Tract Infections in Baghdad Hospitals. *Iraqi Journal of Biotechnology*.

[59] Firoozeh F., Aghaseyed-Hosseini M., Zibaei M., Piroozmand A. (2016). Detection of blaKPC and blaGES Carbapenemase Genes in *Klebsiella pneumoniae* Isolated From Hospitalized Patients in Kashan, Iran. *Recent Patents on Anti-Infective Drug Discovery*.

[60] Metwally L., Gomaa N., Attallah M., Kamel N. (2013). High Prevalence of *Klebsiella pneumoniae* Carbapenemase-Mediated Resistance in K. pneumoniae Isolates From Egypt. *Eastern Mediterranean Health Journal*.

[61] Ma L., Qu Y., Wang W., Wang D. (2023). Characterization of *Klebsiella pneumoniae* Carrying the *bla*_NDM-1_ Gene in IncX3 Plasmids and the Rare In1765 in an IncFIB-IncHI1B Plasmid. *Frontiers in Cellular and Infection Microbiology*.

[62] Ugbo E. N., Anyamene C. O., Moses I. B. (2020). Prevalence of blaTEM, blaSHV, and blaCTX-M Genes Among Extended Spectrum Beta-Lactamase-Producing *Escherichia coli* and *Klebsiella pneumoniae* of Clinical Origin. *Gene Reports*.

[63] Dumaru R., Baral R., Shrestha L. B. (2019). Study of Biofilm Formation and Antibiotic Resistance Pattern of Gram-Negative Bacilli Among the Clinical Isolates at BPKIHS, Dharan. *BMC Research Notes*.

[64] Sheikh S. W., Ali A., Ahsan A., Shakoor S., Shang F., Xue T. (2021). Insights Into Emergence of Antibiotic Resistance in Acid-Adapted Enterohaemorrhagic *Escherichia coli*. *Antibiotics*.

[65] Rice L. B. (2012). Mechanisms of Resistance and Clinical Relevance of Resistance to *β*-Lactams, Glycopeptides, and Fluoroquinolones. *Mayo Clinic Proceedings*.

[66] Herrera-Espejo S., Domínguez-Miranda J. L., Rodríguez-Mogollo J. I., Pachón J., Cordero E., Pachón-Ibáñez M. E. (2024). Effects of pH on the Pathogenicity of *Escherichia coli* and *Klebsiella pneumoniae* on the Kidney: In Vitro and In Vivo Studies. *International Journal of Molecular Sciences*.

[67] Zarzecka U., Chajęcka-Wierzchowska W., Zakrzewski A., Zadernowska A., Fraqueza M. J. (2022). High Pressure Processing, Acidic and Osmotic Stress Increased Resistance to Aminoglycosides and Tetracyclines and the Frequency of Gene Transfer Among Strains From Commercial Starter and Protective Cultures. *Food Microbiology*.

