# Supplementary material for: Exploring the Relationship Between Biofilm Formation and Antibiotic Resistance Genes in Clinically Isolated Klebsiella pneumoniae
Source: Int J Microbiol. 2025 Oct 16;2025:3833882. doi: 10.1155/ijm/3833882 (PMC12549196; doi:10.1155/ijm/3833882)
Supplement: Supporting Information — Additional supporting information can be found online in the Supporting Information section. The following supporting information is provided to support the findings and reproducibility of this research. (1) README: instructions on how to use the data and run the analysis code. (2) Folder1_Data: contains the raw data in CSV format. (3) Folder2_Scipts: contains the code for the analysis. (4) Folder3_Outputs: figures and plot generated for the study. [file 3833882.f1.zip › Data-analysis/README.pdf]

Exploring the Relationship Between Biofilm Formation and Antibiotic Resistance Genes in Clinically Isolated *Klebsiella pneumonia* (Data analysis)

Corresponding Author: Hevar N. Abdulqadir
Email: hivar.neyaz@chu.edu.iq
Date: 11/4/2025

1. Project Overview

This repository contains the data, and the scripts used in the analysis of the manuscripts titled '*Exploring the Relationship Between Biofilm Formation and Antibiotic Resistance Genes in Clinically Isolated Klebsiella pneumonia*'.

The study investigated the relationship between biofilm formation and antibiotic resistance genes in *Klebsiella pneumonia* using genotypic and phenotypic methods. All exploratory data analysis and statistical tests were performed using R and Python.

2. Folder and File Structure

- **Folder1_Data**
  - ***Samples.csv***

Column name	Description	Data Type
Sample code	Unique id for each strain	Character
Age	Age of the patients	Integer
Gender	Gender of the patients	Character
Sample area	Sampling location	Character

  - ***antibiotic.csv***

Column name	Description	Data Type
Sample code	Unique id for each strain	Character
Age	Age of the patients	Integer
Gender	Gender of the patients	Character
Sample area	Sampling location	Character
Antibiotics columns	Contain MIC and resistance category for each antibiotic	Character, MIC values (Integer)

- **biofilm_data.csv**

Column name	Description	Data Type
Sample code	Unique id for each strain	Character
Age	Age of the patients	Integer
Gender	Gender of the patients	Character
Sample area	Sampling location	Character
MrkA	Presence of mrkA gene	Character (positive/negative)
OD	Crystal violet assay OD	Integer
Biofilm category	Weak, moderate, strong, non-biofilm formers. Was based on OD	Character
Antibiotics columns	Contain MIC and resistance category for each antibiotic	Character,<br>MIC values (Integer)

- **Ct_values.cv**

Column name	Description	Data Type
Sample ID	Unique ID for the tested strains	Integer
Group	Either mrkA positive or negative	Character
Gene	*blaTEM, blaSHV, mrkA*	Character
Rep 1 pH5	Normalized Ct values at pH5 (Replicate 1)	Integer
Rep 2 pH5	Normalized Ct values at pH5 (Replicate 2)	Integer
Rep 3 pH5	Normalized Ct values at pH5 (Replicate 3)	Integer
Ct values ph5	Averaged normalized Ct values at pH5	Integer
SD pH5	Standard deviation for the Ct values at pH5	Integer
Rep 1 pH7	Normalized Ct values at pH7 (Replicate 1)	Integer

Rep 2 pH7	Normalized Ct values at pH7 (Replicate 2)	Integer
Rep 3 pH7	Normalized Ct values at pH7 (Replicate 3)	Integer
Ct values ph7	Averaged Normalized Ct values at pH7	Integer
SD pH7	Standard deviation for the Ct values at pH7	Integer
Delta-Delta Ct value	Difference between pH5 and pH7 gene expression	Integer
Fold change	Ratio of expression level across the pH levels	Integer
Combined SD	Combined SD data for both pH	Integer
Lower bound	Lower bound for 95% confidence interval	Integer
Higher bound	Higher bound for 95% confidence interval	Integer

- **Gene_f_data.csv**

Column name	Description	Data Type
Sample ID	Unique ID and condition	Character
Gene	*blaTEM, blaSHV, mrkA*	Character
Ct value	Normalized Ct value	Integer
Rest of the columns	Fluorescent reading from Roche device	Integer

- **antibiotic_resistance_gene.csv**

Column name	Description	Data Type
Sample code	Unique id for each strain	Character
Age	Age of the patients	Integer

Gender	Gender of the patients	Character
Sample area	Sampling location	Character
Antibiotics columns	Contain MIC and resistance category for each antibiotic	Character, MIC values (Integer)
SHV	Presence of SHV (Positive/negative)	Character
TEM	Presence of TEM (Positive/negative)	Character

- **all_variables.csv**

Column name	Description	Data Type
Sample code	Unique id for each strain	Character
Age	Age of the patients	Integer
Gender	Gender of the patients	Character
Sample area	Sampling location	Character
MrkA	Presence of mrkA gene	Character (positive/negative)
SHV	Presence of blaSHV	Character
TEM	Presence of blaTEM	Character
KPC	Presence of blaKPC	Character
OD	Crystal violet assay OD	Integer
Biofilm category	Weak, moderate, strong, non-biofilm formers. Was based on OD	Character
Antibiotics columns	Contain MIC and resistance category for each antibiotic	Character, MIC values (Integer)

- **crystal_violet_assay.csv**

Column name	Description	Data Type
Sample Code	Unique ID and condition	Character
Replicate 1	Replicate 1 OD value	Integer
Replicate 2	Replicate 2 OD value	Integer
Replicate 3	Replicate 3 OD value	Integer
Average OD	Average of all three replicate	Integer
SD	Standard deviations for the replicate	Integer

ODc (Control)	Average of three negative wells	Integer
Mean±SD	Error	Integer
Biofilm category	Weak, moderate, strong, non-biofilm formers. Was based on OD	Character

- **all_samples.csv**

Column name	Description	Data Type
Sample code	Unique id for each strain	Character
Age	Age of the patients	Integer
Gender	Gender of the patients	Character
Sample area	Sampling location	Character
Average OD	Crystal violet assay OD, average of three replicates	Integer
Biofilm category	Weak, moderate, strong, non-biofilm formers. Was based on OD	Character

- **Gene_normalization.csv**

Column name	Description	Data Type
Sample ID	Unique ID for the tested strains	Integer
Group	Either mrkA positive or negative	Character
Genes	*blaTEM, blaSHV, mrkA*	Character
Rep 1 T pH5	Replicate one for target gene at pH5	Integer
Rep 2 T pH5	Replicate two for target gene at pH5	Integer
Rep 3 T pH5	Replicate three for target gene at pH5	Integer
Ct values ph5	Averaged Raw Ct values at pH5 (target)	Integer

Rep 1 I pH5	Replicate one for Internal gene at pH5	Integer
Rep 2 I pH5	Replicate two for Internal gene at pH5	Integer
Rep 3 I pH5	Replicate three for Internal gene at pH5	Integer
16S Ct value at Ph5	Averaged internal control Ct value at pH5	Integer
ΔCt Value pH5	Normalized Ct values at pH5	Integer
Rep 1 T pH7	Replicate one for target gene at pH7	Integer
Rep 2 T pH7	Replicate two for target gene at pH7	Integer
Rep 3 T pH7	Replicate three for target gene at pH7	Integer
Ct values ph7	Averaged Raw Ct values at pH7 (target)	Integer
Rep 1 I pH7	Replicate one for Internal gene at pH7	Integer
Rep 2 I pH7	Replicate two for Internal gene at pH7	Integer
Rep 3 I pH7	Replicate three for Internal gene at pH7	Integer
16S Ct value at Ph7	Averaged internal control Ct value at pH7	Integer
ΔCt Value pH7	Normalized Ct values at pH7	Integer
ΔΔCt Value	Difference between pH5 and pH7 gene expression	Integer
Fold change	Ratio of expression level across the pH levels	Integer

- **Species_Selection_Criteria.csv**

Column name	Description	Data Type
Gender	Gender of the patient	Character
Age	Age of the patient	Integer
Bacterial name	Isolated bacterial species from infection site	Character
Antibiotics	Tested antibiotics on the isolated sample	Character
Sensitivity	Automated system results for that antibiotic	Character

Note: The file named **Species_Selection_Criteria.csv** was used solely as an initial check to estimate the prevalence of each bacterial species in Suleimani city. Based on that preliminary check *klebsiella pneumonia* was chosen to be studied. The file was not used for further analysis.

- **Folder2_Scripts**
    - **R_data_analysis.R**
      This file contains the R scripts that were used in the analysis of this study.
    - **Python_data_analysis.ipynb**
      This file contains the code for the python analysis.
    - **R_markdown.pdf**
      The file contains the codes from the file named **R_data_analysis.R** with their corresponding outputs.
    - **Data_analysis _python.pdf**
      This file contains the code from the file named **Python_data_analysis.ipynb** with their corresponding outputs.

- **Folder3_Outputs**
  This folder contains the outputs of the R and python code. The files also contain other figures and outputs that were used in the study for instance, it contains the raw gel images.

3. Software and Dependencies

This analysis used the following environments:

- For R:

- R version 4.4.2 (2024-10-31)
- Key packages used in the analysis.

tidyr, dplyr, ggplot2, fmsb, ggridges, pheatmap, emmeans, car, effectsize, cluster, factoextra, coin, gt, tidyverse, ggstatsplot, palmerpenguins, gridExtra

-For Python

- Python version 3.12 (2023-10-02)
- Key packages used in the analysis
  Pandas, Numpy, Seaborn, Matplotlib

4. How to reproduce the analysis

To reproduce the results presented in the manuscript please follow the steps below:

1- Please note that you must download both python and R in order to fully reproduce the analysis.
2- After downloading python and R, install the required packages (Mentioned above)
3- Download all the files provided in this repository and save them to a single folder.
4- Open the file named data_analysis_script.R (for the analysis in R), and data_analysis.ipynb (for the analysis in python)
5- Set your working directory to the folder you created. Further notes are given in the script's files.
6- Run the analysis (select all and press **shift** + **enter**)

Note: If you prefer not to run the analysis yourself, the files named R_markdown.pdf and data_analysis _python.pdf contains the code and the corresponding output.

5. Data Access

All data either raw or processed are publicly available alongside the published article. If you choose to use the data or the code, please cite this paper accordingly.

6. Contacts

If you have any inquiries, please contact

Name: Hevar N. Abdulqadir

Affiliation: Charmo university

Email: hivar.neyaz@chu.edu.iq
