# Supplementary material for: Exploring the Relationship Between Biofilm Formation and Antibiotic Resistance Genes in Clinically Isolated Klebsiella pneumoniae
Source: Int J Microbiol. 2025 Oct 16;2025:3833882. doi: 10.1155/ijm/3833882 (PMC12549196; doi:10.1155/ijm/3833882)
Supplement: Supporting Information — Additional supporting information can be found online in the Supporting Information section. The following supporting information is provided to support the findings and reproducibility of this research. (1) README: instructions on how to use the data and run the analysis code. (2) Folder1_Data: contains the raw data in CSV format. (3) Folder2_Scipts: contains the code for the analysis. (4) Folder3_Outputs: figures and plot generated for the study. [file 3833882.f1.zip › Data-analysis/Folder2_Scripts/data_analysis _python.pdf]

In [93]:
```
import pandas as pd
import numpy as np
import matplotlib.pyplot as plt
import seaborn as sns
```

In [94]:
```
df = pd.read_csv('/Users/hevarneaz/Downloads/MS DATA/publication ma
```

In [95]:
```
df.head(5)
```

Out[95]:

	sample code	Age	Gender	Sample area	MrkA	SHV	TEM	KPC	
0	S12	49.0	Male	Bon and tissue	Negative	Negative	Positive	Negative	0.[illegible]
1	S13	31.0	Male	Pouch	Positive	Positive	Negative	Negative	0.
2	S24	0.3	Male	Urine	Positive	Positive	Positive	Negative	0.[illegible]
3	S27	11.0	Female	Urine	Positive	Positive	Negative	Negative	0.[illegible]
4	S28	74.0	Female	Wound	Positive	Positive	Negative	Negative	0.[illegible]

5 rows × 28 columns

In [96]:
```
df.drop('sample code', axis=1, inplace=True)
```

In [97]:
```
df.info()
```

```
<class 'pandas.core.frame.DataFrame'>
RangeIndex: 19 entries, 0 to 18
Data columns (total 27 columns):
 #   Column              Non-Null Count  Dtype
---  ------              --------------  -----
 0   Age                 19 non-null     float64
 1   Gender              19 non-null     object
 2   Sample area         19 non-null     object
 3   MrkA                19 non-null     object
 4   SHV                 19 non-null     object
 5   TEM                 19 non-null     object
 6   KPC                 19 non-null     object
 7   OD                  19 non-null     float64
 8   Biofilm category    19 non-null     object
 9   Cefepime (MIC)      19 non-null     int64
 10  Cefepime            19 non-null     object
 11  Ceftriaxone (MIC)   19 non-null     int64
 12  Ceftriaxone         19 non-null     object
 13  Meropenem (MIC)     19 non-null     float64
 14  Meropenem           19 non-null     object
 15  Imipenem (MIC)      19 non-null     float64
 16  Imipenem            19 non-null     object
 17  Amikacin (MIC)      19 non-null     int64
 18  Amikacin            19 non-null     object
 19  Gentamicin (MIC)    19 non-null     int64
 20  Gentamicin          19 non-null     object
 21  Ciprofloxacin (MIC) 19 non-null     float64
 22  Ciprofloxacin       19 non-null     object
 23  Levofloxacin (MIC)  19 non-null     int64
 24  Levofloxacin        19 non-null     object
 25  Colistin (MIC)      19 non-null     float64
 26  Colistin            19 non-null     object
dtypes: float64(6), int64(5), object(16)
memory usage: 4.1+ KB
```

In [98]:
```
df.describe()
```

Out[98]:

	Age	OD	Cefepime (MIC)	Ceftriaxone (MIC)	Meropenem (MIC)	Imipener (MIC
**count**	19.000000	19.000000	19.000000	19.000000	19.000000	19.00000
**mean**	43.642105	0.145842	14.105263	29.000000	3.973684	4.40789
**std**	25.822972	0.037578	10.224669	21.221059	5.508765	5.36527
**min**	0.300000	0.087000	1.000000	1.000000	0.500000	0.25000
**25%**	25.500000	0.117500	3.000000	16.000000	0.750000	1.00000
**50%**	46.000000	0.148000	16.000000	32.000000	1.000000	4.00000
**75%**	60.000000	0.167000	16.000000	32.000000	4.000000	4.00000
**max**	82.000000	0.214000	32.000000	64.000000	16.000000	16.00000

In [99]:
```
sns.pairplot(df, hue = "Age")
```

Out[99]: <seaborn.axisgrid.PairGrid at 0x30ca7dee0>

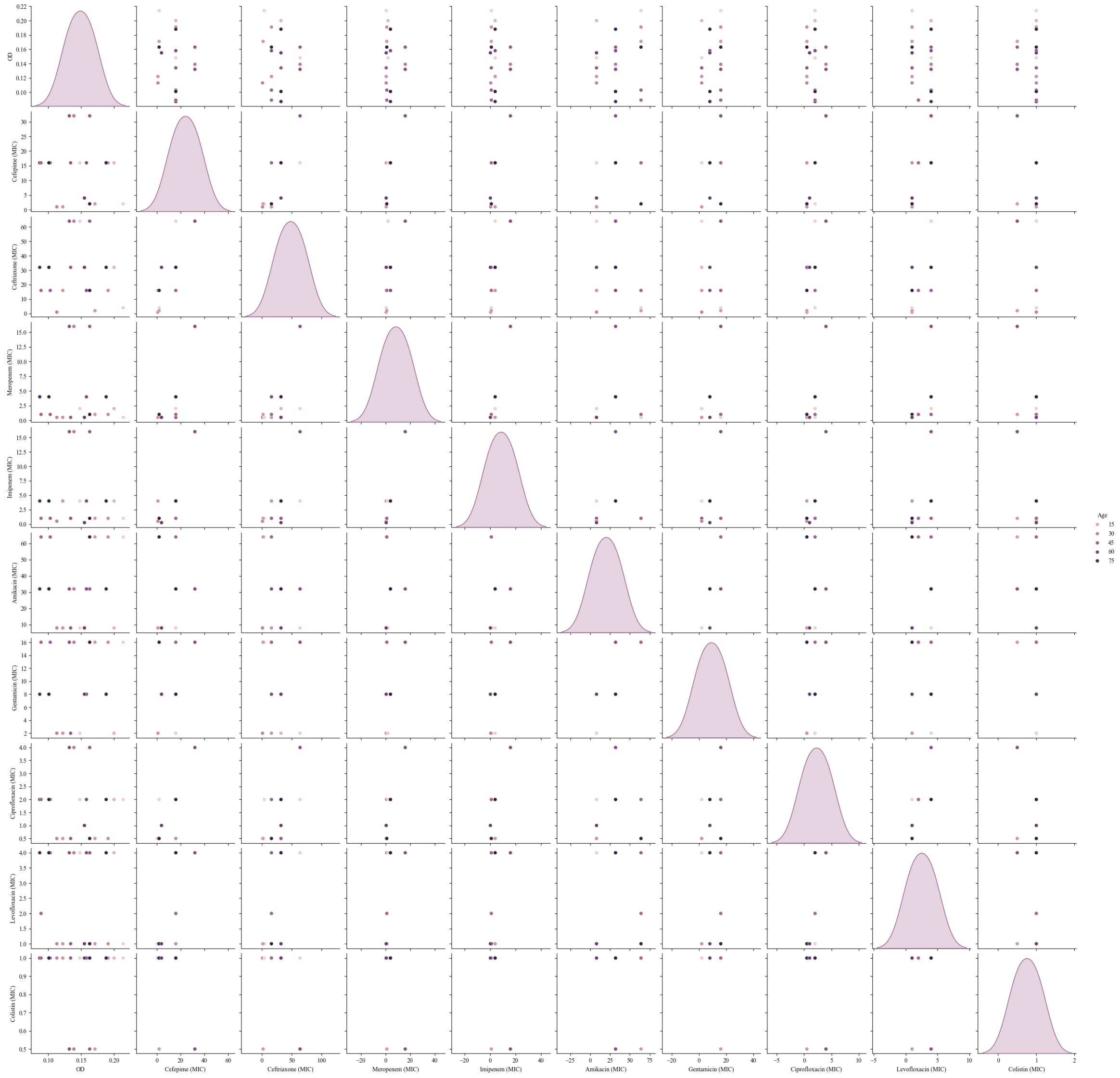

In [100…

```
df_static = pd.DataFrame(df.describe())
```

In [101…

```
df_static
```

Out[101…

	Age	OD	Cefepime (MIC)	Ceftriaxone (MIC)	Meropenem (MIC)	Imipenei (MIC
**count**	19.000000	19.000000	19.000000	19.000000	19.000000	19.00000
**mean**	43.642105	0.145842	14.105263	29.000000	3.973684	4.40789
**std**	25.822972	0.037578	10.224669	21.221059	5.508765	5.36527
**min**	0.300000	0.087000	1.000000	1.000000	0.500000	0.25000
**25%**	25.500000	0.117500	3.000000	16.000000	0.750000	1.00000
**50%**	46.000000	0.148000	16.000000	32.000000	1.000000	4.00000
**75%**	60.000000	0.167000	16.000000	32.000000	4.000000	4.00000
**max**	82.000000	0.214000	32.000000	64.000000	16.000000	16.00000

In [102…

```
sns.countplot(data=df, x = 'Biofilm category', palette='magma',orde

plt.title('Distribution of Biofilm Categories', fontsize=16, fontwe
plt.xlabel('Biofilm Category', fontsize=12)
plt.ylabel('Count', fontsize=12)

for i in plt.gca().containers:
    plt.bar_label(i, label_type='edge', fontsize=10, padding=2)

sns.despine()

plt.tight_layout()
```

```
/var/folders/rb/3xv7x0z91nq4nl41p9dwhfdh0000gn/T/ipykernel_76062/407
4522397.py:1: FutureWarning:

Passing `palette` without assigning `hue` is deprecated and will be
removed in v0.14.0. Assign the `x` variable to `hue` and set `legend
=False` for the same effect.

  sns.countplot(data=df, x = 'Biofilm category', palette='magma',ord
er= df['Biofilm category'].value_counts().index)
```

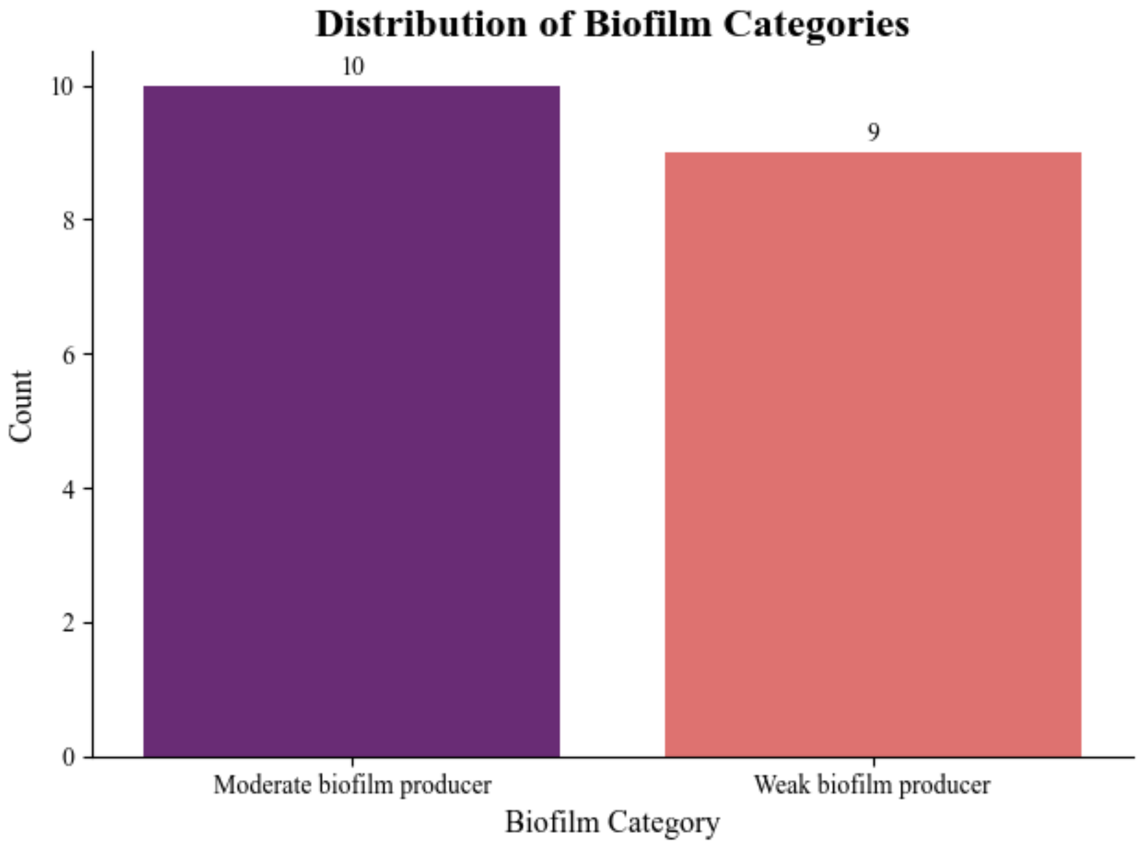

In [103…

```
sns.boxenplot(data = df, x ='Biofilm category', y = 'OD', hue = 'Bi
plt.legend(loc= 'upper left', bbox_to_anchor=(1, 1))
sns.despine(right = True, top = True,)
```

```
No artists with labels found to put in legend.  Note that artists wh
ose label start with an underscore are ignored when legend() is call
ed with no argument.
```

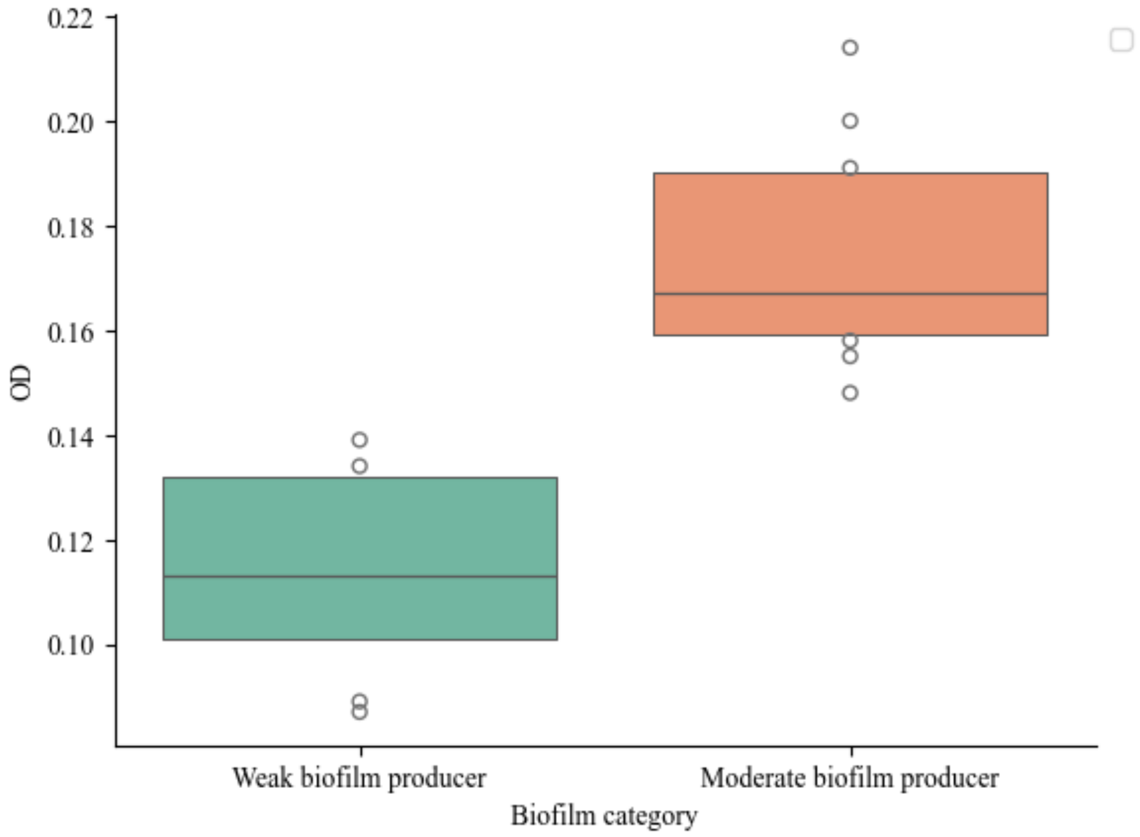

In [104…
```
sns.violinplot(data = df, x ="Biofilm category", y ="OD", hue = "Bi
plt.title("Distrubition of OD Accros Biofilm Category")
plt.legend(loc= 'upper left', bbox_to_anchor=(1, 1))
```

```
No artists with labels found to put in legend.  Note that artists wh
ose label start with an underscore are ignored when legend() is call
ed with no argument.
```

Out[104…
```
<matplotlib.legend.Legend at 0x30ecf1a30>
```

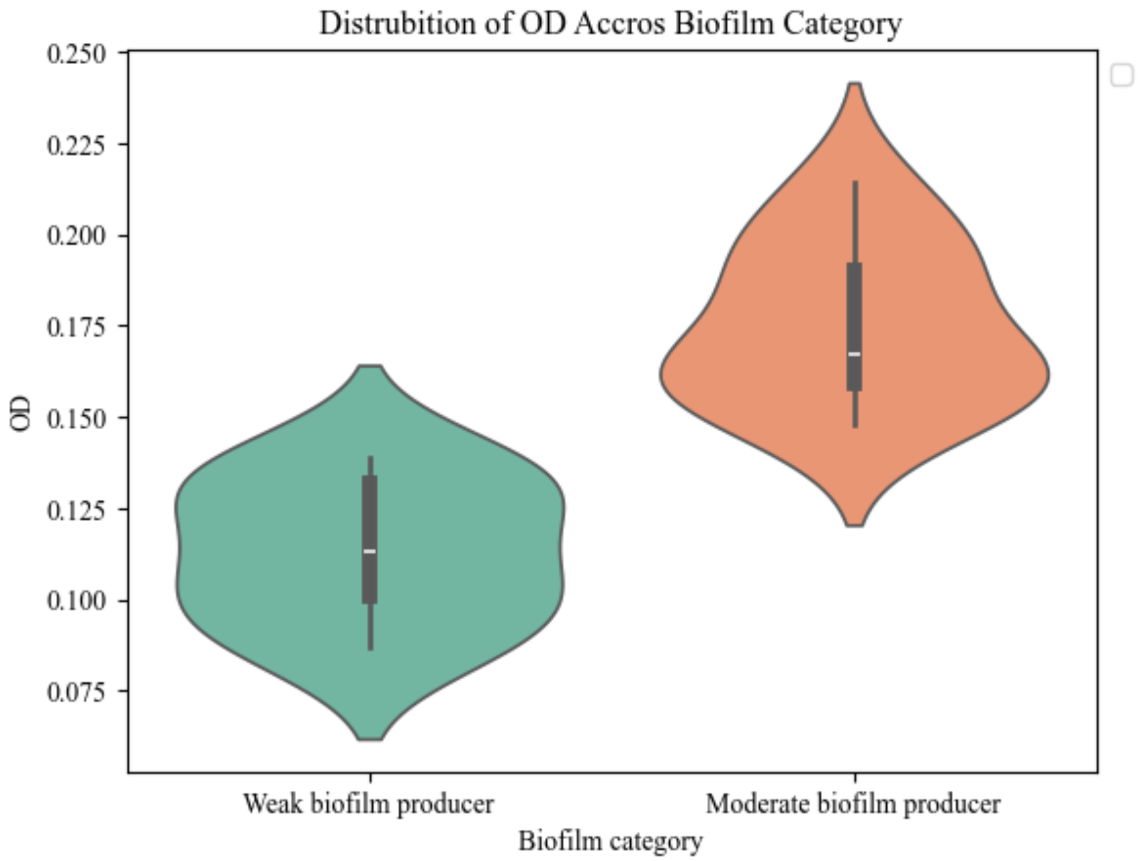

In [105…
```
df2 = pd.read_csv('all_50.csv')
age_bins = [0, 30, 50, 70, float('inf')]
age_labels = ['Youth', 'Middle-aged', 'Senior', 'Elderly']

df2['Age Group'] = pd.cut(df2['Age '], bins=age_bins, labels=age_la

print(df2[['Age ', 'Age Group']].head())

print(df2['Age Group'].value_counts())
age_group = df2.groupby('Age Group')
print(age_group)
```

```
   Age     Age Group
0  20.0        Youth
1  70.0      Elderly
2  23.0        Youth
3  65.0       Senior
4  43.0  Middle-aged
Age Group
Middle-aged    17
Youth          15
Elderly        10
Senior          9
Name: count, dtype: int64
<pandas.core.groupby.generic.DataFrameGroupBy object at 0x3059e41a0>
```

```
/var/folders/rb/3xv7x0z91nq4nl41p9dwhfdh0000gn/T/ipykernel_76062/378
358921.py:10: FutureWarning: The default of observed=False is deprec
ated and will be changed to True in a future version of pandas. Pass
observed=False to retain current behavior or observed=True to adopt
the future default and silence this warning.
  age_group = df2.groupby('Age Group')
```

In [106…

```
plt.figure(figsize=(10, 6))
sns.violinplot(data = df2, x ="Biofilm Category", y = "Average OD",
plt.tight_layout()
plt.rcParams['font.family'] = 'Times New Roman'
plt.ylabel('Average OD', fontsize=20, fontweight = 'bold',)
plt.xlabel('Biofilm Category', fontsize=20,fontweight = 'bold')
plt.yticks(fontsize=15)
plt.xticks(fontsize=15)
sns.despine(right = True, top = True)
```

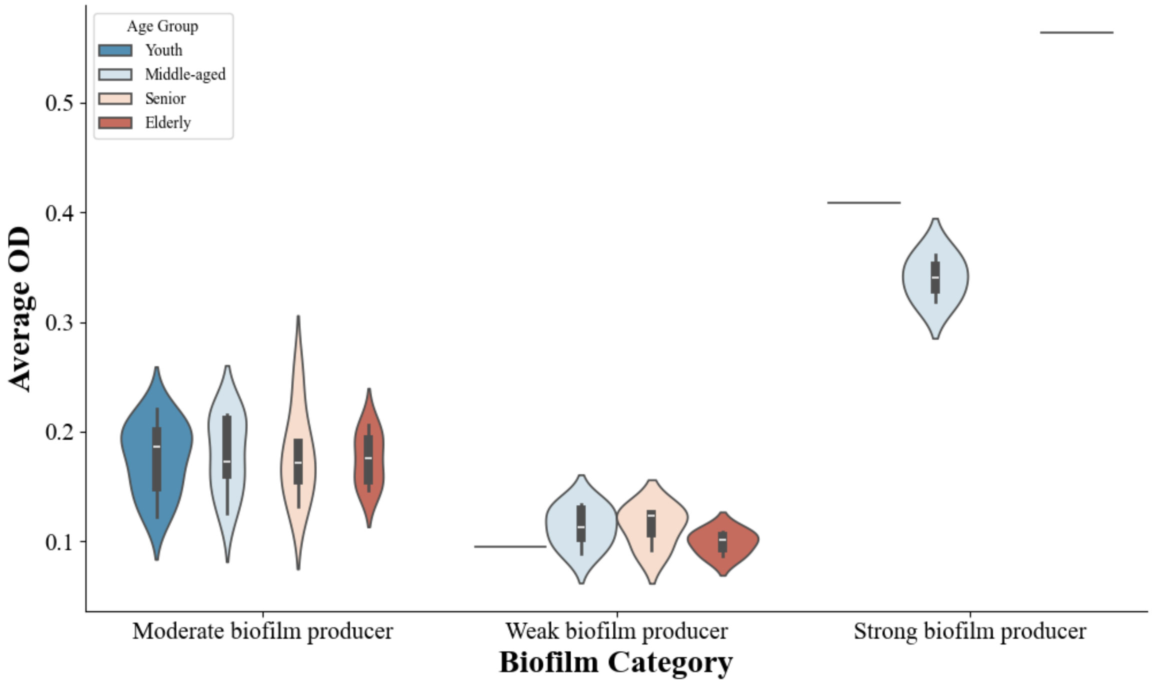

In [107…

```
plt.figure(figsize=(18, 6))
sns.countplot(data = df, x ='Sample area')
```

Out[107… <Axes: xlabel='Sample area', ylabel='count'>

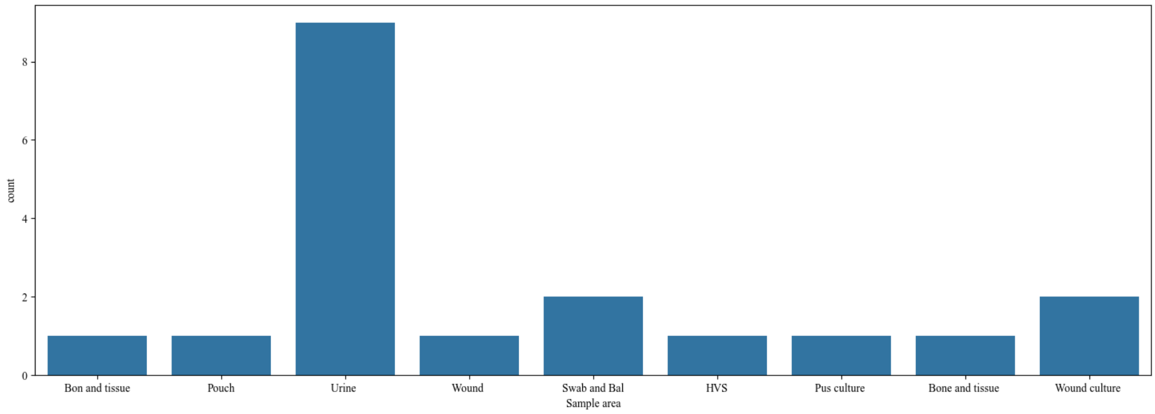

In [108…

```
col = df[['Cefepime (MIC)', 'Ceftriaxone (MIC)', 'Meropenem (MIC)',
       'Amikacin (MIC)','Gentamicin (MIC)','Ciprofloxacin (MIC)',
       'Levofloxacin (MIC)', 'Colistin (MIC)']]
col
```

Out[108…

	Cefepime (MIC)	Ceftriaxone (MIC)	Meropenem (MIC)	Imipenem (MIC)	Amikacin (MIC)	Gentamicin (MIC)	C
**0**	16	32	0.5	1.00	8	2	
**1**	1	1	0.5	0.50	8	2	
**2**	2	4	0.5	1.00	64	16	
**3**	16	32	2.0	4.00	8	2	
**4**	16	32	4.0	4.00	32	8	
**5**	32	64	16.0	16.00	32	16	
**6**	32	64	16.0	16.00	32	16	
**7**	32	64	16.0	16.00	32	16	
**8**	2	2	1.0	1.00	64	16	
**9**	16	16	4.0	4.00	32	8	
**10**	1	16	0.5	4.00	8	2	
**11**	16	16	1.0	1.00	64	16	
**12**	16	16	1.0	1.00	64	16	
**13**	2	16	1.0	1.00	64	16	
**14**	16	16	1.0	1.00	64	16	
**15**	16	32	4.0	4.00	32	8	
**16**	16	64	2.0	4.00	8	2	
**17**	16	32	4.0	4.00	32	8	
**18**	4	32	0.5	0.25	8	8	

In [109…

```
corr_df = col.corr()
corr_df
```

Out[109…

	Cefepime (MIC)	Ceftriaxone (MIC)	Meropenem (MIC)	Imipenem (MIC)	Amikacin (MIC)	Ge
**Cefepime (MIC)**	1.000000	0.811397	0.843367	0.824535	-0.058023	
**Ceftriaxone (MIC)**	0.811397	1.000000	0.775104	0.804983	-0.387883	
**Meropenem (MIC)**	0.843367	0.775104	1.000000	0.984155	-0.069761	
**Imipenem (MIC)**	0.824535	0.804983	0.984155	1.000000	-0.157740	
**Amikacin (MIC)**	-0.058023	-0.387883	-0.069761	-0.157740	1.000000	
**Gentamicin (MIC)**	0.275336	0.003450	0.378997	0.282961	0.857499	
**Ciprofloxacin (MIC)**	0.852948	0.753205	0.883927	0.872761	-0.003842	
**Levofloxacin (MIC)**	0.735095	0.637655	0.570798	0.575971	-0.152372	-
**Colistin (MIC)**	-0.539373	-0.487520	-0.797091	-0.775471	-0.126551	-

In [110…

```
plt.figure(figsize=(10,10))
sns.heatmap(data = corr_df,annot=True, cmap='RdBu_r',)
```

Out[110…

```
<Axes: >
```

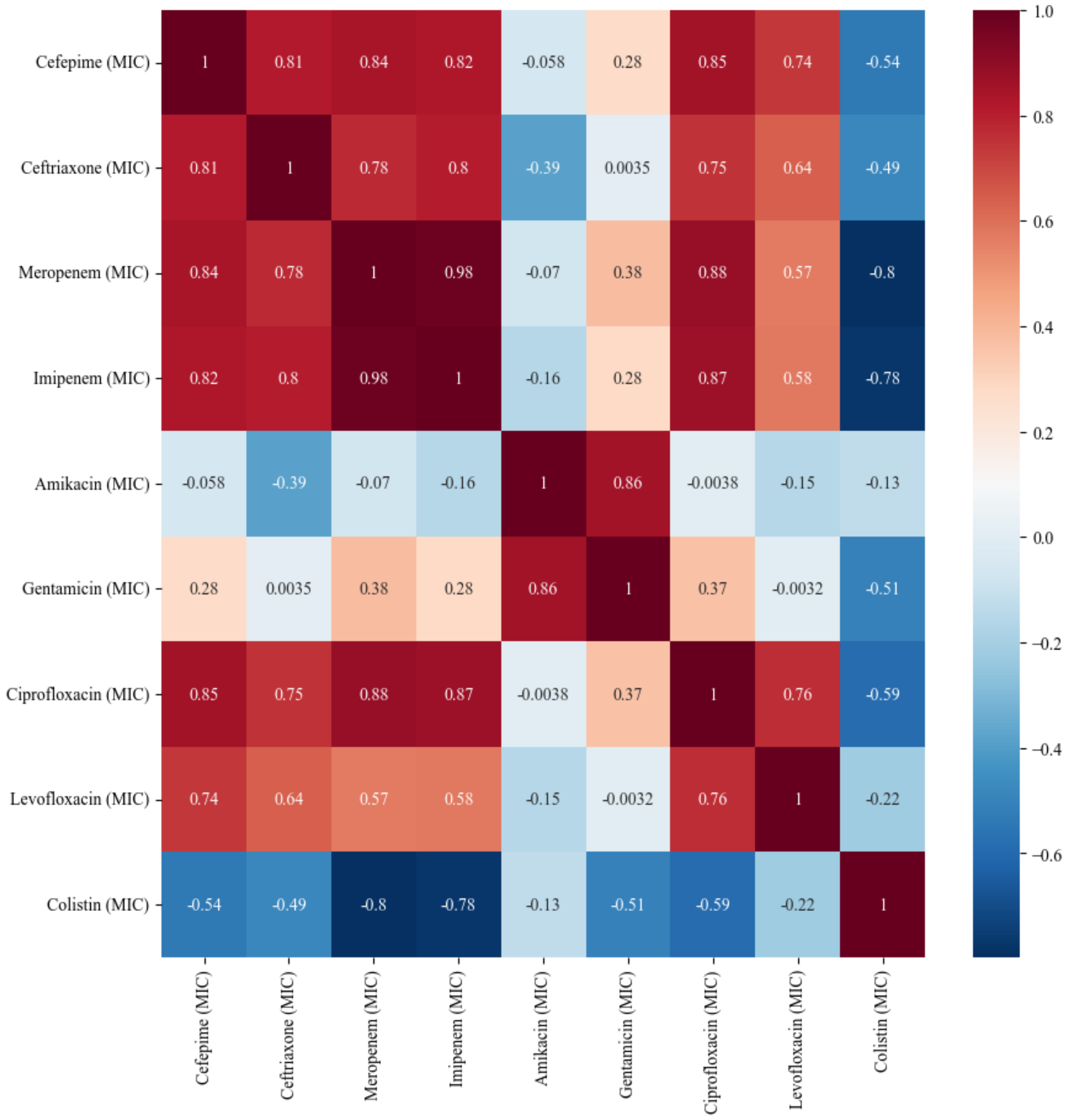

In [111…

```
corr_df= df.select_dtypes(include=['float64','int64'])
corr_df_matrix=corr_df.corr()
```

In [112…

```
corr_df_matrix
```

Out [112…

	Age	OD	Cefepime (MIC)	Ceftriaxone (MIC)	Meropenem (MIC)	In…
**Age**	1.000000	-0.365998	0.152321	0.083436	0.153471	[illegible]
**OD**	-0.365998	1.000000	-0.124736	-0.062978	-0.040008	[illegible]
**Cefepime (MIC)**	0.152321	-0.124736	1.000000	0.811397	0.843367	[illegible]
**Ceftriaxone (MIC)**	0.083436	-0.062978	0.811397	1.000000	0.775104	[illegible]
**Meropenem (MIC)**	0.153471	-0.040008	0.843367	0.775104	1.000000	[illegible]
**Imipenem (MIC)**	0.059897	-0.025909	0.824535	0.804983	0.984155	[illegible]
**Amikacin (MIC)**	0.059737	0.120060	-0.058023	-0.387883	-0.069761	[illegible]
**Gentamicin (MIC)**	0.116978	0.119988	0.275336	0.003450	0.378997	[illegible]
**Ciprofloxacin (MIC)**	0.060489	-0.055266	0.852948	0.753205	0.883927	[illegible]
**Levofloxacin (MIC)**	0.217927	-0.172751	0.735095	0.637655	0.570798	[illegible]
**Colistin (MIC)**	0.079966	-0.076351	-0.539373	-0.487520	-0.797091	[illegible]

In [113…

```
plt.figure(figsize=(10,8))
sns.heatmap(data = corr_df_matrix, annot=True, cmap="magma",linewid
            , annot_kws={"size": 10})
plt.xticks(rotation=90, ha='right', fontsize=8)
plt.yticks(rotation=0, fontsize=8)
plt.yticks(rotation=0)
plt.tight_layout()
plt.show()
```

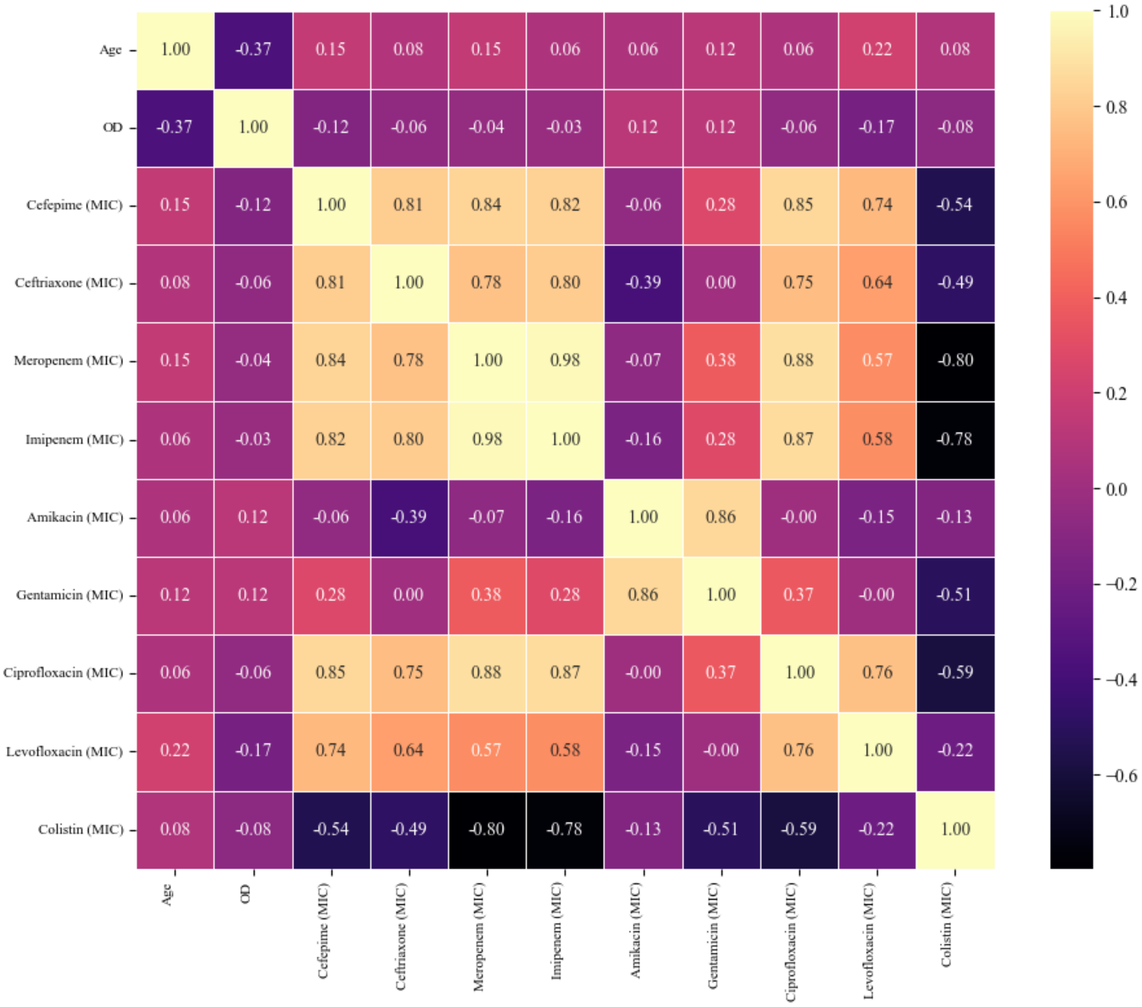
Age
OD
Cefepime (MIC)
Ceftriaxone (MIC)
Meropenem (MIC)
Imipenem (MIC)
Amikacin (MIC)
Gentamicin (MIC)
Ciprofloxacin (MIC)
Levofloxacin (MIC)
Colistin (MIC)
1.00 -0.37 0.15 0.08 0.15 0.06 0.06 0.12 0.06 0.22 0.08
-0.37 1.00 -0.12 -0.06 -0.04 -0.03 0.12 0.12 -0.06 -0.17 -0.08
0.15 -0.12 1.00 0.81 0.84 0.82 -0.06 0.28 0.85 0.74 -0.54
0.08 -0.06 0.81 1.00 0.78 0.80 -0.39 0.00 0.75 0.64 -0.49
0.15 -0.04 0.84 0.78 1.00 0.98 -0.07 0.38 0.88 0.57 -0.80
0.06 -0.03 0.82 0.80 0.98 1.00 -0.16 0.28 0.87 0.58 -0.78
0.06 0.12 -0.06 -0.39 -0.07 -0.16 1.00 0.86 -0.00 -0.15 -0.13
0.12 0.12 0.28 0.00 0.38 0.28 0.86 1.00 0.37 -0.00 -0.51
0.06 -0.06 0.85 0.75 0.88 0.87 -0.00 0.37 1.00 0.76 -0.59
0.22 -0.17 0.74 0.64 0.57 0.58 -0.15 -0.00 0.76 1.00 -0.22
0.08 -0.08 -0.54 -0.49 -0.80 -0.78 -0.13 -0.51 -0.59 -0.22 1.00
1.0
0.8
0.6
0.4
0.2
0.0
−0.2
−0.4
−0.6
