# Supplementary material for: Exploring the Relationship Between Biofilm Formation and Antibiotic Resistance Genes in Clinically Isolated Klebsiella pneumoniae
Source: Int J Microbiol. 2025 Oct 16;2025:3833882. doi: 10.1155/ijm/3833882 (PMC12549196; doi:10.1155/ijm/3833882)
Supplement: Supporting Information — Additional supporting information can be found online in the Supporting Information section. The following supporting information is provided to support the findings and reproducibility of this research. (1) README: instructions on how to use the data and run the analysis code. (2) Folder1_Data: contains the raw data in CSV format. (3) Folder2_Scipts: contains the code for the analysis. (4) Folder3_Outputs: figures and plot generated for the study. [file 3833882.f1.zip › Data-analysis/Folder2_Scripts/R_markdown.pdf]

Exploring the Relationship Between Biofilm Formation and Antibiotic Resistance Genes in Clinically Isolated Klebsiella pneumonia (Data analysis in R)

Hevar

2025-04-08

Load Required Libraries

These libraries are used throughout the analysis for data manipulation, visualization, and statistical analysis.

```
library(tidyr); library(dplyr); library(ggplot2); library(fmsb); library(ggridges)
library(pheatmap); library(emmeans); library(car); library(effectsize); library(cluster)
library(factoextra); library(coin); library(ggpubr); library(gt);library(tidyverse)
library(ggstatsplot); library(palmerpenguins); library(gridExtra)
```

```
knitr::opts_chunk$set(fig.height = 6, fig.width = 12)
```

Load and Explore Dataset

```
df <- read.csv("all_variables.csv")
head(df,3)  # Preview the first 10 rows
```

```
##   sample.code  Age Gender    Sample.area      MrkA      SHV       TEM      KPC
## 1         S12 49.0   Male  Bon and tissue Negative  Negative Positive  Negative
## 2         S13 31.0   Male           Pouch Positive  Positive Negative  Negative
## 3         S24  0.3   Male          Urine  Positive  Positive Positive  Negative
##      OD          Biofilm.category Cefepime..MIC.   Cefepime Ceftriaxone..MIC.
## 1 0.134     Weak biofilm producer             16 Resistance                32
## 2 0.113     Weak biofilm producer              1  Sensitive                 1
## 3 0.214 Moderate biofilm producer              2  Sensitive                 4
##   Ceftriaxone Meropenem..MIC. Meropenem Imipenem..MIC.  Imipenem Amikacin..MIC.
## 1  Resistance             0.5 Sensitive            1.0 Sensitive              8
## 2   Sensitive             0.5 Sensitive            0.5 Sensitive              8
## 3  Resistance             0.5 Sensitive            1.0 Sensitive             64
##     Amikacin Gentamicin..MIC. Gentamicin Ciprofloxacin..MIC. Ciprofloxacin
## 1  Sensitive                2  Sensitive                 0.5     Sensitive
## 2  Sensitive                2  Sensitive                 0.5     Sensitive
## 3 Resistance               16 Resistance                 2.0    Resistance
##   Levofloxacin..MIC. Levofloxacin Colistin..MIC.  Colistin
## 1                  1    Sensitive              1 Sensitive
## 2                  1    Sensitive              1 Sensitive
## 3                  1    Sensitive              1 Sensitive
```

```r
str(df)       # Structure of the data
```

```
## 'data.frame':    19 obs. of  28 variables:
##  $ sample.code        : chr  "S12" "S13" "S24" "S27" ...
##  $ Age                : num  49 31 0.3 11 74 34 49 54 22 56 ...
##  $ Gender             : chr  "Male " "Male " "Male " "Female " ...
##  $ Sample.area        : chr  "Bon and tissue" "Pouch" "Urine " "Urine " ...
##  $ MrkA               : chr  "Negative " "Positive " "Positive " "Positive " ...
##  $ SHV                : chr  "Negative" "Positive" "Positive" "Positive" ...
##  $ TEM                : chr  "Positive " "Negative " "Positive " "Negative " ...
##  $ KPC                : chr  "Negative" "Negative" "Negative" "Negative" ...
##  $ OD                 : num  0.134 0.113 0.214 0.2 0.087 0.139 0.163 0.132 0.171 0.158 ...
##  $ Biofilm.category   : chr  "Weak biofilm producer" "Weak biofilm producer" "Moderate biofilm produc
##  $ Cefepime..MIC.     : int  16 1 2 16 16 32 32 32 2 16 ...
##  $ Cefepime           : chr  "Resistance" "Sensitive" "Sensitive" "Resistance" ...
##  $ Ceftriaxone..MIC.  : int  32 1 4 32 32 64 64 64 2 16 ...
##  $ Ceftriaxone        : chr  "Resistance" "Sensitive" "Resistance" "Resistance" ...
##  $ Meropenem..MIC.    : num  0.5 0.5 0.5 2 4 16 16 16 1 4 ...
##  $ Meropenem          : chr  "Sensitive" "Sensitive" "Sensitive" "Resistance" ...
##  $ Imipenem..MIC.     : num  1 0.5 1 4 4 16 16 16 1 4 ...
##  $ Imipenem           : chr  "Sensitive" "Sensitive" "Sensitive" "Resistance" ...
##  $ Amikacin..MIC.     : int  8 8 64 8 32 32 32 32 64 32 ...
##  $ Amikacin           : chr  "Sensitive" "Sensitive" "Resistance" "Sensitive" ...
##  $ Gentamicin..MIC.   : int  2 2 16 2 8 16 16 16 16 8 ...
##  $ Gentamicin         : chr  "Sensitive" "Sensitive" "Resistance" "Sensitive" ...
##  $ Ciprofloxacin..MIC.: num  0.5 0.5 2 2 2 4 4 4 0.5 2 ...
##  $ Ciprofloxacin      : chr  "Sensitive" "Sensitive" "Resistance" "Resistance" ...
##  $ Levofloxacin..MIC. : int  1 1 1 4 4 4 4 4 1 4 ...
##  $ Levofloxacin       : chr  "Sensitive" "Sensitive" "Sensitive" "Resistance" ...
##  $ Colistin..MIC.     : num  1 1 1 1 1 0.5 0.5 0.5 0.5 1 ...
##  $ Colistin           : chr  "Sensitive" "Sensitive" "Sensitive" "Sensitive" ...
```

```r
summary(df)   # Summary statistics
```

```
##  sample.code             Age           Gender           Sample.area       
##  Length:19          Min.   : 0.30   Length:19          Length:19         
##  Class :character   1st Qu.:25.50   Class :character   Class :character  
##  Mode  :character   Median :46.00   Mode  :character   Mode  :character  
##                     Mean   :43.64                                        
##                     3rd Qu.:60.00                                        
##                     Max.   :82.00                                        
##      MrkA               SHV                TEM                KPC           
##  Length:19          Length:19          Length:19          Length:19         
##  Class :character   Class :character   Class :character   Class :character  
##  Mode  :character   Mode  :character   Mode  :character   Mode  :character  
##                                                                             
##                                                                             
##                                                                             
##        OD         Biofilm.category   Cefepime..MIC.    Cefepime        
##  Min.   :0.0870   Length:19          Min.   : 1.00   Length:19         
##  1st Qu.:0.1175   Class :character   1st Qu.: 3.00   Class :character  
##  Median :0.1480   Mode  :character   Median :16.00   Mode  :character  
```

```
##  Mean   :0.1458                          Mean   :14.11
##  3rd Qu.:0.1670                          3rd Qu.:16.00
##  Max.   :0.2140                          Max.   :32.00
##  Ceftriaxone..MIC. Ceftriaxone        Meropenem..MIC.   Meropenem
##  Min.   : 1        Length:19          Min.   : 0.500   Length:19
##  1st Qu.:16        Class :character   1st Qu.: 0.750   Class :character
##  Median :32        Mode  :character   Median : 1.000   Mode  :character
##  Mean   :29                           Mean   : 3.974
##  3rd Qu.:32                           3rd Qu.: 4.000
##  Max.   :64                           Max.   :16.000
##  Imipenem..MIC.     Imipenem         Amikacin..MIC.    Amikacin
##  Min.   : 0.250   Length:19          Min.   : 8.00   Length:19
##  1st Qu.: 1.000   Class :character   1st Qu.: 8.00   Class :character
##  Median : 4.000   Mode  :character   Median :32.00   Mode  :character
##  Mean   : 4.408                      Mean   :34.53
##  3rd Qu.: 4.000                      3rd Qu.:64.00
##  Max.   :16.000                      Max.   :64.00
##  Gentamicin..MIC.  Gentamicin        Ciprofloxacin..MIC. Ciprofloxacin
##  Min.   : 2.00    Length:19          Min.   :0.500       Length:19
##  1st Qu.: 5.00    Class :character   1st Qu.:0.500       Class :character
##  Median : 8.00    Mode  :character   Median :2.000       Mode  :character
##  Mean   :10.21                       Mean   :1.789
##  3rd Qu.:16.00                       3rd Qu.:2.000
##  Max.   :16.00                       Max.   :4.000
##  Levofloxacin..MIC. Levofloxacin       Colistin..MIC.     Colistin
##  Min.   :1.000      Length:19          Min.   :0.5000   Length:19
##  1st Qu.:1.000      Class :character   1st Qu.:1.0000   Class :character
##  Median :4.000      Mode  :character   Median :1.0000   Mode  :character
##  Mean   :2.632                         Mean   :0.8947
##  3rd Qu.:4.000                         3rd Qu.:1.0000
##  Max.   :4.000                         Max.   :1.0000
```

Factor Conversion

To Ensure Biofilm.category is treated as a categorical variable.

```
df$Biofilm.category <- factor(df$Biofilm.category)
```

Biofilm Category vs OD Visualization

Scatter and boxplot visualization of OD values by Biofilm category and MrkA marker.

```
h <- ggplot(data = df, aes(x = Biofilm.category, y = OD, color = MrkA))
h + geom_jitter() + geom_boxplot(size =1, alpha = 0.5) +
  scale_color_brewer(palette = 'Set1')
```

Antibiotics	means
Cefepime MIC	14.105263
Ceftriaxone MIC	29.000000
Meropenem MIC	3.973684
Imipenem MIC	4.407895
Amikacin MIC	34.526316

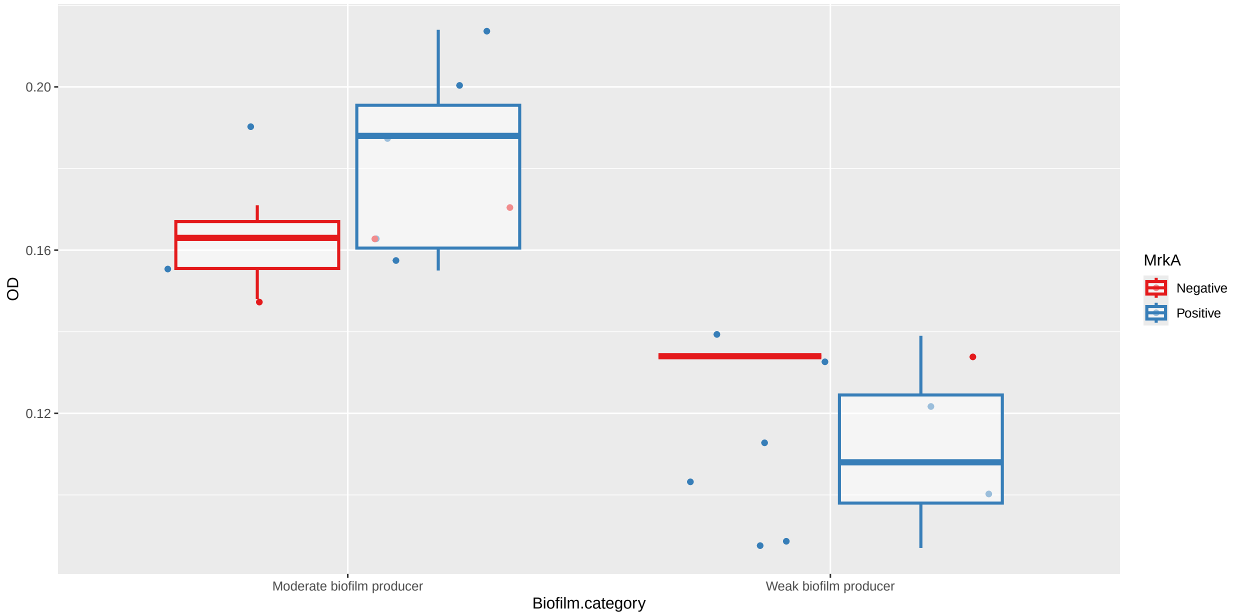

Age Categorization

Categorize age into custom groups.

```
df$Age.cat <- cut(df$Age, breaks = c(0,12,18, 35,50,85)
                  , labels = c('Child', "Teenager", "Adults",'Middle aged', 'senior'),
                  right = F)
```

Bar Plot: Mean MIC Values per Antibiotic

Compute and visualize mean MIC values across antibiotics.

```
Antibiotics <- c("Cefepime MIC", "Ceftriaxone MIC", "Meropenem MIC",
                 "Imipenem MIC", "Amikacin MIC", "Gentamicin MIC","Ciprofloxacin MIC",
                 "Levofloxacin MIC ", "Colistin MIC")
means <- colMeans(df[, c('Cefepime..MIC.', 'Ceftriaxone..MIC.', 'Meropenem..MIC.',
                         'Imipenem..MIC.', 'Amikacin..MIC.', 'Gentamicin..MIC.',
                         'Ciprofloxacin..MIC.', 'Levofloxacin..MIC.', 'Colistin..MIC.')])
mic_mean <- data.frame(Antibiotics,means)
head(mic_mean, 5) %>% gt()
```

Age	OD	Biofilm_category	Antibiotic	MIC
49	0.134	Weak biofilm producer	Cefepime	16.0
49	0.134	Weak biofilm producer	Ceftriaxone	32.0
49	0.134	Weak biofilm producer	Meropenem	0.5
49	0.134	Weak biofilm producer	Imipenem	1.0
49	0.134	Weak biofilm producer	Amikacin	8.0

MIC vs OD Correlation Across Antibiotics

Transform MIC data to long format and visualize correlation with OD.

```
numeric_df <- df %>%
  select(where(is.numeric))

colnames(numeric_df) <- c('Age','OD' ,'Cefepime' ,'Ceftriaxone' ,'Meropenem' ,
                          'Imipenem' ,'Amikacin' ,'Gentamicin' ,'Ciprofloxacin',
                          'Levofloxacin' ,'Colistin')

numeric_df$Biofilm_category <- df$Biofilm.category


mic_values_long <- numeric_df %>%
  pivot_longer(
    cols = starts_with("Cefepime") | starts_with("Ceftriaxone") | starts_with("Meropenem") |
      starts_with("Imipenem") | starts_with("Amikacin") | starts_with("Gentamicin") |
      starts_with("Ciprofloxacin") | starts_with("Levofloxacin") | starts_with("Colistin"),
    names_to = "Antibiotic",
    values_to = "MIC"
  )
head(mic_values_long,5) %>% gt()
```

```
ggplot(data = mic_values_long, aes(x = OD,y = MIC, color = Biofilm_category)) + geom_point(size = 1.5)
  facet_grid(Antibiotic~., scales = 'free') +
  geom_smooth(method = "lm", se = F, linetype = "dashed", color = "black") +
  ggtitle('MIC Relation To OD') +
  labs(color = 'Biofilm Category') + theme_test()
```

```
## `geom_smooth()` using formula = 'y ~ x'
```

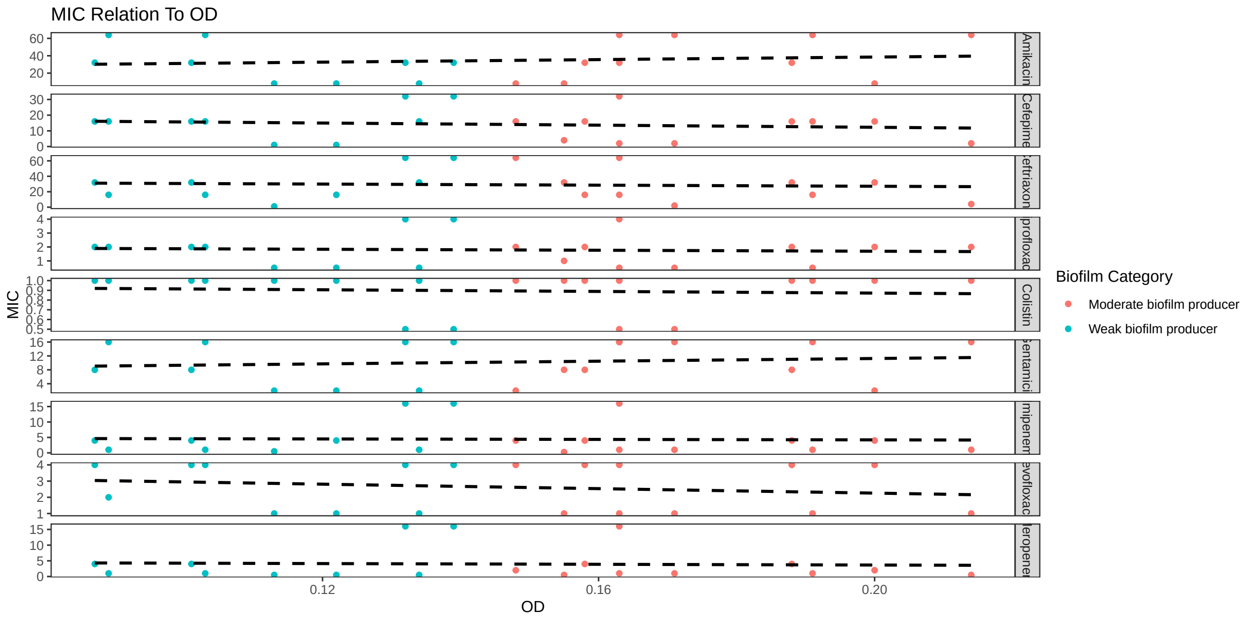

Stacked Barplot of Resistance Categories by Antibiotic

This chunk reshapes the main data and visualizes resistance patterns by antibiotic.

```r
df2 <- df[, c("sample.code", "Age" ,"Gender", "Sample.area", "MrkA", "TEM", "KPC","SHV", "OD","Biofilm.
              "Amikacin" , "Gentamicin", "Ciprofloxacin", "Levofloxacin" , "Colistin")]



df2.long <- df2 %>%
  pivot_longer(
    cols = starts_with("Cefepime") | starts_with("Ceftriaxone") | starts_with("Meropenem") |
    starts_with("Imipenem") | starts_with("Amikacin") | starts_with("Gentamicin") |
    starts_with("Ciprofloxacin") | starts_with("Levofloxacin") | starts_with("Colistin"),
    names_to = "Antibiotics",
    values_to = "degree"
  )
head(df2.long, 5)
```

```
## # A tibble: 5 x 12
##   sample.code   Age Gender  Sample.area    MrkA        TEM     KPC   SHV      OD
##   <chr>       <dbl> <chr>   <chr>          <chr>       <chr>   <chr> <chr> <dbl>
## 1 S12            49 "Male " Bon and tissue "Negative " "Posit~ Nega~ Nega~ 0.134
## 2 S12            49 "Male " Bon and tissue "Negative " "Posit~ Nega~ Nega~ 0.134
## 3 S12            49 "Male " Bon and tissue "Negative " "Posit~ Nega~ Nega~ 0.134
## 4 S12            49 "Male " Bon and tissue "Negative " "Posit~ Nega~ Nega~ 0.134
## 5 S12            49 "Male " Bon and tissue "Negative " "Posit~ Nega~ Nega~ 0.134
## # i 3 more variables: Biofilm.category <fct>, Antibiotics <chr>, degree <chr>
```

```r
first_plot <- ggplot(data = df2.long, aes(x = Antibiotics, fill = degree)) +
  geom_bar(position = 'stack', color = 'black') +
        ggtitle('Resistance levels across antibiotics') + theme_classic() +
        scale_fill_brewer(palette = 'Set2') +
```

```r
  labs(x = 'Antibiotics', y = 'Number of Cases', fill = 'Resistance level')

second_plot <- ggplot(mic_mean, aes(x = reorder(Antibiotics,  -means), y = means, fill = Antibiotics)) +
        geom_bar(stat = "identity", color = "black") +
        scale_fill_brewer(palette = 'Paired') +
        labs(title = "Mean MIC Values Across Antibiotics",x = "Antibiotics", y = "Mean MIC") +
        theme_classic() +
        theme(axis.text.x = element_text(angle = 45, hjust = 1, size = 20))

grid.arrange(first_plot,second_plot, nrow =1,ncol = 2)
```

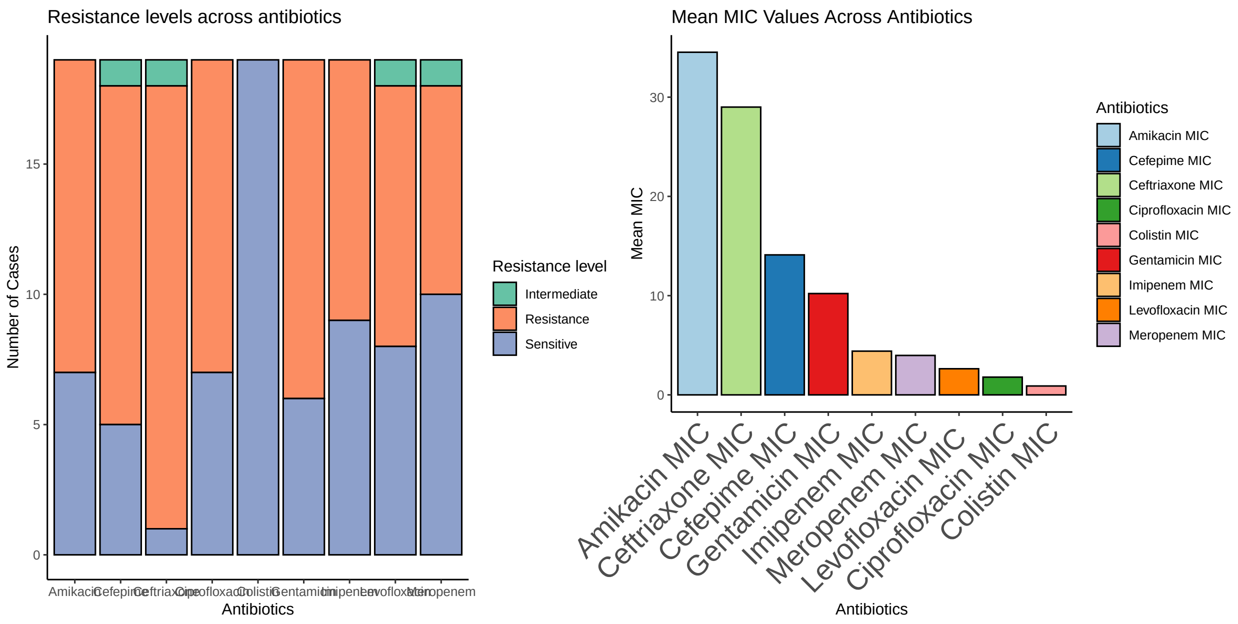

Assigning and Inspecting Data

Assign the MIC values from 'mic__values__long' to the 'df2.long' dataset

```r
df2.long$MIC <- mic_values_long$MIC
```

Reshaping Data (pivot__longer)

Reshaping the df2.long dataset from wide format to long format Pivot columns that start with 'MrkA', 'TEM', 'KPC', or 'SHV' into two columns: 'Genes' and 'gene presence'

```r
df3.long <- df2.long %>%
  pivot_longer(
    cols = starts_with('MrkA') | starts_with('TEM') | starts_with('KPC') |
      starts_with("SHV"),
    names_to = 'Genes',           # Name the column containing the gene names
```

```
    values_to = 'gene presence'  # Name the column containing the presence/absence of the genes
  )
```

Density Plot (MIC and Biofilm Category)

Create a density plot to visualize the distribution of MIC values across different biofilm categories and antibiotics

```
ggplot(data = df3.long , aes(x = MIC, fill = Biofilm.category)) +
  geom_density(alpha = 0.6) +                          # Add density curves with transparency
  facet_grid(Antibiotics~., scales = 'free') +         # Facet the plot by Antibiotic, with independent 
  labs(fill = "Biofilm Category",
       title = 'Density Distribution of MIC Values Across Antibiotics and Biofilm Categories',
       y = 'Density')
```

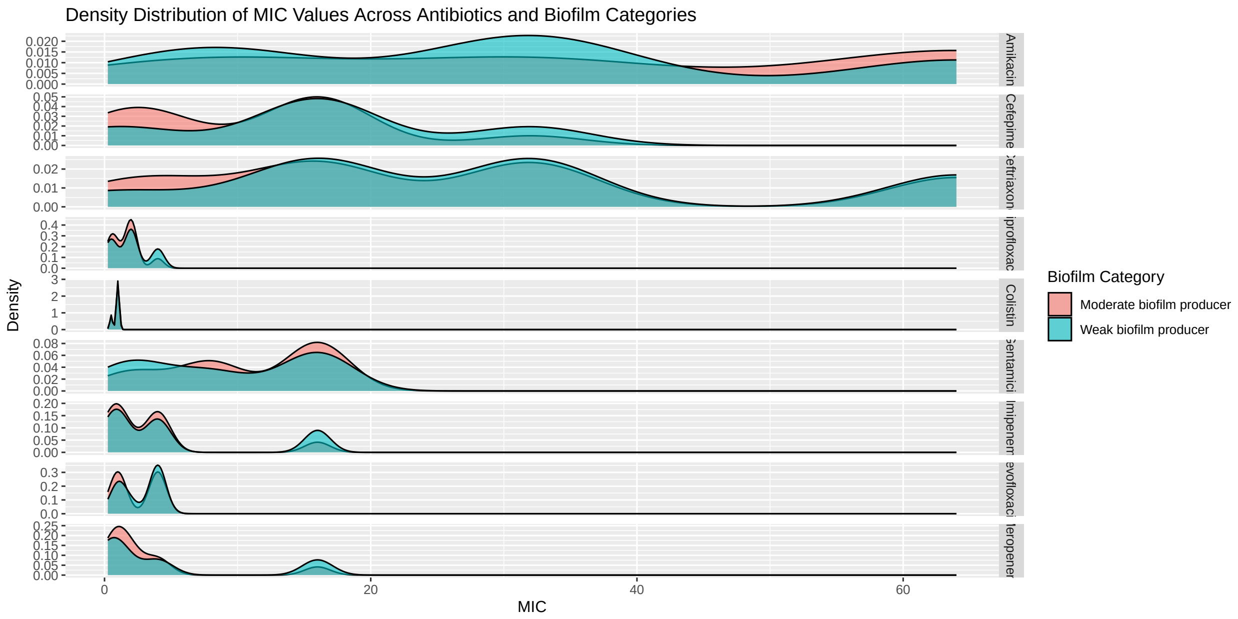

Ridge Plot (MIC and Biofilm Category)

Create a ridge plot to show the distribution of MIC values across

different biofilm categories and antibiotics

```
ggplot(data = df3.long, aes(x = MIC, y = Antibiotics, fill = Biofilm.category)) +
  geom_density_ridges(alpha = 0.6) +                 # Add density ridges with transparency
  labs(fill = "Biofilm Category") +
  scale_fill_brewer(palette = 'Set1')  # Use the Set1 color palette for the fill
```

```
## Picking joint bandwidth of 2.71
```

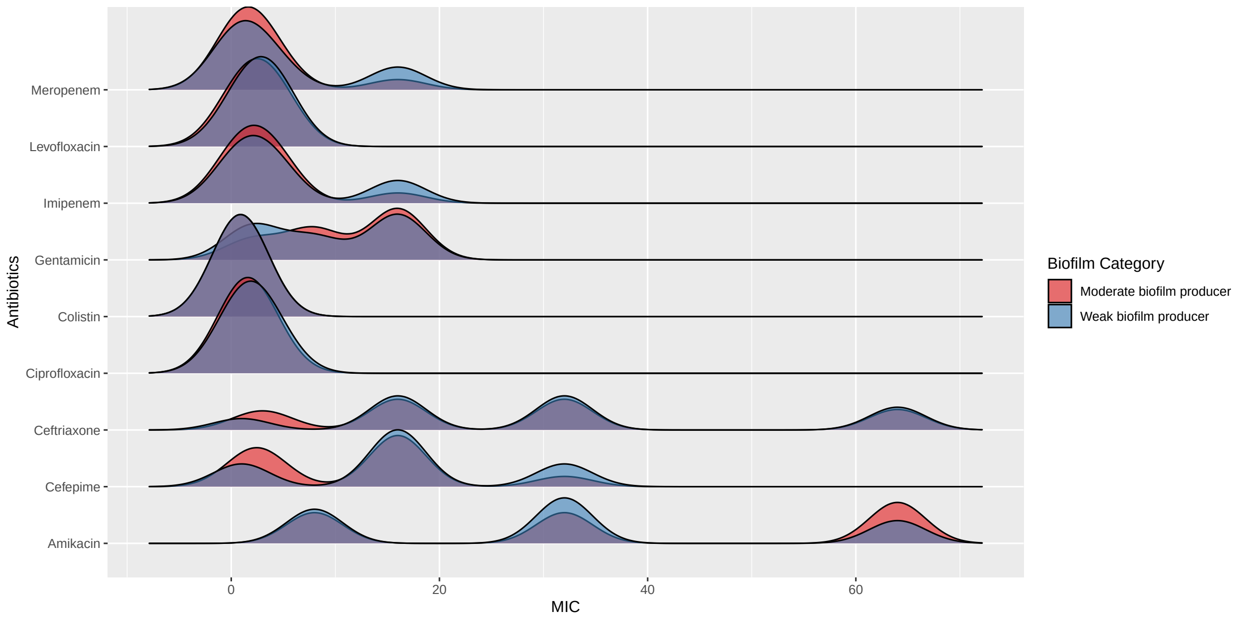

Boxplot for Biofilm Category and MrkA

Create a boxplot to visualize the effect of MrkA presence on biofilm optical density (OD) across different biofilm categories

```
ggplot(data = df, aes(x = Biofilm.category, y = OD, fill = MrkA)) +
  geom_boxplot(alpha = 0.6, size = 0.9, staplewidth = 0.3) + theme_classic() + scale_fill_brewer(palett
  ggtitle("mrkA effect on biofilm OD") + labs(fill = "mrkA presence", x = 'Biofilm Category') +
        scale_y_continuous(breaks = seq(0, 0.25, by = 0.02))
```

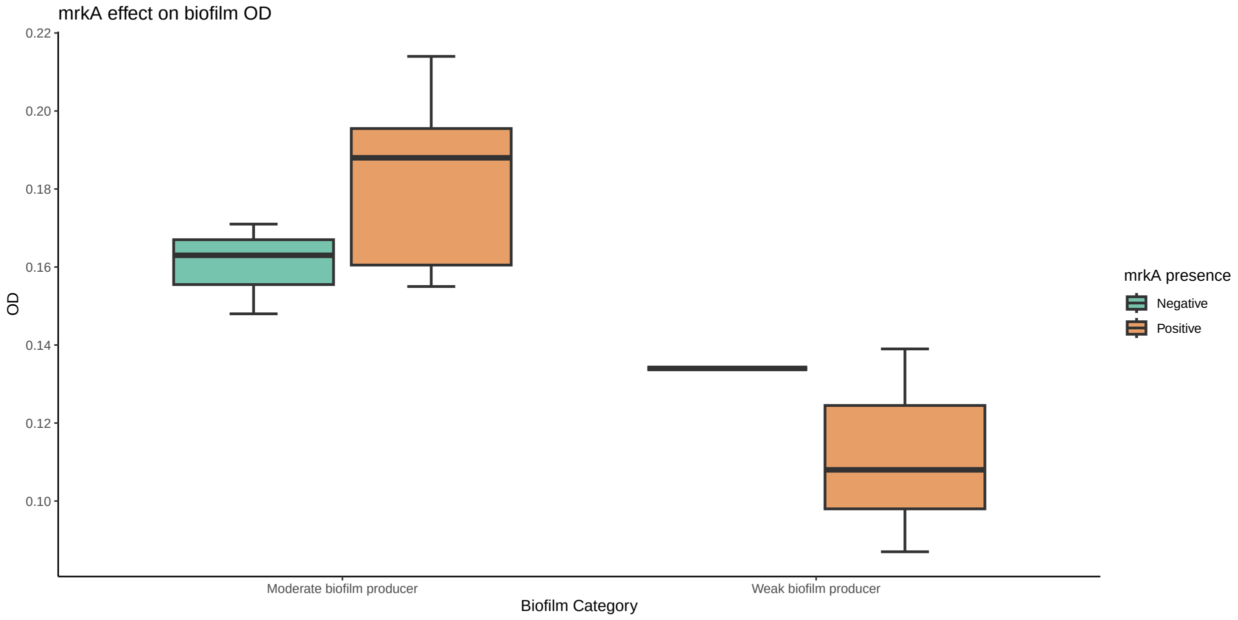

MrkA	Biofilm.category	mean(OD, na.rm = T)
Negative	Moderate biofilm producer	0.1606667
Negative	Weak biofilm producer	0.1340000
Positive	Moderate biofilm producer	0.1812857
Positive	Weak biofilm producer	0.1107500

Calculate Mean OD for MrkA and Biofilm.category

Group data by MrkA presence and biofilm category, then calculate the mean OD within each group

```
mean_od <- df %>%
  group_by(MrkA, Biofilm.category) %>%       # Group data by MrkA and Biofilm category
  summarise(mean(OD, na.rm = T), .groups = 'drop')  # Calculate mean OD, ignoring missing values
head(mean_od, 5) %>% gt()  # Display the summarized results
```

Load and View Raw Gene Expression Data

Read the dataset containing fluorescence values across multiple PCR cycles.

```
gene_exp <- read.csv("Gene_f_data.csv")  # Load gene expression data
head(gene_exp,2)  # View raw dataset
```

```
##   Sample.id Sample.id.1 Gene CT.Value           X1           X2           X3
## 1        29         pH5  TEM    14.25  0.003664158 0.002708658 0.002534171
## 2        29         pH5 mrkA    15.79 -0.001119816 0.001096974 0.000694002
##            X4           X5           X6           X7           X8           X9
## 1 0.001080477  0.000491324  0.000491324 -0.001993893 -0.003666275 -0.003132216
## 2 0.000895141 -0.000311401 -0.000208705  0.000776411  0.000776411  0.000513385
##           X10         X11        X12        X13        X14       X15       X16
## 1 0.000846798 0.009407605 0.03147078 0.07725016 0.17385996 0.3568631 0.6829096
## 2 0.001661716 0.004352812 0.01357826 0.02797575 0.06049471 0.1202811 0.2326206
##         X17      X18      X19      X20      X21      X22      X23      X24
## 1 1.1815952 1.804807 2.409750 2.876689 3.167608 3.323355 3.404278 3.460404
## 2 0.4302528 0.748080 1.195063 1.711647 2.201101 2.586575 2.850632 3.026864
##        X25      X26      X27      X28      X29      X30      X31      X32
## 1 3.490829 3.516170 3.533856 3.544375 3.551819 3.557362 3.562347 3.564783
## 2 3.145221 3.225353 3.284089 3.328077 3.362257 3.388991 3.405896 3.424108
##        X33      X34      X35      X36      X37      X38      X39      X40
## 1 3.565336 3.567022 3.567090 3.565567 3.563108 3.563380 3.560729 3.557976
## 2 3.434441 3.443859 3.453108 3.457416 3.461530 3.469013 3.467249 3.467204
```

Reshape Data to Long Format Convert wide-format fluorescence cycle data to long format for easier visualization.

```
gene_ex <- gene_exp %>%
  pivot_longer(
    cols = starts_with("X"),           # All fluorescence values (cycle data)
    values_to = 'Fluorecent',          # New column for fluorescence values
    names_to = 'Cycle'                 # New column for cycle identifiers
  )
```

Convert Sample.id to character to ensure proper grouping during visualization

```
gene_ex$Sample.id <- as.character(gene_ex$Sample.id)
```

Plot Fluorescence vs. Cycle Number by Gene and Sample

Visualize how fluorescence changes across PCR cycles for each gene and sample combination.

```
ggplot(data = gene_ex, aes(x = reorder(Cycle, +Fluorecent), y = Fluorecent)) +
  geom_line(aes(group = interaction(Sample.id, Gene), color = Gene), size = 1) +
  facet_grid(Sample.id.1 ~ ., scale = 'free') +
  scale_color_brewer(palette = 'Dark2') +
  theme_classic() +
  labs(x = 'Cycle Number', y = 'Fluorescent', shape = 'Sample Code') +
  geom_point(aes(shape = Sample.id), size = 2) +
  scale_x_discrete(labels = c(
    'X1' = '1', "X3" = '2', "X2" = '3', 'X7' = '4', 'X5' = '5',
    'X6' = '6', 'X4'= "7",
    'X8' = '8', "X9" = '9', "X10" = '10', 'X11' = '11', 'X12' =
            '12', 'X13' = '13', 'X14'= "14",
    'X15' = '15', "X16" = '16', "X17" = '17', 'X18' = '18',
    'X19' = '19', 'X20' = '20', 'X21'= "21",
    'X22' = '22', "X23" = '23', "X24" = '24', 'X25' = '25',
    'X26' = '26', 'X27' = '27', 'X28'= "28",
    'X29' = '29', "X30" = '30', "X31" = '31', 'X32' = '32',
    'X33' = '33', 'X34' = '34', 'X35'= "35",
    'X36' = '36', "X37" = '37', "X38" = '38', 'X39' = '39', 'X40' = '40'
  ))
```

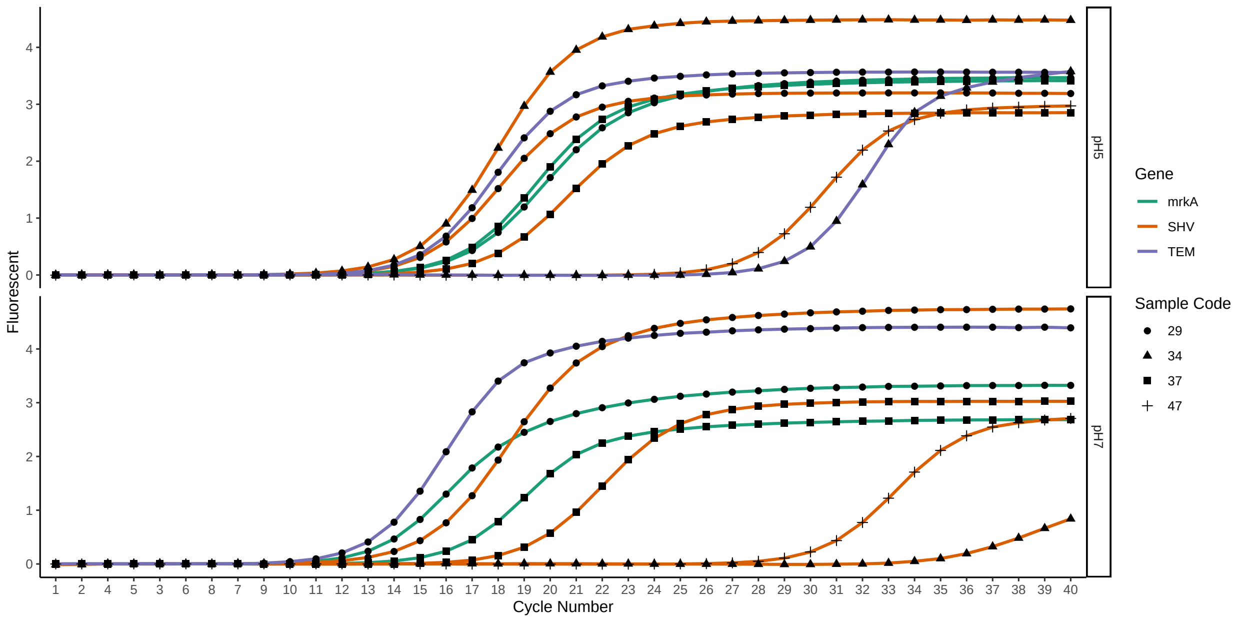

Perform K-Means Clustering

Apply unsupervised k-means clustering on fluorescence cycle data.

```
kmean_r <- kmeans(scale(gene_exp[, c(
  "X1", "X2", "X3", "X4", "X5", "X6", "X7", "X8", "X9", "X10",
  "X11", "X12", "X13", "X14", "X15", "X16", "X17", "X18", "X19", "X20",
  "X21", "X22", "X23", "X24", "X25", "X26", "X27", "X28", "X29", "X30",
  "X31", "X32", "X33", "X34", "X35", "X36", "X37", "X38", "X39", "X40"
)]), centers = 2)  # Using 2 clusters (can be adjusted)

# View distribution of samples across clusters
table(kmean_r$cluster)
```

```
##
## 1 2
## 8 7
```

Normality Test for MIC Values

Test whether Amikacin and Gentamicin MIC values are normally distributed using the Shapiro-Wilk test.

```
shapiro.test(df$Amikacin..MIC.)
```

```
##
##  Shapiro-Wilk normality test
##
## data:  df$Amikacin..MIC.
## W = 0.80206, p-value = 0.001224
```

```r
shapiro.test(df$Gentamicin..MIC.)
```

```
##
##  Shapiro-Wilk normality test
##
## data:  df$Gentamicin..MIC.
## W = 0.76439, p-value = 0.0003631
```

```r
shapiro.test(df$Colistin..MIC.)
```

```
##
##  Shapiro-Wilk normality test
##
## data:  df$Colistin..MIC.
## W = 0.50718, p-value = 5.816e-07
```

Kruskal-Wallis Test for MIC Values Across Biofilm Categories

Compare MIC distributions across biofilm-forming groups using non-parametric testing.

```r
kruskal.test(df$Gentamicin..MIC. ~ df$Biofilm.category)
```

```
##
##  Kruskal-Wallis rank sum test
##
## data:  df$Gentamicin..MIC. by df$Biofilm.category
## Kruskal-Wallis chi-squared = 0.19388, df = 1, p-value = 0.6597
```

```r
kruskal.test(df$Amikacin..MIC. ~ df$Biofilm.category)
```

```
##
##  Kruskal-Wallis rank sum test
##
## data:  df$Amikacin..MIC. by df$Biofilm.category
## Kruskal-Wallis chi-squared = 0.31667, df = 1, p-value = 0.5736
```

Kruskal-Wallis Test for All Numeric MICs Across Biofilm Categories

Loop through all numeric columns to test for differences in MICs among biofilm categories.

```r
numeric_col <- df %>% select(where(is.numeric))

table1 <- data.frame(
  column = character(),
  chi.squared = numeric(),
  p.value = numeric(),
  stringsAsFactors = FALSE
)
```

column	chi.squared	p.value
Age	0.48208955	0.4874773568
OD	13.51185250	0.0002370614
Cefepime..MIC.	0.33240166	0.5642483163
Ceftriaxone..MIC.	0.06452830	0.7994768506
Meropenem..MIC.	0.00000000	1.0000000000
Imipenem..MIC.	0.18554688	0.6666490509
Amikacin..MIC.	0.31666667	0.5736177761
Gentamicin..MIC.	0.19387755	0.6597087711
Ciprofloxacin..MIC.	0.15688073	0.6920447385
Levofloxacin..MIC.	0.21324355	0.6442369373
Colistin..MIC.	0.01333333	0.9080725553

```
for(col in names(numeric_col)){
  test_results <- kruskal.test(x = df[[col]], g = df$Biofilm.category)
  table1 <- rbind(table1, data.frame(
    column = col,
    chi.squared = test_results$statistic,
    p.value = test_results$p.value
  ))
}
table1 %>% gt()
```

Kruskal-Wallis Test for MICs by Age Category

Compare all numeric MIC values across patient age categories.

```
table2 <- data.frame(
  column = character(),
  chi.squared = numeric(),
  p.value = numeric(),
  stringsAsFactors = FALSE
)

for(col in colnames(numeric_col)){
  test_R <- kruskal.test(x = df[[col]], g = df$Age.cat)
  table2 <- rbind(table2, data.frame(
    column = col,
    chi.squared = test_R$statistic,
    p.value = test_R$p.value
  ))
}
table2 %>% gt()
```

Reshape Resistance Interpretation Data to Long Format

Prepare resistance level data for visualization using pivot_longer.

column	chi.squared	p.value
Age	16.593503	0.0008566651
OD	4.495466	0.2126950712
Cefepime..MIC.	3.011114	0.3899148534
Ceftriaxone..MIC.	2.562453	0.4641101051
Meropenem..MIC.	2.285056	0.5153898326
Imipenem..MIC.	0.609375	0.8942828676
Amikacin..MIC.	0.875000	0.8314563046
Gentamicin..MIC.	1.586939	0.6623549777
Ciprofloxacin..MIC.	3.317366	0.3452329721
Levofloxacin..MIC.	3.466210	0.3251711233
Colistin..MIC.	1.999286	0.5725549704

```
df2 <- df[, !sapply(df, is.numeric)]

df2_long <- df2 %>%
  pivot_longer(
    cols = starts_with('Cefepime') | starts_with('Ceftriaxone') | starts_with('Meropenem') |
           starts_with('Imipenem') | starts_with('Amikacin') | starts_with('Gentamicin') |
           starts_with('Ciprofloxacin') | starts_with('Levofloxacin') | starts_with('Colistin'),
    names_to = "Antibiotics",
    values_to = 'level'
  )
```

Plot: Resistance Levels by Age Category and Antibiotics

Visualize the distribution of antibiotic resistance levels by patient age group.

```
ggplot(data = df2_long, aes(x = Age.cat, fill = level)) +
  geom_bar(stat = 'count', position = 'stack') +
  facet_grid(Antibiotics ~ ., scales = 'free') +
  scale_fill_brewer(palette = 'Set2') +
  labs(title = "Antibiotic resistance and age category",
       x = "Age category", y = 'Number of Cases', fill = "Level of resistance")
```

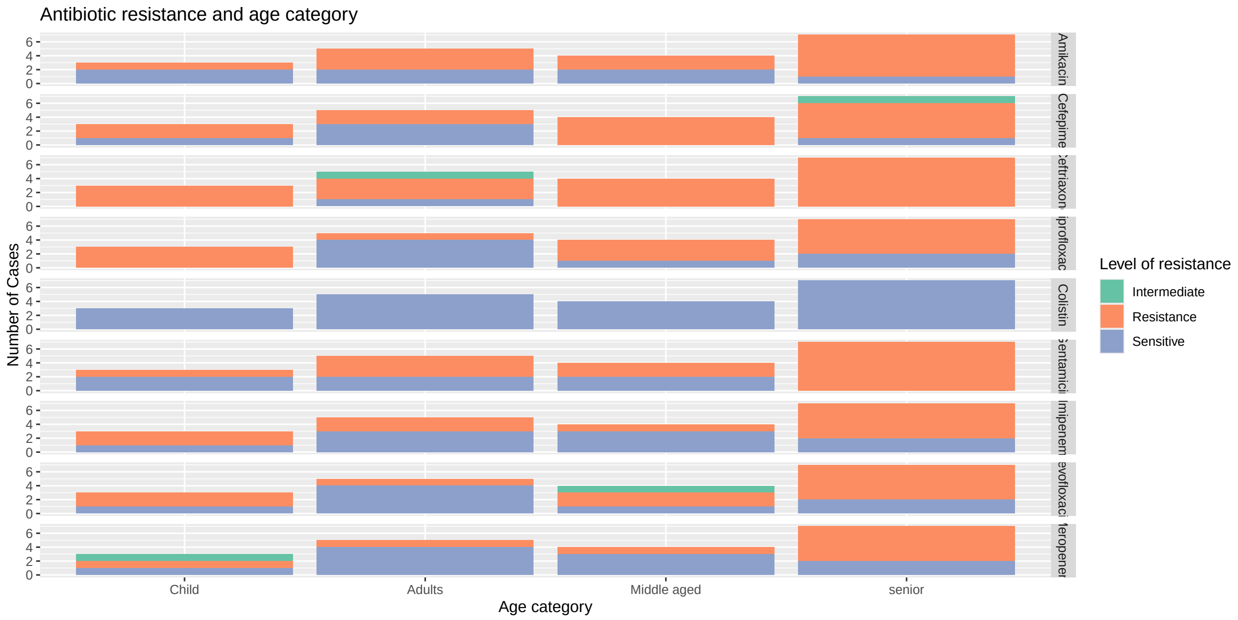

Clean Gene Presence Column and Filter

Remove unwanted whitespace and exclude MrkA from gene-wise analysis.

```
df3.long$`gene presence` <- trimws(df3.long$`gene presence`)
df3.long.f <- df3.long %>%
  filter(Genes != 'MrkA')
```

Plot: Gene Presence per Sample (Excluding MrkA)

Visualize gene presence (excluding MrkA) per sample across different genes.

```
ggplot(data = df3.long.f, aes(x = sample.code, fill = `gene presence`)) +
  geom_bar(position = 'stack') +
  facet_grid(Genes ~ ., scales = 'free') +
  scale_fill_brewer(palette = 'RdBu') +
  labs(x = 'Sample Code', fill = 'Gene Presence')
```

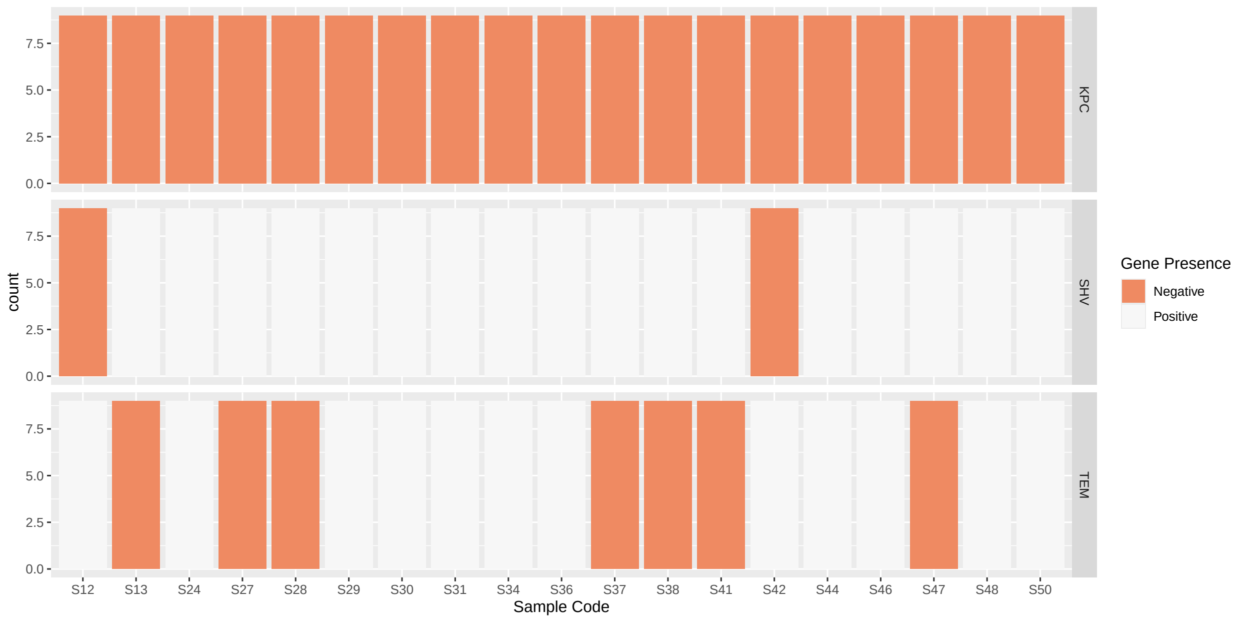

Fisher's Exact Test for Association Between Antibiotics and Biofilm Category

```
# Extract character columns for Fisher's Test
cat_df <- df %>%
    select(where(is.character)) # Display the data frame with categorical variables

# Add the Biofilm category to the data frame
cat_df$Biofilm.category <- df$Biofilm.category

# Subset the data to focus on antibiotic columns and Biofilm category
cat_df2 <- cat_df[, c("Cefepime", "Ceftriaxone", "Meropenem", "Imipenem",
                      "Amikacin", "Gentamicin", "Ciprofloxacin", "Levofloxacin",
                      "Biofilm.category")]

# Initialize an empty data frame to store Fisher's test results
fisher_table <- data.frame(
    column = character(),
    p.value = numeric(),
    alternative.hypo = character(),
    stringsAsFactors = F
)

# Perform Fisher's Exact Test for each antibiotic column
for(col in colnames(cat_df2)){
    # Create a contingency table for the antibiotic and TEM gene
    table_con <- table(cat_df$TEM, cat_df[,col])

    # Perform Fisher's Exact Test
    test_out <- fisher.test(table_con)

    # Store the test results in the fisher_table data frame
```

column	p.value	alternative.hypo
Cefepime	1.0000000	two.sided
Ceftriaxone	0.6140351	two.sided
Meropenem	0.3265063	two.sided
Imipenem	1.0000000	two.sided
Amikacin	0.3260895	two.sided
Gentamicin	0.1287410	two.sided
Ciprofloxacin	1.0000000	two.sided
Levofloxacin	1.0000000	two.sided
Biofilm.category	0.6499166	two.sided

```
    fisher_table <- rbind(fisher_table, data.frame(
        column = col,
        p.value = test_out$p.value,
        alternative.hypo = test_out$alternative
    ))
}
# Print the results of the Fisher's Exact Tests
fisher_table %>% gt()
```

Additional Analysis

```
# Heatmap to visualize correlations or patterns between numeric variables
pheatmap(as.matrix(numeric_col), border_color = 'black',
    color = colorRampPalette(c('grey','white','yellow'))(100),
    main = 'Heatmap', show_rownames = T,
    display_numbers = T,
    number_color = 'black')
```

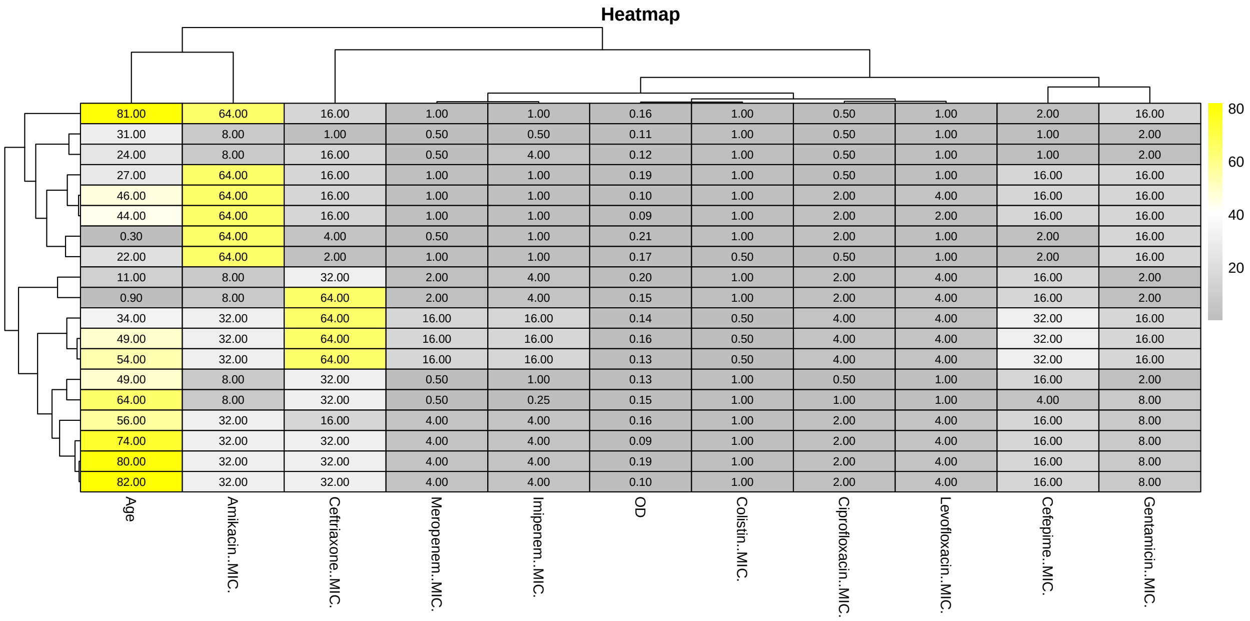


```r
# Principal Component Analysis (PCA) for dimensionality reduction
pca <- prcomp(scale(numeric_col), scale = TRUE)  # Perform PCA with scaling
summary(pca)  # Summary of PCA results
```

```
## Importance of components:
##                           PC1    PC2    PC3     PC4     PC5     PC6     PC7
## Standard deviation     2.3472 1.4570 1.1943 0.86065 0.77978 0.50012 0.39293
## Proportion of Variance 0.5009 0.1930 0.1297 0.06734 0.05528 0.02274 0.01404
## Cumulative Proportion  0.5009 0.6938 0.8235 0.89085 0.94613 0.96886 0.98290
##                            PC8     PC9    PC10    PC11
## Standard deviation     0.34920 0.21932 0.11819 0.06403
## Proportion of Variance 0.01109 0.00437 0.00127 0.00037
## Cumulative Proportion  0.99398 0.99836 0.99963 1.00000
```

```r
# Scree plot to visualize the proportion of variance explained by each principal component
pca_var <- pca$sdev^2  # Variance of each principal component
pca_var_explained <- pca_var / sum(pca_var)  # Proportion of variance explained by each component

plot(pca_var_explained, type = "b", xlab = "Principal Component",
    ylab = "Proportion of Variance Explained", main = "Scree Plot")
```

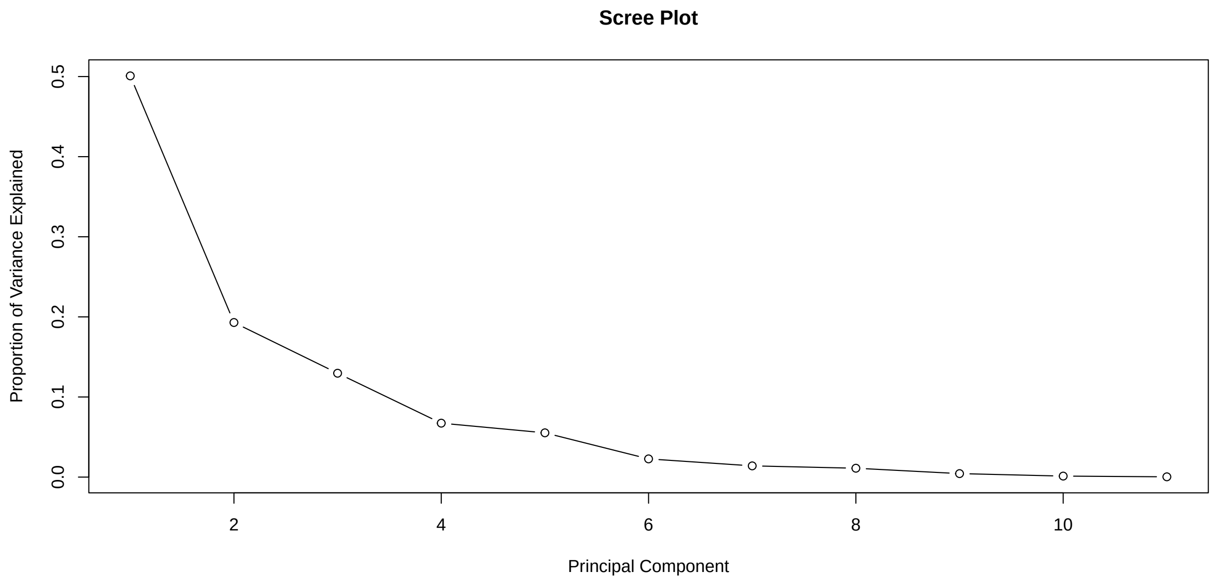


```r
# Scatter plot for the first two principal components (PC1 vs PC2)
pca_data <- as.data.frame(pca$x)  # Extract PCA scores
ggplot(pca_data, aes(x = PC1, y = PC2)) +
    geom_point() +
    theme_minimal() +
    labs(title = "PCA: PC1 vs PC2", x = "PC1", y = "PC2")
```

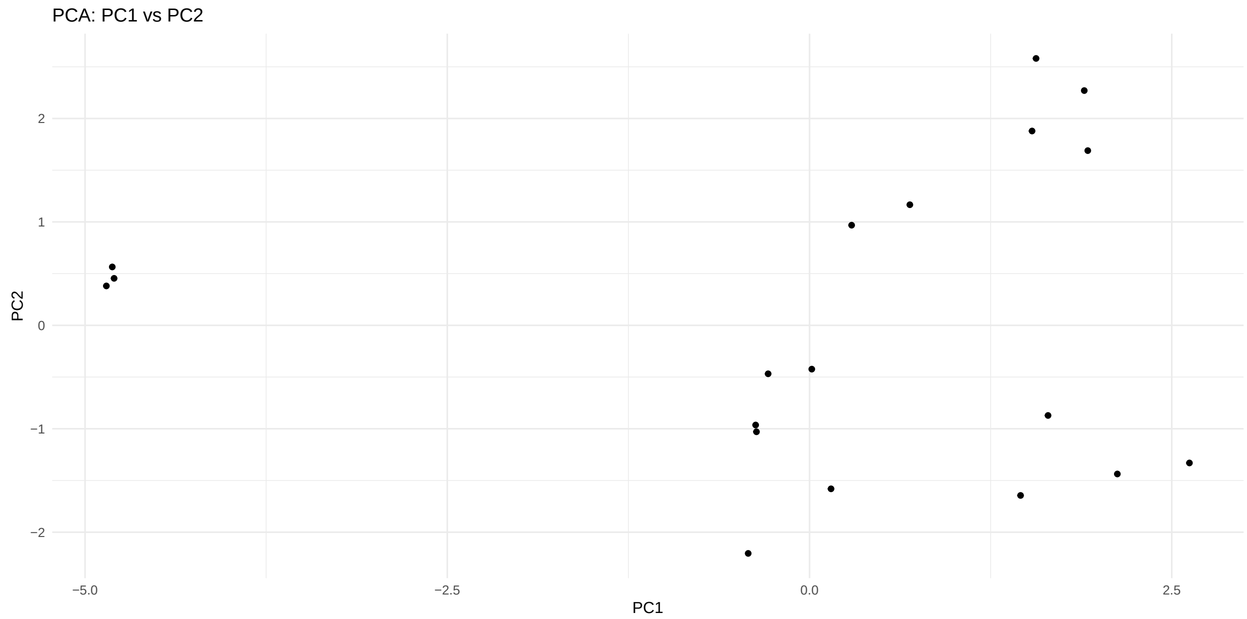


```r
## Kruskal-Wallis Test for Antibiotic Resistance and TEM Gene

# Initialize a data frame to store Kruskal-Wallis test results for numeric columns
table1.1 <- data.frame(
    column = character(),
```

column	chi.squared	p.value
Age	3.15276558	0.07579840
OD	0.57907939	0.44667340
Cefepime..MIC.	0.76075865	0.38309121
Ceftriaxone..MIC.	0.37641509	0.53952809
Meropenem..MIC.	0.82741935	0.36301981
Imipenem..MIC.	0.12723214	0.72131929
Amikacin..MIC.	1.35714286	0.24403326
Gentamicin..MIC.	3.32361516	0.06829150
Ciprofloxacin..MIC.	1.09775011	0.29476039
Levofloxacin..MIC.	0.02056277	0.88597646
Colistin..MIC.	2.80000000	0.09426431

```
    chi.squared = numeric(),
    p.value = numeric(),
    stringsAsFactors = F
)

# Perform Kruskal-Wallis Test for each numeric column against TEM gene
for(col in colnames(numeric_col)){
    test_k <- kruskal.test(x = df[[col]], g = df$TEM)  # Kruskal-Wallis test
    table1.1 <- rbind(table1.1, data.frame(
        column = col,
        chi.squared = test_k$statistic,
        p.value = test_k$p.value
    ))
}
# Print the results of the Kruskal-Wallis Tests
table1.1 %>% gt()
```

```
## Normality Tests and Q-Q Plots

# Read data files for further analysis
col_sam <- read.csv("Samples.csv")
all <- read.csv("all_variables.csv")

# Subset numeric columns for normality testing
num <- all %>%
    select(where(is.numeric))

colnames(num) <- c("Age", "OD", "Cefepime MIC", "Ceftriaxone MIC", "Meropenem MIC",
    "Imipenem MIC", "Amikacin MIC", "Gentamicin MIC", "Ciprofloxacin MIC", "Levofloxacin MIC",
    "Colistin MIC")

# Perform Shapiro-Wilk normality test for each numeric column
for(col in colnames(num)){
    test <- shapiro.test(num[,col])  # Shapiro-Wilk test for normality
    print(col)
    print(test)
```

```
}
```

```
## [1] "Age"
##
##  Shapiro-Wilk normality test
##
## data:  num[, col]
## W = 0.95389, p-value = 0.4589
##
## [1] "OD"
##
##  Shapiro-Wilk normality test
##
## data:  num[, col]
## W = 0.97035, p-value = 0.7835
##
## [1] "Cefepime MIC"
##
##  Shapiro-Wilk normality test
##
## data:  num[, col]
## W = 0.82096, p-value = 0.002342
##
## [1] "Ceftriaxone MIC"
##
##  Shapiro-Wilk normality test
##
## data:  num[, col]
## W = 0.85352, p-value = 0.007657
##
## [1] "Meropenem MIC"
##
##  Shapiro-Wilk normality test
##
## data:  num[, col]
## W = 0.62489, p-value = 8.03e-06
##
## [1] "Imipenem MIC"
##
##  Shapiro-Wilk normality test
##
## data:  num[, col]
## W = 0.65812, p-value = 1.835e-05
##
## [1] "Amikacin MIC"
##
##  Shapiro-Wilk normality test
##
## data:  num[, col]
## W = 0.80206, p-value = 0.001224
##
## [1] "Gentamicin MIC"
##
```

```
##  Shapiro-Wilk normality test
##
## data:  num[, col]
## W = 0.76439, p-value = 0.0003631
##
## [1] "Ciprofloxacin MIC"
##
##  Shapiro-Wilk normality test
##
## data:  num[, col]
## W = 0.80632, p-value = 0.001414
##
## [1] "Levofloxacin MIC"
##
##  Shapiro-Wilk normality test
##
## data:  num[, col]
## W = 0.66787, p-value = 2.359e-05
##
## [1] "Colistin MIC"
##
##  Shapiro-Wilk normality test
##
## data:  num[, col]
## W = 0.50718, p-value = 5.816e-07
```

```
# Generate Q-Q plots for each numeric column
for(col in colnames(num)){
    qqnorm(num[,col], main = paste("Q-Q plot of", col))  # Create Q-Q plot
    qqline(num[,col], col = 'red')  # Add a reference line to the Q-Q plot
}
```

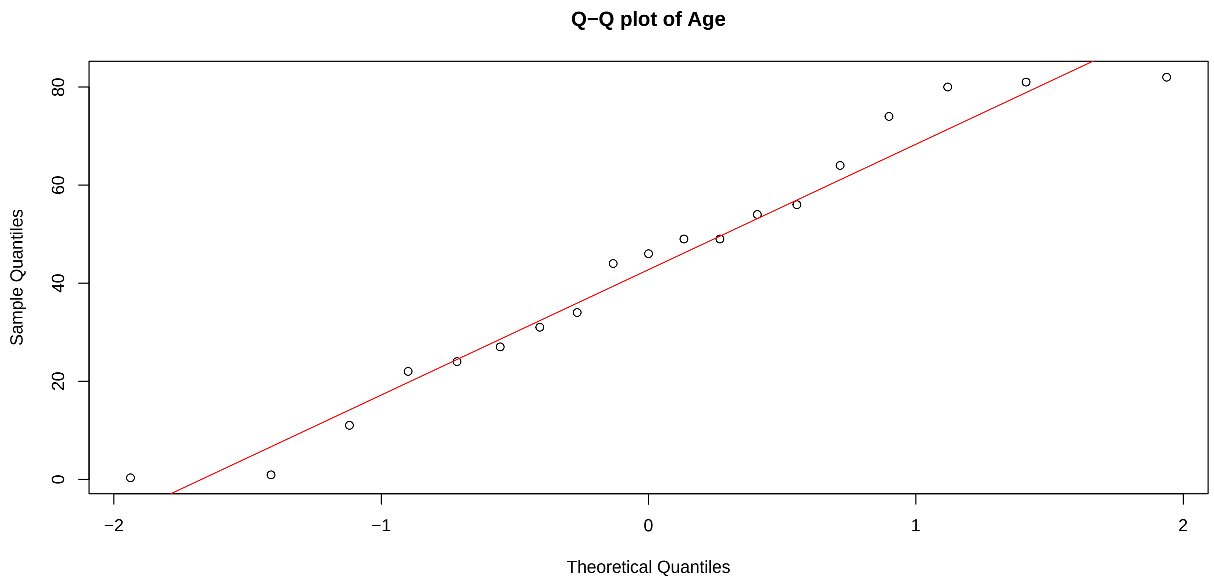

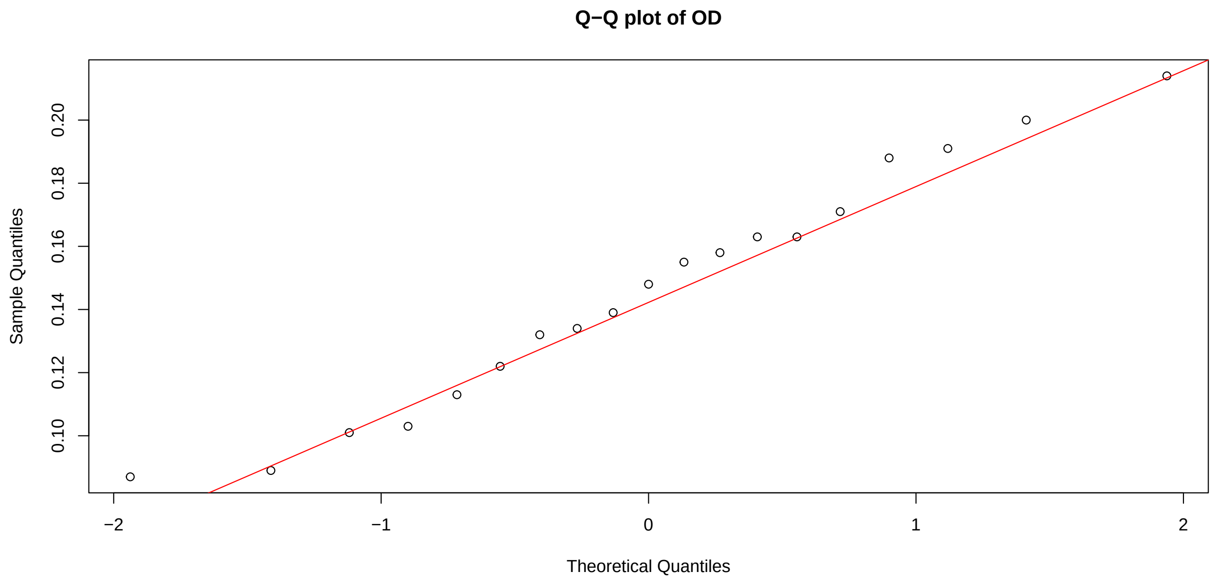
Q−Q plot of OD
Sample Quantiles
0.10
0.12
0.14
0.16
0.18
0.20
−2
−1
0
1
2
Theoretical Quantiles

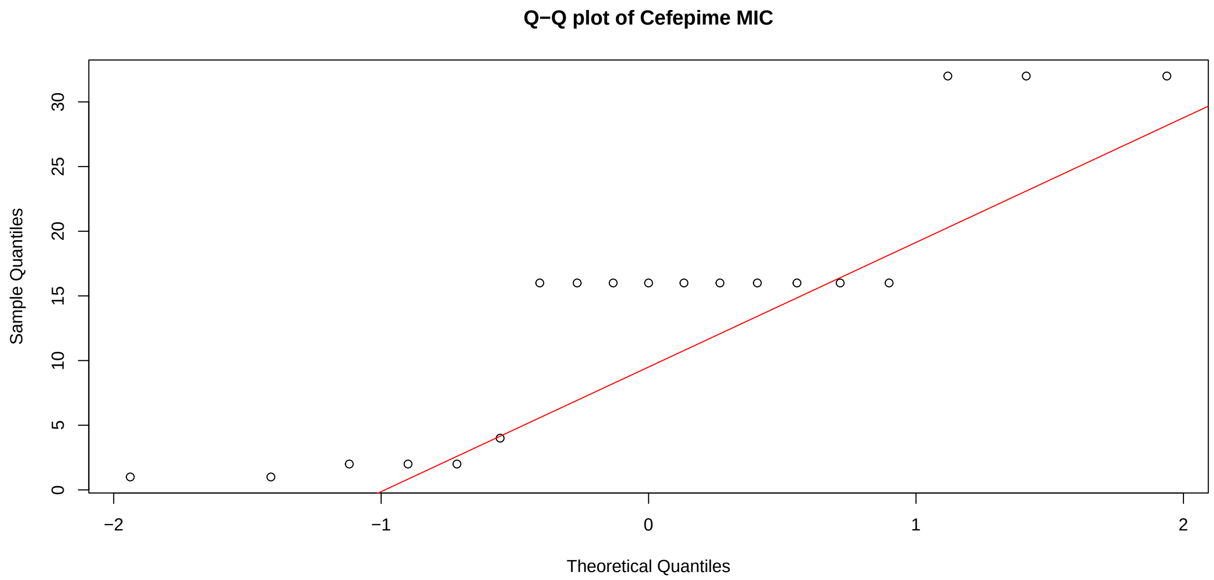
Q−Q plot of Cefepime MIC
Sample Quantiles
0
5
10
15
20
25
30
−2
−1
0
1
2
Theoretical Quantiles

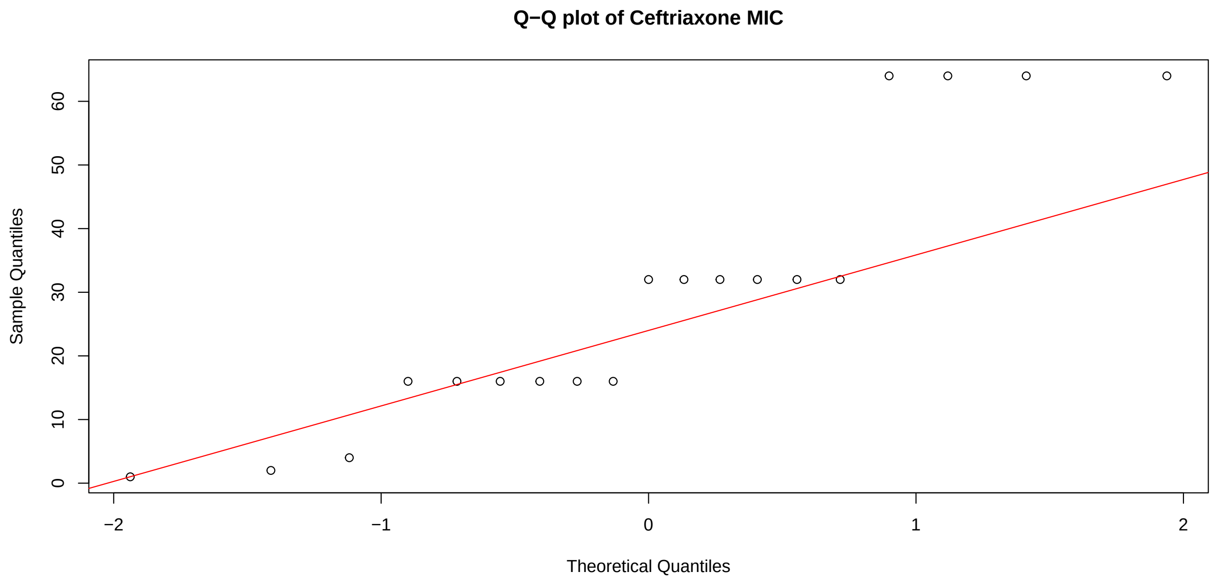
Q−Q plot of Ceftriaxone MIC
Sample Quantiles
Theoretical Quantiles
0
10
20
30
40
50
60
−2
−1
0
1
2

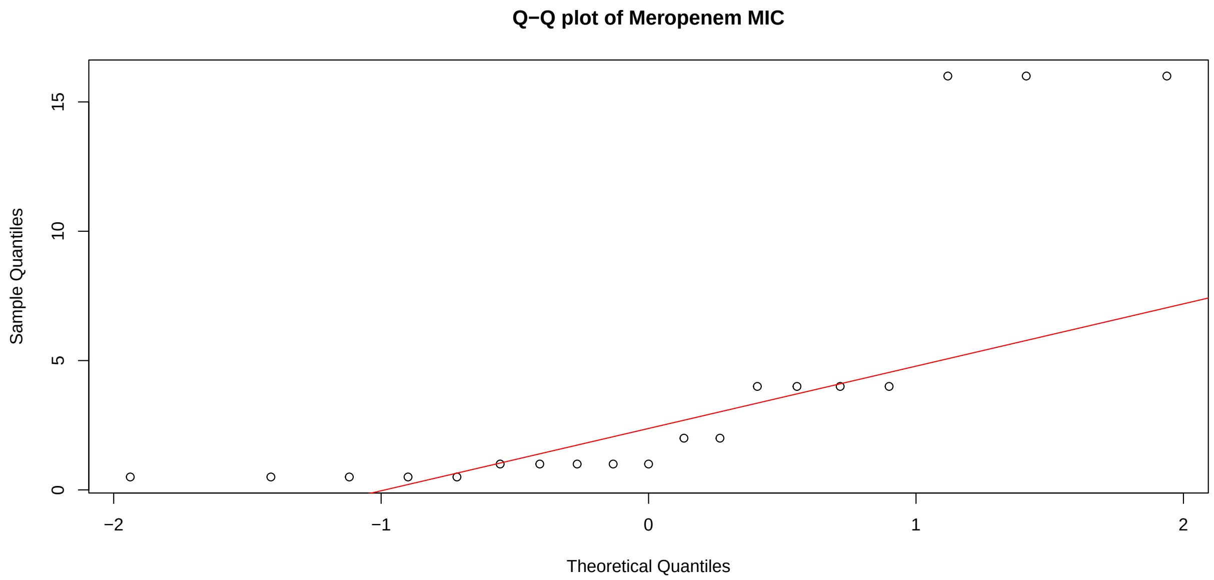
Q−Q plot of Meropenem MIC
Sample Quantiles
Theoretical Quantiles
0
5
10
15
−2
−1
0
1
2

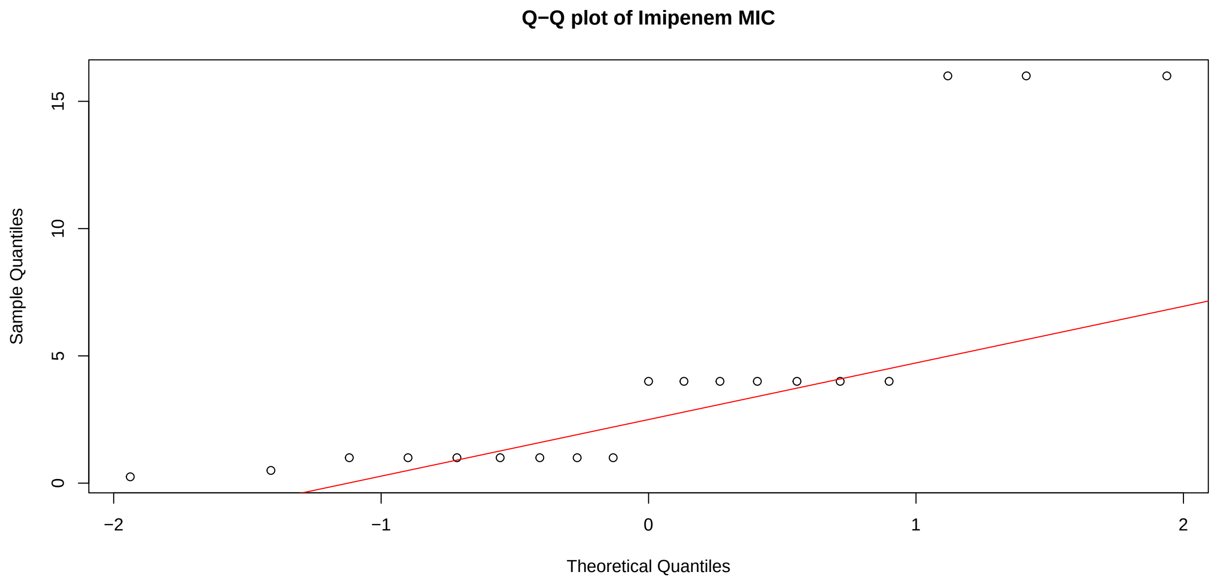
Q−Q plot of Imipenem MIC
Sample Quantiles
Theoretical Quantiles
0
5
10
15
−2
−1
0
1
2

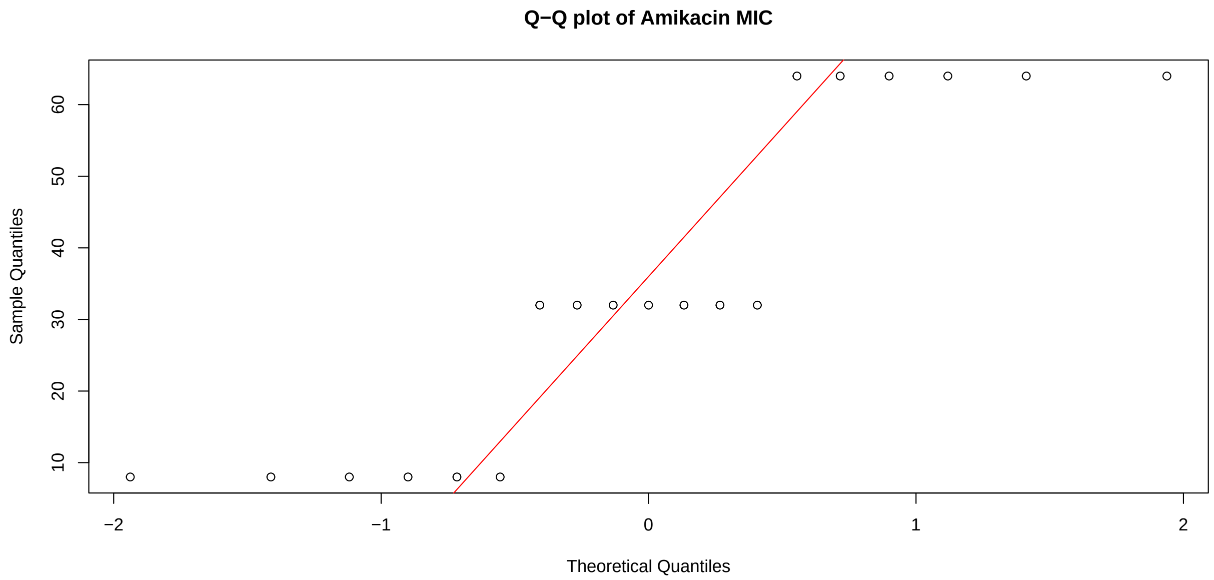
Q−Q plot of Amikacin MIC
Sample Quantiles
Theoretical Quantiles
10
20
30
40
50
60
−2
−1
0
1
2

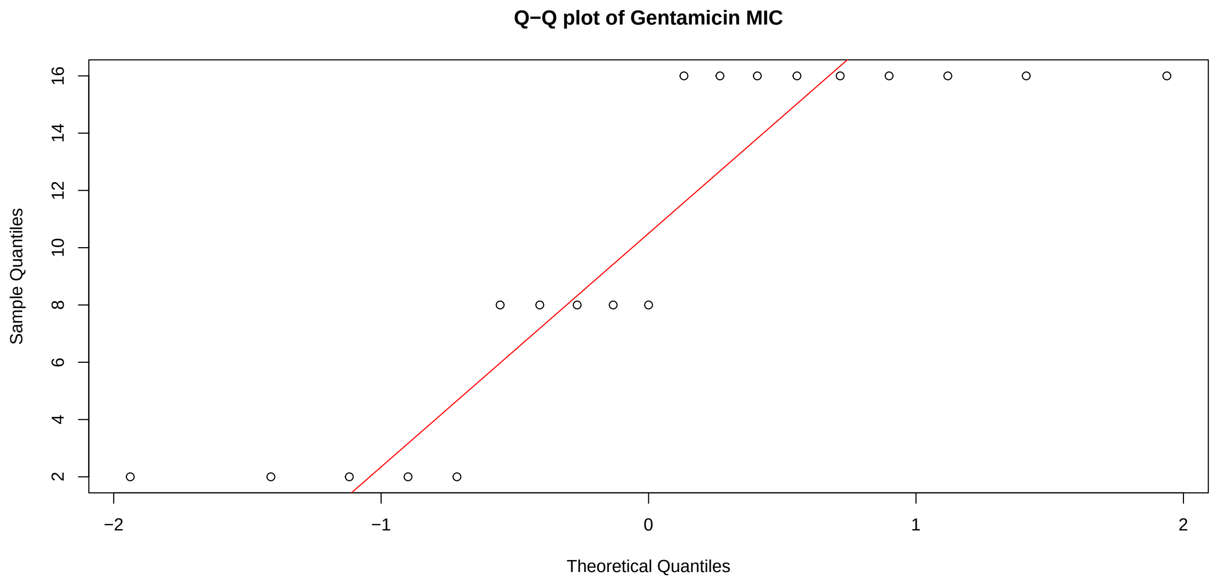
Q−Q plot of Gentamicin MIC
Sample Quantiles
Theoretical Quantiles

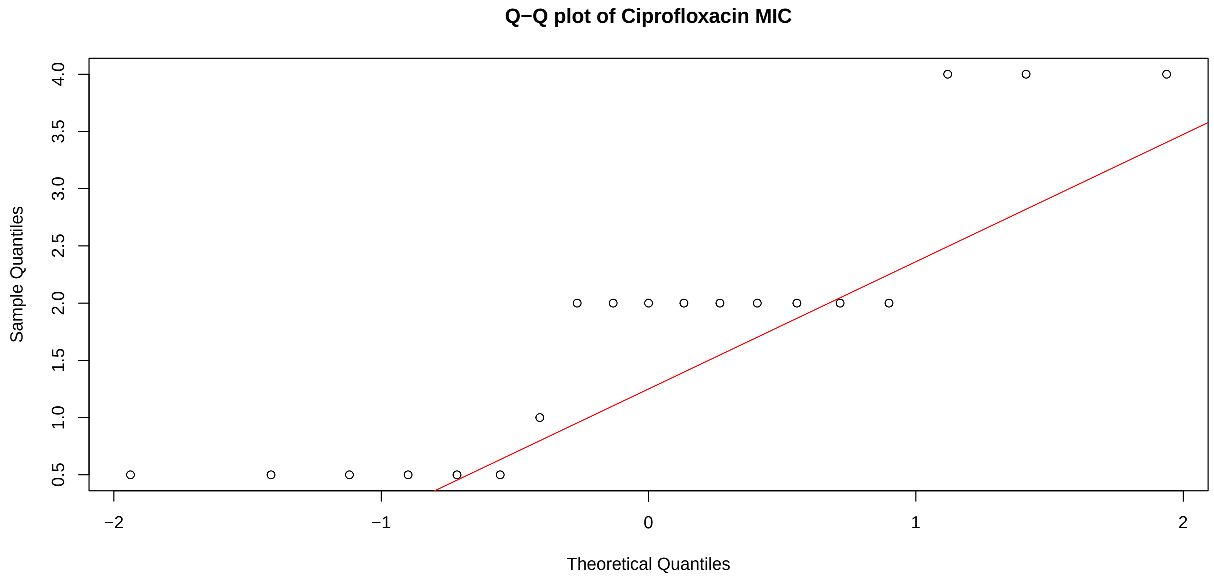
Q−Q plot of Ciprofloxacin MIC
Sample Quantiles
Theoretical Quantiles

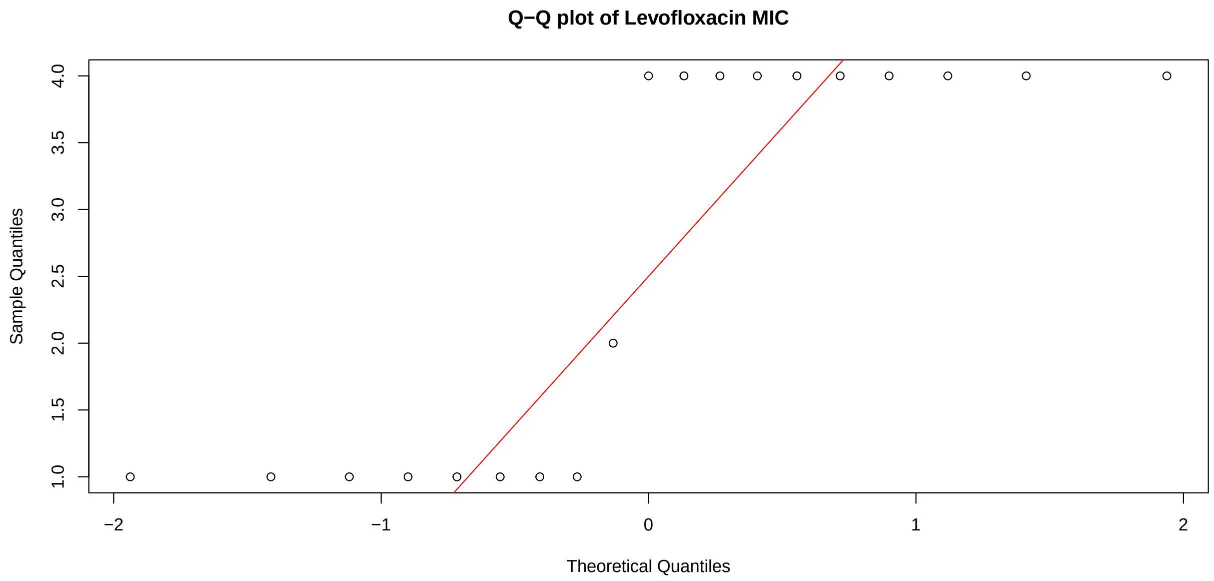

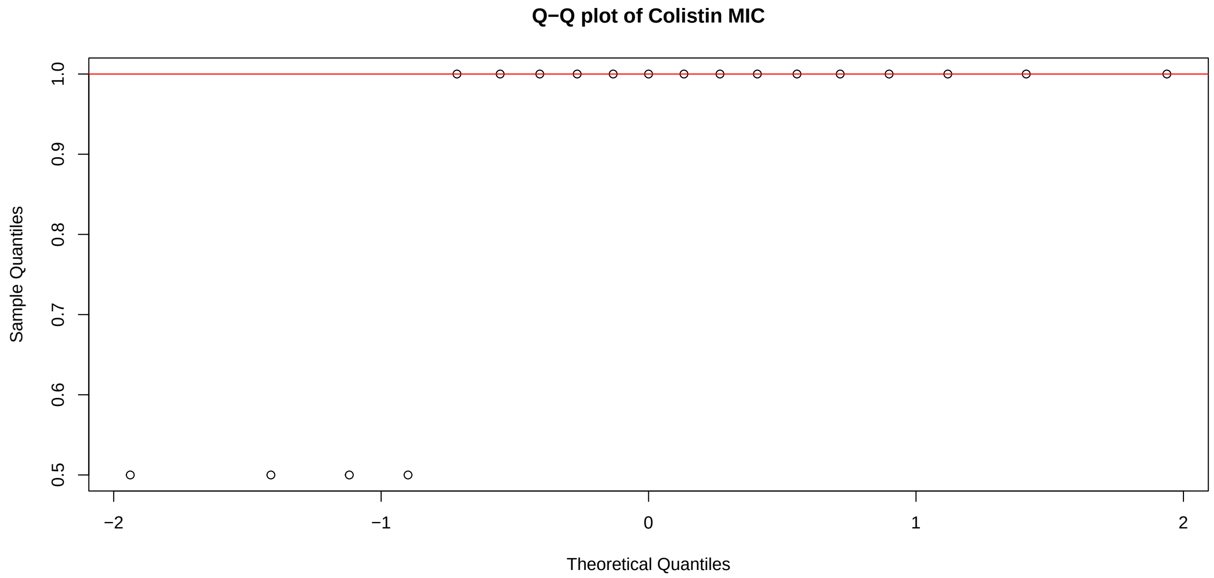


```r
## Kruskal-Wallis Test for Age and Sample Area/Gender

# Perform Kruskal-Wallis test for Age by Sample Area
kruskal.test(col_sam$Age~col_sam$Sample.Area)
```

```
##
##  Kruskal-Wallis rank sum test
##
## data:  col_sam$Age by col_sam$Sample.Area
## Kruskal-Wallis chi-squared = 23.017, df = 19, p-value = 0.2366
```

```r
# Perform Kruskal-Wallis test for Age by Gender
kruskal.test(col_sam$Age~col_sam$Gender)
```

```
##
##  Kruskal-Wallis rank sum test
##
## data:  col_sam$Age by col_sam$Gender
## Kruskal-Wallis chi-squared = 1.027, df = 1, p-value = 0.3109
```

Antibiotic Susceptibility Analysis

Analyze numerical antibiotic response data and test for differences across sample areas using Kruskal-Wallis tests.

```r
# Load antibiotic dataset
anti <- read.csv("antibiotics.csv")

# Select only numerical columns for non-parametric testing
an.num <- anti %>%
    select(where(is.numeric))

# Initialize table to store Kruskal-Wallis test results
tableJ <- data.frame(
    column = character(),
    chi.squared = numeric(),
    p.value = numeric(),
    df = numeric(),
    stringsAsFactors = F
)

# Perform Kruskal-Wallis test for each numeric antibiotic variable by Sample area
for(col in colnames(an.num)){
    kir1 <- kruskal.test(anti[,col] ~ anti$Sample.area)
    tableJ <- rbind(tableJ, data.frame(
        column = col,
        chi.squared = kir1$statistic,
        p.value = kir1$p.value,
        df = kir1$parameter
    ))
}
tableJ %>% gt()
```

Resistance Gene Association Analysis

Check whether resistance gene presence (e.g., SHV) correlates with numeric antibiotic response using Kruskal-Wallis test.

```r
# Load antibiotic resistance gene dataset
anti2 <- read.csv("antibiotics_resistance_genes.csv")
```

column	chi.squared	p.value	df
Age	8.549166	0.3817414	8
Cefepime..MIC.	9.359731	0.3128640	8
Ceftriaxone..MIC.	10.407547	0.2375780	8
Meropenem..MIC.	9.286175	0.3187328	8
Imipenem..MIC.	9.765625	0.2818629	8
Amikacin..MIC.	6.916667	0.5456499	8
Gentamicin..MIC.	7.877551	0.4455219	8
Ciprofloxacin..MIC.	9.816514	0.2781412	8
Levofloxacin..MIC.	7.890011	0.4442874	8
Colistin..MIC.	12.933333	0.1141641	8

```r
# Select only numeric columns for analysis
num_anti <- anti2 %>%
    select(where(is.numeric))

# Initialize results table for Kruskal-Wallis output
table2.1 <- data.frame(
    column = character(),
    chi.squared = numeric(),
    p.value = numeric(),
    df = numeric(),
    stringsAsFactors = F
)

# Run Kruskal-Wallis test for each numeric antibiotic column vs SHV gene presence
for(col in colnames(num_anti)){
    kir3 <- kruskal.test(anti2[,col] ~ anti2$SHV)
    table2.1 <- rbind(table2.1, data.frame(
        column = col,
        chi.squared = kir3$statistic,
        p.value = kir3$p.value,
        df = kir3$parameter
    ))
}
table2.1 %>% gt()
```

```r
# Fisher test

df_anti <- df[,c('MrkA','SHV','TEM')]
df_anti[,'MrkA'] <- ifelse(df_anti[,'MrkA'] == 'Positive ', 'mrkA-Positive', 'mrkA-Negative')
df_anti[,'SHV'] <- ifelse(df_anti[,'SHV'] == 'Positive', 'blaSHV-Positive', 'blaSHV-Negative')
df_anti[,'TEM'] <- ifelse(df_anti[,'TEM'] == 'Positive ', 'blaTEM-Positive', 'blaTEM-Negative')

table1 <- table(df_anti[,'MrkA'], df_anti[,'SHV'])
table2 <- table(df_anti[,'MrkA'], df_anti[,'TEM'])

fisher.test(table1)
```

column	chi.squared	p.value	df
Age	1.59404534	0.20674912	1
Cefepime..MIC.	0.42172086	0.51607961	1
Ceftriaxone..MIC.	0.01897891	0.89042696	1
Meropenem..MIC.	1.50187043	0.22038379	1
Imipenem..MIC.	1.25735294	0.26215274	1
Amikacin..MIC.	0.00000000	1.00000000	1
Gentamicin..MIC.	0.08211285	0.77445431	1
Ciprofloxacin..MIC.	3.46573125	0.06265280	1
Levofloxacin..MIC.	2.73202614	0.09835444	1
Colistin..MIC.	0.56470588	0.45237036	1

```
##
##  Fisher's Exact Test for Count Data
##
## data:  table1
## p-value = 0.03509
## alternative hypothesis: true odds ratio is not equal to 1
## 95 percent confidence interval:
##  0.8102566       Inf
## sample estimates:
## odds ratio
##        Inf
```

```
fisher.test(table2)
```

```
##
##  Fisher's Exact Test for Count Data
##
## data:  table2
## p-value = 1
## alternative hypothesis: true odds ratio is not equal to 1
## 95 percent confidence interval:
##  0.008187916 8.421036732
## sample estimates:
## odds ratio
##  0.5174438
```

```
par(mfrow = c(1,2))
mosaicplot(table1, shade = T, main = 'Mosaicplot mrkA & blaSHV',
           cex.axis = 0.9)
mosaicplot(table2, shade =T, main = 'Mosaicplot mrkA & blaTEM',
           cex.axis = 0.9)
```

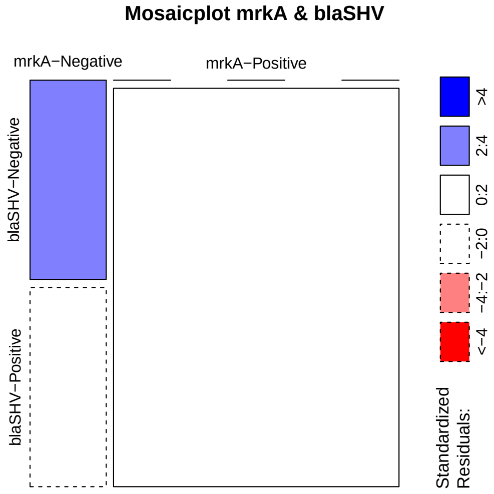

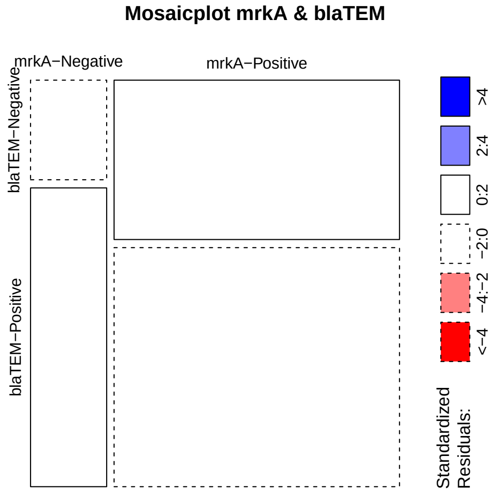

Bioflim Optical Density Analysis

Explore the influence of categorical variables and gene presence on biofilm formation using ANOVA.

```r
# Load biofilm dataset
bio <- read.csv("biofilm_data.csv")

# Select only categorical variables
char_bio <- bio %>%
    select(where(is.character)) %>%
        select(!Colistin)

# One-way ANOVA: test OD differences across levels of each categorical variable
for(col in colnames(char_bio)){
    anova <- aov(bio$OD ~ bio[,col])
    print(col)
    print(summary(anova))
}
```

```
## [1] "sample.code"
##              Df  Sum Sq  Mean Sq
## bio[, col]   18 0.02542 0.001412
## [1] "Gender"
##              Df   Sum Sq   Mean Sq F value Pr(>F)
## bio[, col]    1 0.000022 0.0000221   0.015  0.905
## Residuals    17 0.025396 0.0014939
## [1] "Sample.area"
##              Df  Sum Sq  Mean Sq F value Pr(>F)
## bio[, col]    8 0.01083 0.001354   0.928  0.533
## Residuals    10 0.01459 0.001459
## [1] "MrkA"
##              Df   Sum Sq   Mean Sq F value Pr(>F)
```

```
## bio[, col]   1 0.000337 0.0003372   0.229  0.639
## Residuals   17 0.025081 0.0014754
## [1] "Biofilm.category"
##             Df   Sum Sq  Mean Sq F value  Pr(>F)
## bio[, col]   1 0.018072 0.018072   41.82 5.8e-06 ***
## Residuals   17 0.007347 0.000432
## ---
## Signif. codes:  0 '***' 0.001 '**' 0.01 '*' 0.05 '.' 0.1 ' ' 1
## [1] "Cefepime"
##             Df   Sum Sq   Mean Sq F value Pr(>F)
## bio[, col]   2 0.000967 0.0004837   0.316  0.733
## Residuals   16 0.024451 0.0015282
## [1] "Ceftriaxone"
##             Df   Sum Sq   Mean Sq F value Pr(>F)
## bio[, col]   2 0.001715 0.0008575   0.579  0.572
## Residuals   16 0.023704 0.0014815
## [1] "Meropenem"
##             Df   Sum Sq  Mean Sq F value Pr(>F)
## bio[, col]   2 0.000006 0.000003   0.002  0.998
## Residuals   16 0.025413 0.001588
## [1] "Imipenem"
##             Df   Sum Sq  Mean Sq F value Pr(>F)
## bio[, col]   1 0.000088 0.000088   0.059  0.811
## Residuals   17 0.025330 0.001490
## [1] "Amikacin"
##             Df   Sum Sq   Mean Sq F value Pr(>F)
## bio[, col]   1 0.000811 0.0008114   0.561  0.464
## Residuals   17 0.024607 0.0014475
## [1] "Gentamicin"
##             Df   Sum Sq  Mean Sq F value Pr(>F)
## bio[, col]   1 0.001162 0.001161   0.814   0.38
## Residuals   17 0.024257 0.001427
## [1] "Ciprofloxacin"
##             Df   Sum Sq   Mean Sq F value Pr(>F)
## bio[, col]   1 0.000179 0.0001787    0.12  0.733
## Residuals   17 0.025240 0.0014847
## [1] "Levofloxacin"
##             Df   Sum Sq  Mean Sq F value Pr(>F)
## bio[, col]   2 0.004545 0.002272   1.742  0.207
## Residuals   16 0.020874 0.001305
```

```r
# Two-way ANOVA: test interaction between MrkA and Biofilm category on OD
anova_result <- aov(OD ~ MrkA * Biofilm.category, data = bio)
summary(anova_result)
```

```
##                       Df   Sum Sq  Mean Sq F value   Pr(>F)
## MrkA                   1 0.000337 0.000337   0.847    0.372
## Biofilm.category       1 0.017906 0.017906  44.962 7.04e-06 ***
## MrkA:Biofilm.category  1 0.001202 0.001202   3.018    0.103
## Residuals             15 0.005974 0.000398
## ---
## Signif. codes:  0 '***' 0.001 '**' 0.01 '*' 0.05 '.' 0.1 ' ' 1
```

```r
# Three-way ANOVA: include additional factor (e.g., Age)
anova_result_3way <- aov(OD ~ MrkA * Biofilm.category * Age , data = bio)
summary(anova_result_3way)
```

```
##                         Df   Sum Sq  Mean Sq F value   Pr(>F)    
## MrkA                     1 0.000337 0.000337   1.116   0.3115    
## Biofilm.category         1 0.017906 0.017906  59.286 5.55e-06 ***
## Age                      1 0.001188 0.001188   3.935   0.0706 .  
## MrkA:Biofilm.category    1 0.001328 0.001328   4.396   0.0579 .  
## MrkA:Age                 1 0.001023 0.001023   3.388   0.0905 .  
## Biofilm.category:Age     1 0.000012 0.000012   0.040   0.8454    
## Residuals               12 0.003624 0.000302                     
## ---
## Signif. codes:  0 '***' 0.001 '**' 0.01 '*' 0.05 '.' 0.1 ' ' 1
```

```r
# Post-hoc comparisons using estimated marginal means
ema <- emmeans(anova_result_3way, pairwise ~ MrkA | Biofilm.category)
summary(ema)
```

```
## $emmeans
## Biofilm.category = Moderate biofilm producer:
##  MrkA      emmean      SE df lower.CL upper.CL
##  Negative   0.162 0.01040 12   0.1391    0.184
##  Positive   0.180 0.00660 12   0.1654    0.194
## 
## Biofilm.category = Weak biofilm producer:
##  MrkA      emmean      SE df lower.CL upper.CL
##  Negative  nonEst      NA NA       NA       NA
##  Positive   0.113 0.00635 12   0.0993    0.127
## 
## Confidence level used: 0.95 
## 
## $contrasts
## Biofilm.category = Moderate biofilm producer:
##  contrast            estimate     SE df t.ratio p.value
##  Negative  - Positive  -0.0181 0.0123 12  -1.471  0.1670
## 
## Biofilm.category = Weak biofilm producer:
##  contrast            estimate     SE df t.ratio p.value
##  Negative  - Positive    nonEst     NA NA      NA      NA
```

```r
# One-way ANOVA with Tukey HSD post-hoc test
q <- aov(bio$OD ~ bio$Biofilm.category)
TukeyHSD(q)
```

```
##   Tukey multiple comparisons of means
##     95% family-wise confidence level
## 
## Fit: aov(formula = bio$OD ~ bio$Biofilm.category)
## 
## $`bio$Biofilm.category`
##                                                          diff          lwr
```

```
## Weak biofilm producer-Moderate biofilm producer -0.06176667 -0.08191911
##                                                         upr    p adj
## Weak biofilm producer-Moderate biofilm producer -0.04161423 5.8e-06
```

Gene Expression Analysis at Different pH Levels

Statistical comparison of Ct values between mrkA-positive and -negative groups under acidic and neutral pH.

```
# Load qPCR gene expression data
gene <- read.csv("Ct_values.csv")

# Group assignment as factor
gene$Group <- as.factor(gene$Group)

# Extract Ct values by mrkA group under pH 5
group_mrkA_positive_acidic <- gene$Ct.Value.pH5[gene$Group == 'mrkA Positive ']
group_mrkA_negative_acidic <- gene$Ct.Value.pH5[gene$Group == 'mrkA Negative ']

# Extract Ct values by mrkA group under pH 7
group_mrkA_positive_normal <- gene$Ct.Value.pH7[gene$Group == 'mrkA Positive ']
group_mrkA_negative_normal <- gene$Ct.Value.pH7[gene$Group == 'mrkA Negative ']

# Median Ct values for each group (acidic condition)
meidan_mpa <- median(sort(group_mrkA_positive_acidic))
meidan_mpn <- median(sort(group_mrkA_negative_acidic))
meidan_mpa
```

```
## [1] -1.18
```

```
meidan_mpn
```

```
## [1] 12.46
```

```
# Mann-Whitney U test for acidic condition
Acidic_mrka <- wilcox.test(group_mrkA_positive_acidic, group_mrkA_negative_acidic, paired = F)
W <- Acidic_mrka$statistic

# Compute effect size (r) for non-parametric comparison
n1 <- length(group_mrkA_positive_acidic)
n2 <- length(group_mrkA_negative_acidic)
mean_U <- n1 * n2 / 2
sd_U <- sqrt(n1 * n2 * (n1 + n2 + 1) / 12)
z <- (W - mean_U) / sd_U
r <- z / sqrt(n1 + n2)
r
```

```
##          W
## -0.2635231
```

```r
# Mann-Whitney U test for normal (pH 7) condition
normal_mrka <- wilcox.test(group_mrkA_positive_normal, group_mrkA_negative_normal, paired = F)
normal_mrka
```

```
##
##  Wilcoxon rank sum exact test
##
## data:  group_mrkA_positive_normal and group_mrkA_negative_normal
## W = 0, p-value = 0.03571
## alternative hypothesis: true location shift is not equal to 0
```

Paired Comparison of Ct Values Under pH 5 vs pH 7

Assess within-subject changes using Wilcoxon signed-rank test.

```r
# Reshape data to long format for paired analysis
long_data <- data.frame(
    value = c(gene$Ct.Value.pH5, gene$Ct.Value.pH7),
    condition = rep(c("pH5", "pH7"), each = length(gene$Ct.Value.pH5)),
    subject = rep(1:length(gene$Ct.Value.pH5), 2)
)

# Convert variables to factors
long_data$subject <- as.factor(long_data$subject)
long_data$condition <- as.factor(long_data$condition)

head(long_data, 5)
```

```
##   value condition subject
## 1 -2.72       pH5       1
## 2 -1.18       pH5       2
## 3  1.06       pH5       3
## 4 -2.55       pH5       4
## 5  2.41       pH5       5
```

```r
# Wilcoxon signed-rank test (paired data)
wilcox_test(value ~ condition | subject, data = long_data)
```

```
##
##  Asymptotic Wilcoxon-Mann-Whitney Test
##
## data:  value by
##   condition (pH5, pH7)
##   stratified by subject
## Z = -2.0926, p-value = 0.03639
## alternative hypothesis: true mu is not equal to 0
```

```r
# Effect size (r) calculation for paired test
z <- -2.0926
n <- length(unique(long_data$subject))
r <- z / sqrt(n)
r
```

ID	Group	Gene	delta delta Ct value	Fold.change	Conditions	delta Ct value
29	mrkA Positive	TEM	-2.37	5.2	pH7	-2.72
29	mrkA Positive	TEM	-2.37	5.2	pH5	-0.35
29	mrkA Positive	mrkA	-1.60	3.0	pH7	-1.18
29	mrkA Positive	mrkA	-1.60	3.0	pH5	0.42
37	mrkA Positive	mrkA	-2.37	5.2	pH7	1.06
37	mrkA Positive	mrkA	-2.37	5.2	pH5	3.43

```
## [1] -0.7398458
```

Gene Expression delta Ct Value Visualization

Transform the dataset to long format and visualize delta Ct values under different pH conditions.

```r
# Rename columns for clarity
colnames(gene) <- c('ID','Group','Gene','pH7','pH5','delta delta Ct value','Fold.change')

# Reshape wide-format gene expression data into long format
gene_long <- gene %>%
        pivot_longer(
                cols = starts_with('pH7') | starts_with('pH5'),
                names_to = 'Conditions',
                values_to = 'delta Ct value'
        )

# Preview reshaped data
head(gene_long) %>% gt()
```

```r
gene_long<- as.data.frame(gene_long)
qqnorm(gene_long[,c('delta Ct value')], main = 'Q-Q plot of delta Ct values',
       cex.lab = 1.5, cex.axis = 1.5, pch = 16, cex.main = 2, font.lab= 2,cex = 2)
qqline(gene_long[,c('delta Ct value')], col = 'red',lwd = 5)
```

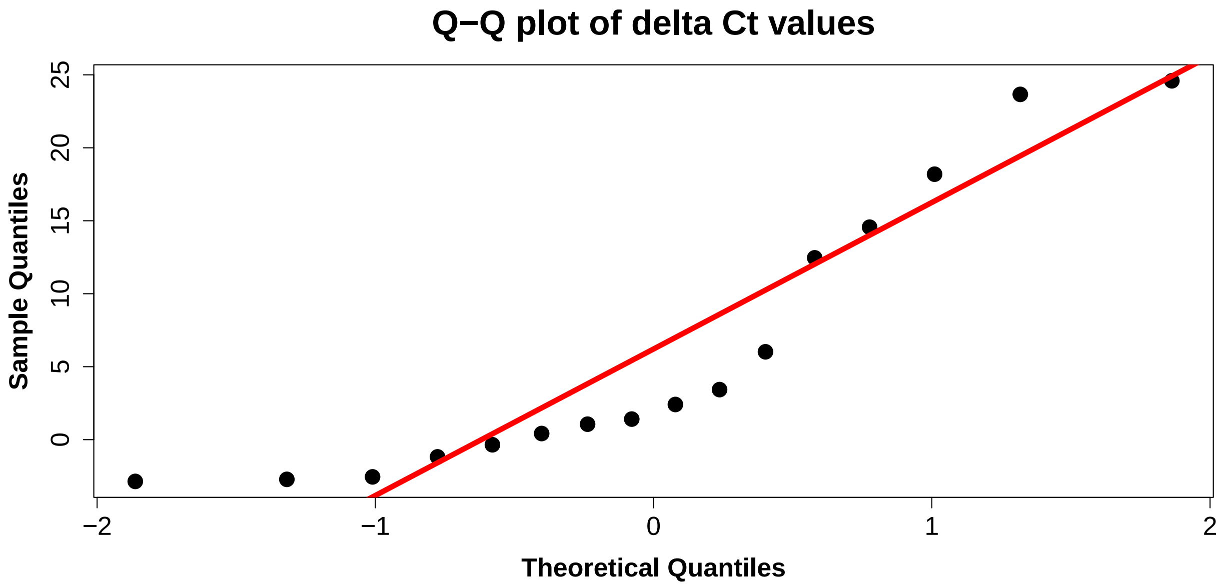


```
# Generate violin and boxplot for delta Ct values under different environmental conditions
plt <- ggbetweenstats(data = gene_long, x = Conditions, y = `delta Ct value`, palette = 'RdBu', type =
plt
```

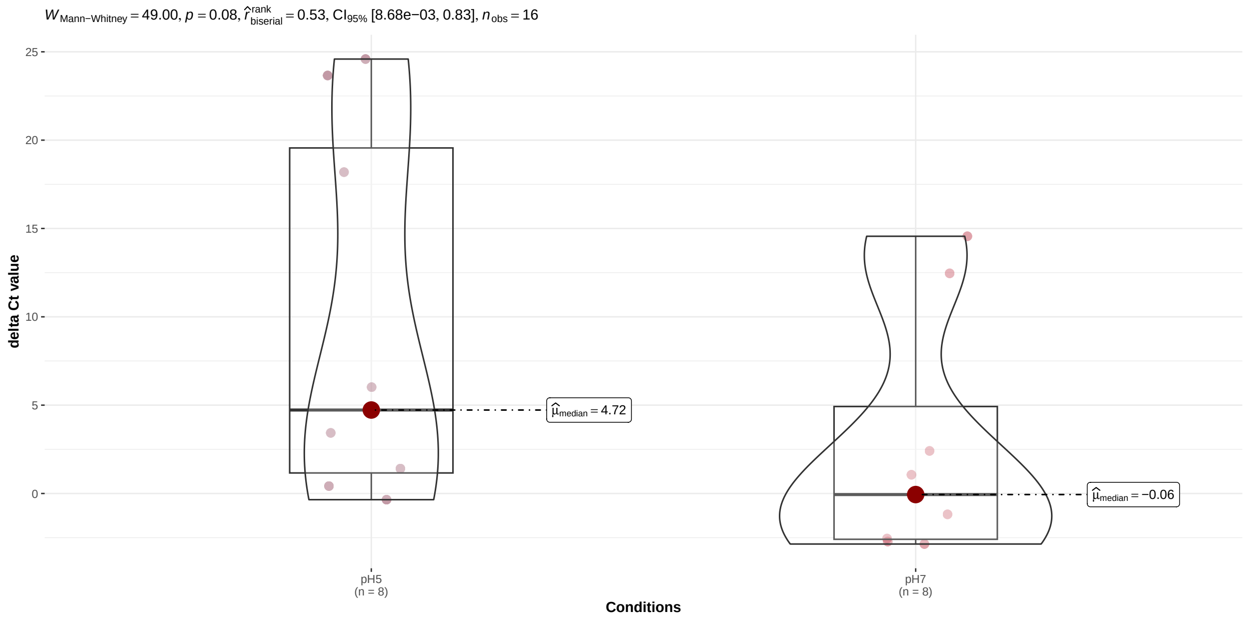

Binary Encoding of Gene Markers

Create binary variables (0/1) for presence of resistance or biofilm-related genes for downstream analysis.

```
# Encode gene presence as binary variables
all$mrkA <- ifelse(all$MrkA == 'Positive ', 1, 0)
all$blaSHV <- ifelse(all$SHV == 'Positive', 1, 0)
all$blaTEM <- ifelse(all$TEM == 'Positive ', 1, 0)
```

Numeric Data Extraction for Correlation Analysis

Prepare numeric subset for computing correlations among antibiotic resistance, gene presence, and phenotypic traits.

```
# Select numeric columns from the dataset
num_all <- all %>%
    select(where(is.numeric))

# Rename columns for more readable labels in the heatmap
colnames(num_all) <- c(
    "Age",
    "Optical Density",
    "Cefepime MIC",
    "Ceftriaxone MIC",
    "Meropenem MIC",
    "Imipenem MIC",
    "Amikacin MIC",
    "Gentamicin MIC",
    "Ciprofloxacin MIC",
    "Levofloxacin MIC",
    "Colistin MIC",
    "mrkA ",
    "blaSHV",
    "blaTEM"
)
```

Correlation Heatmap

Visualize correlations between MIC values, optical density, age, and gene markers using a heatmap.

```
# Compute correlation matrix
cor_data <- cor(num_all)
# Generate annotated heatmap of correlation matrix
pheatmap(
    cor_data,
    display_numbers = TRUE,
    border_color = 'black',
    color = colorRampPalette(c('lightblue','coral','white'))(100),
    fontsize = 12,
    fontsize_number = 12,
    angle_col = 45
)
```

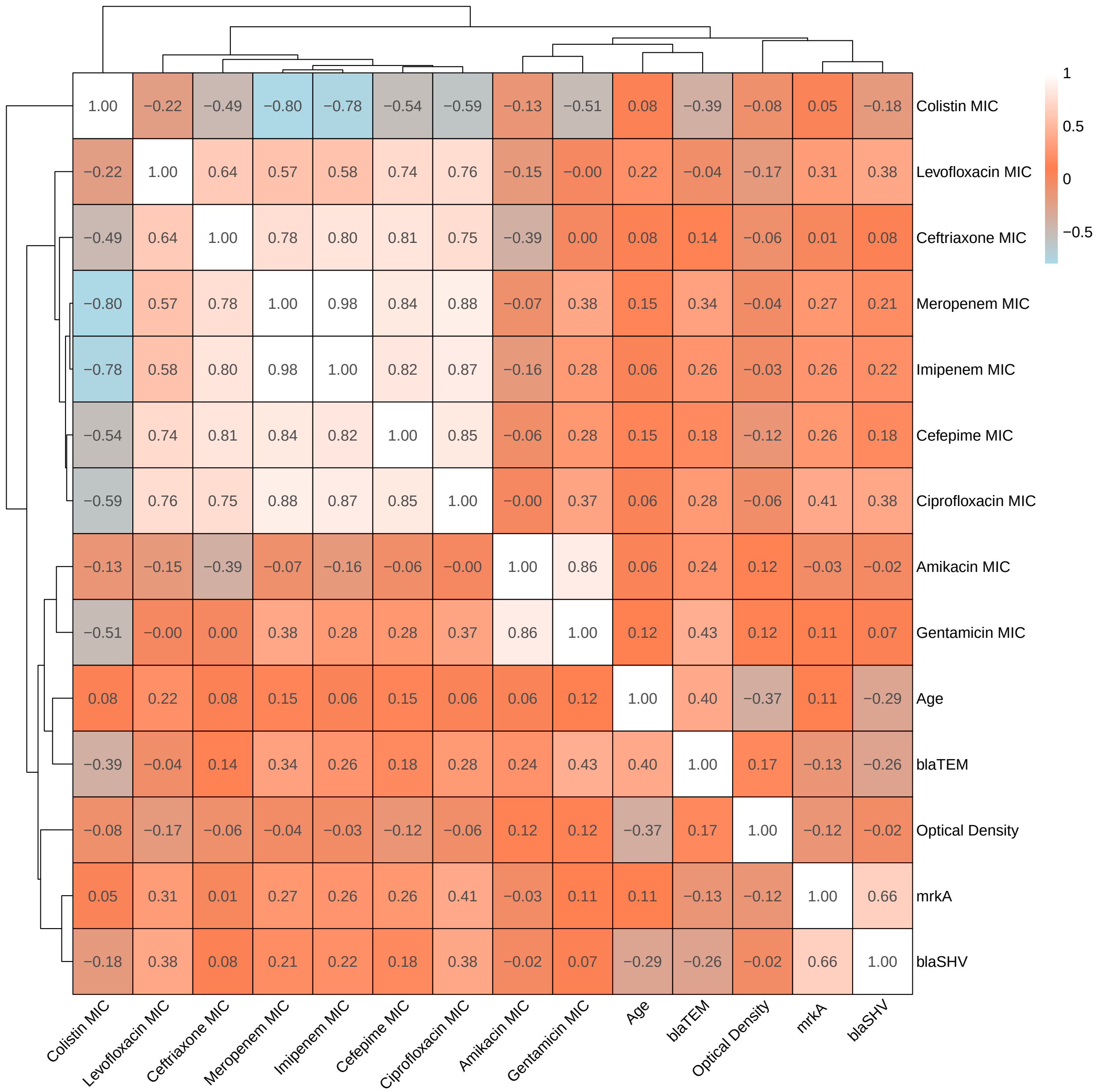
Colistin MIC
Levofloxacin MIC
Ceftriaxone MIC
Meropenem MIC
Imipenem MIC
Cefepime MIC
Ciprofloxacin MIC
Amikacin MIC
Gentamicin MIC
Age
blaTEM
Optical Density
mrkA
blaSHV
1
0.5
0
−0.5
