# Supplementary material for: Exploring the Relationship Between Biofilm Formation and Antibiotic Resistance Genes in Clinically Isolated Klebsiella pneumoniae
Source: Int J Microbiol. 2025 Oct 16;2025:3833882. doi: 10.1155/ijm/3833882 (PMC12549196; doi:10.1155/ijm/3833882)
Supplement: Supporting Information — Additional supporting information can be found online in the Supporting Information section. The following supporting information is provided to support the findings and reproducibility of this research. (1) README: instructions on how to use the data and run the analysis code. (2) Folder1_Data: contains the raw data in CSV format. (3) Folder2_Scipts: contains the code for the analysis. (4) Folder3_Outputs: figures and plot generated for the study. [file 3833882.f1.zip › Data-analysis/Folder1_Data/Raw_gel_images.pdf]

mrkA gene

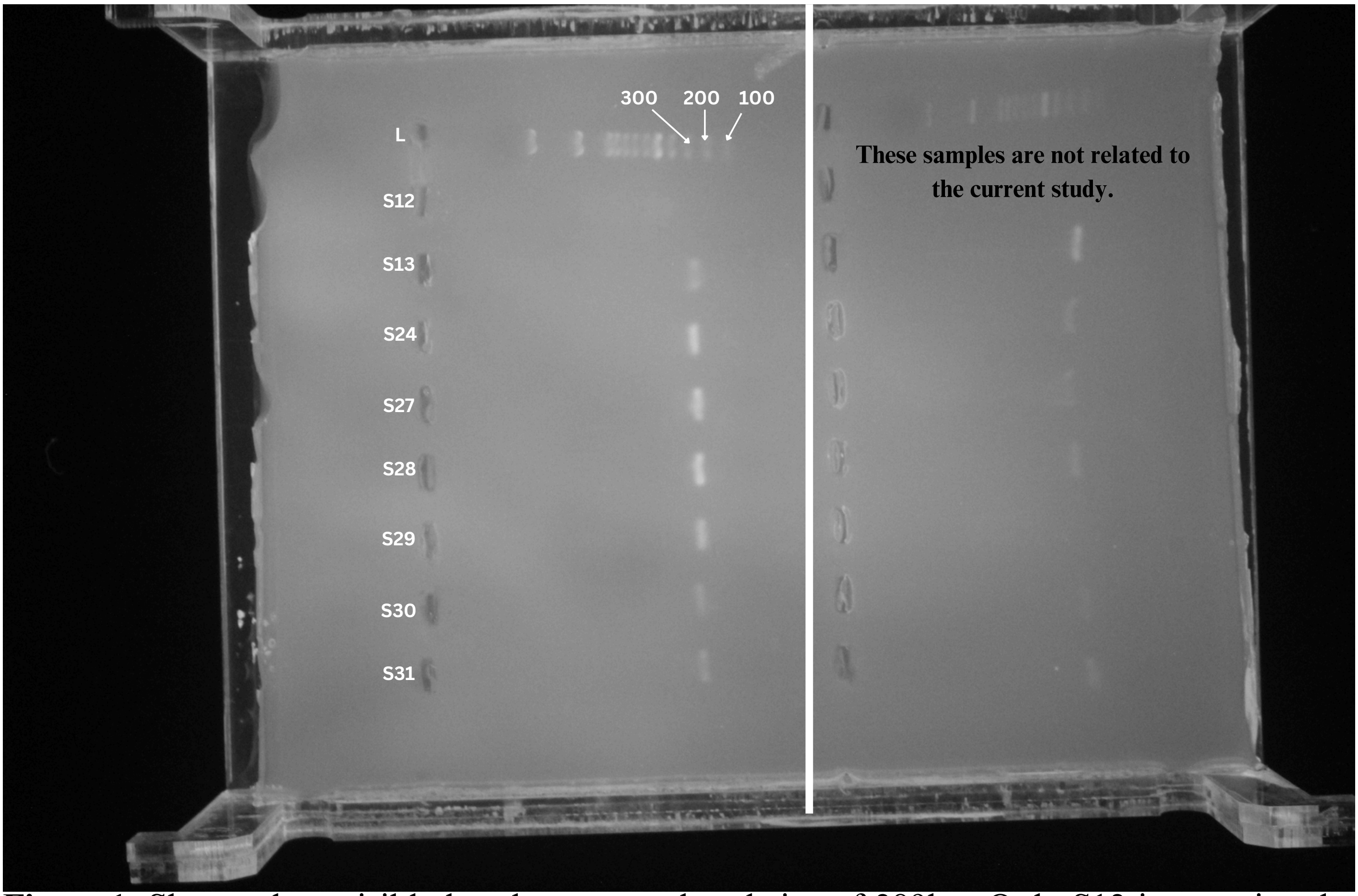

Figure 1: Shows clear visible band at expect band size of 289bp. Only S12 is negative the rest of the samples are positive. The bands on the right side are not related to this study.

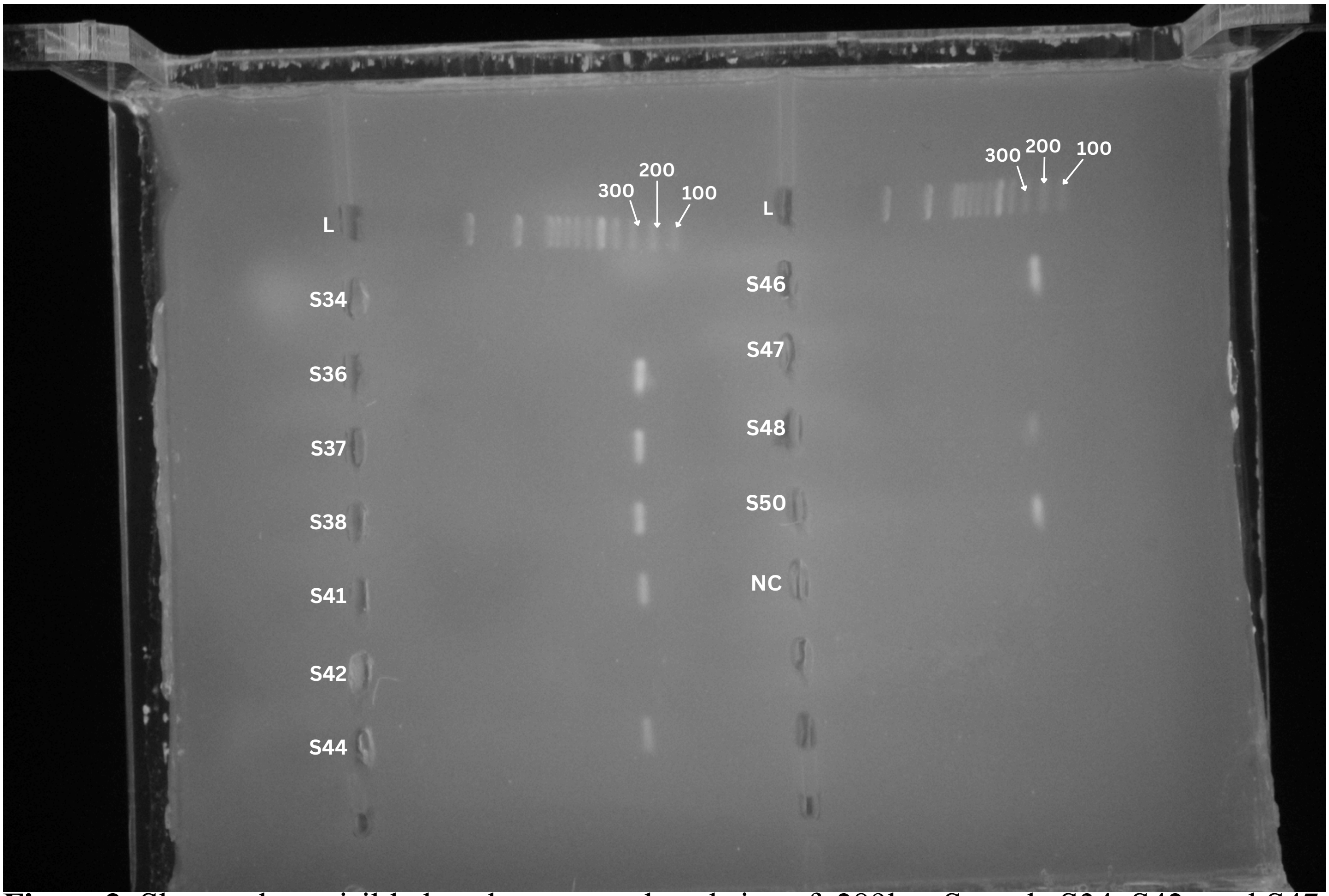

Figure 2: Shows clear visible band at expect band size of 289bp. Sample S34, S42, and S47 are negative for mrkA genes, the rest of samples are positive. Negative control is shown as NC

blaSHV

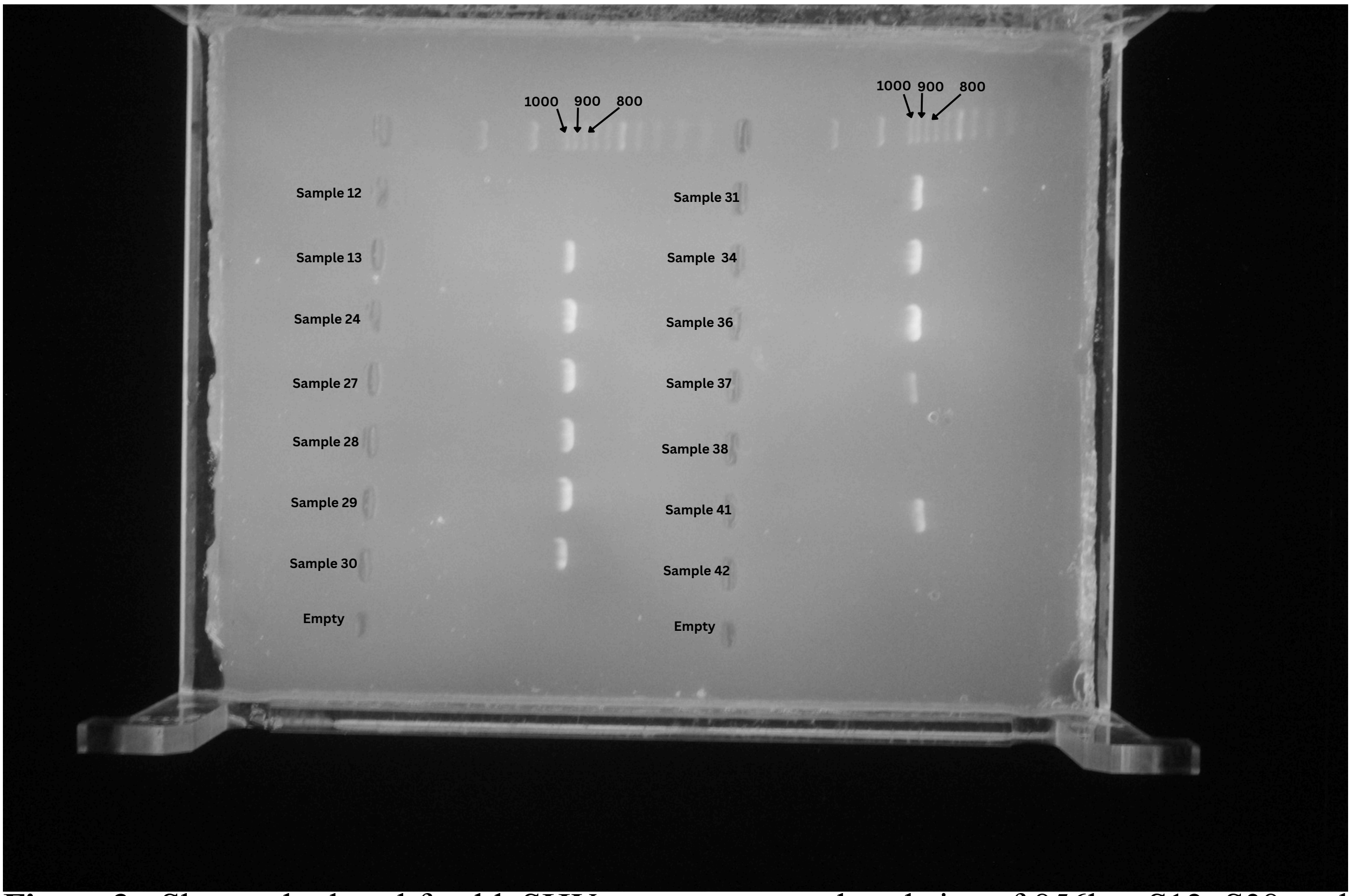

Figure 3: Shows the band for blaSHV gene at expect band size of 856bp. S12, S38 and S42 are negative, the rest of samples are positive for blaSHV gene. Due to small capacity of the last well, it was left unused.

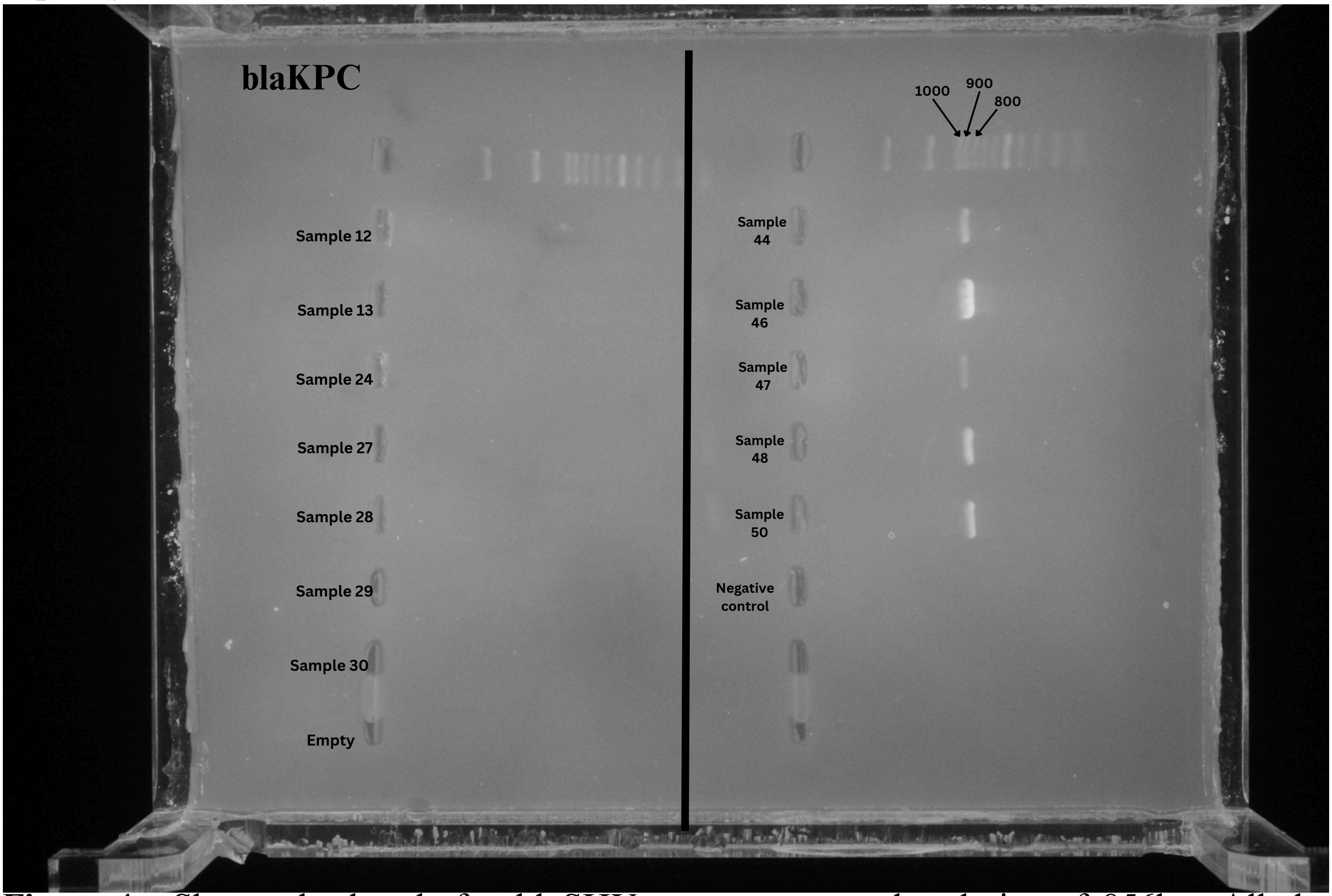

Figure 4: Shows the bands for blaSHV gene at expect band size of 856bp. All the samples in this gel are positive for the gene. Due to small capacity of the last well, it was left unused. The left side of the gel was used for blaKPC genes, as shown all samples tested negative. The rest of the samples for blaKPC are shown in the next page

blaKPC

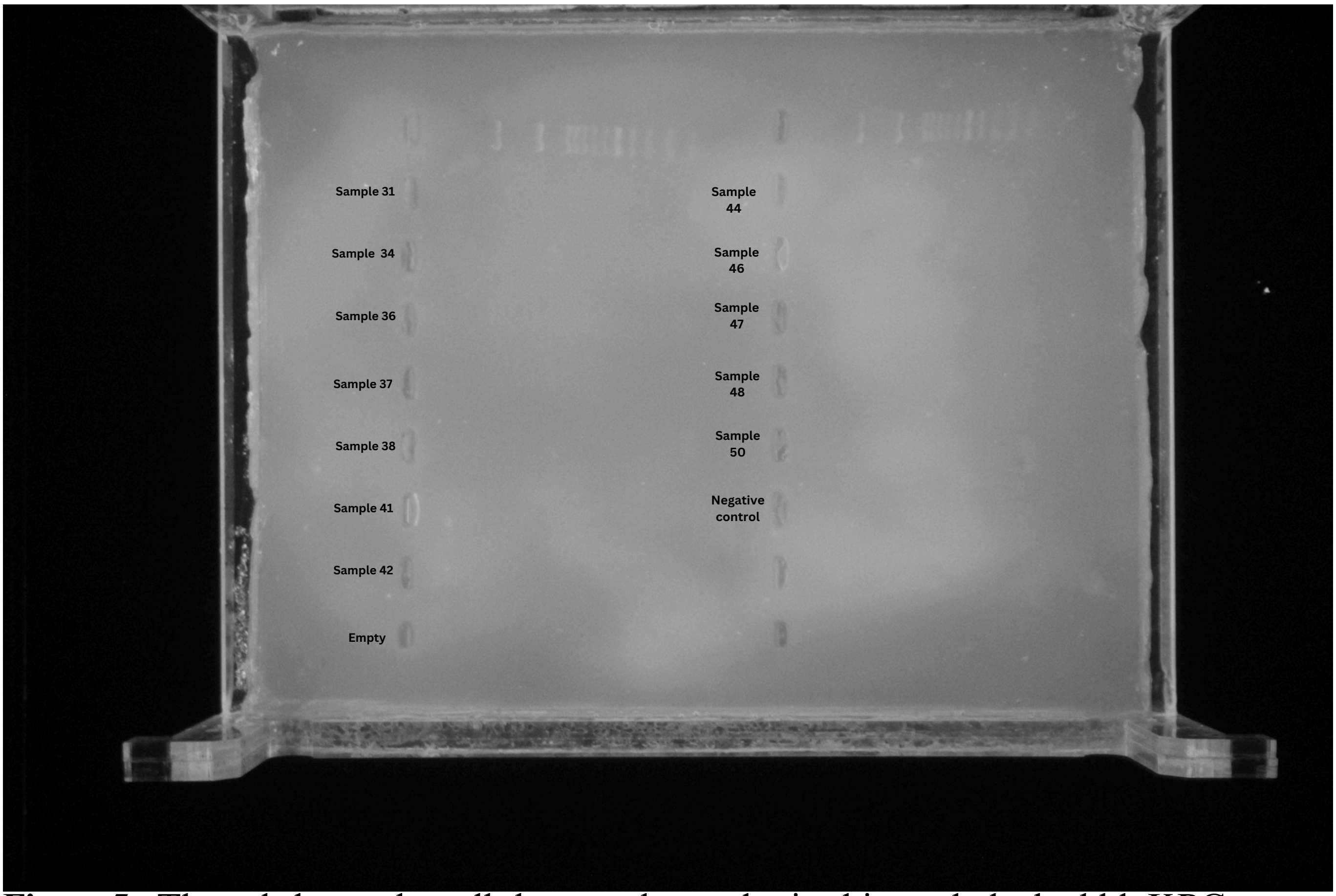

Figure 5: The gel shows that all the tested samples in this study lacked blaKPC genes. As shown there are no visible bands for any of the samples.

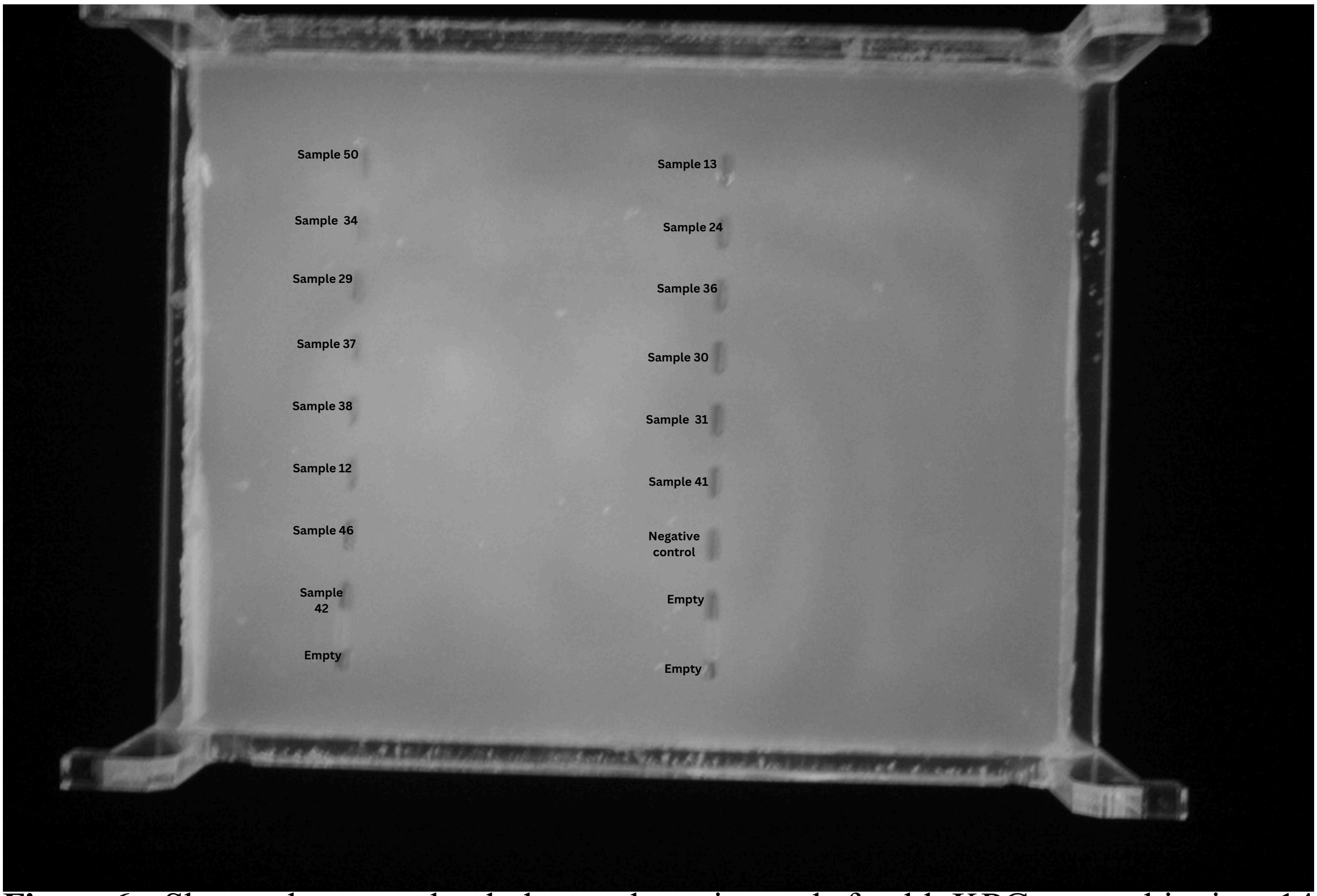

Figure 6: Shows the second gel electrophoresis result for blaKPC gene, this time 14 samples were chosen randomly to be tested again for the presence of blaKPC gene. As shown in the figure all sample again tested negative.

blaTEM

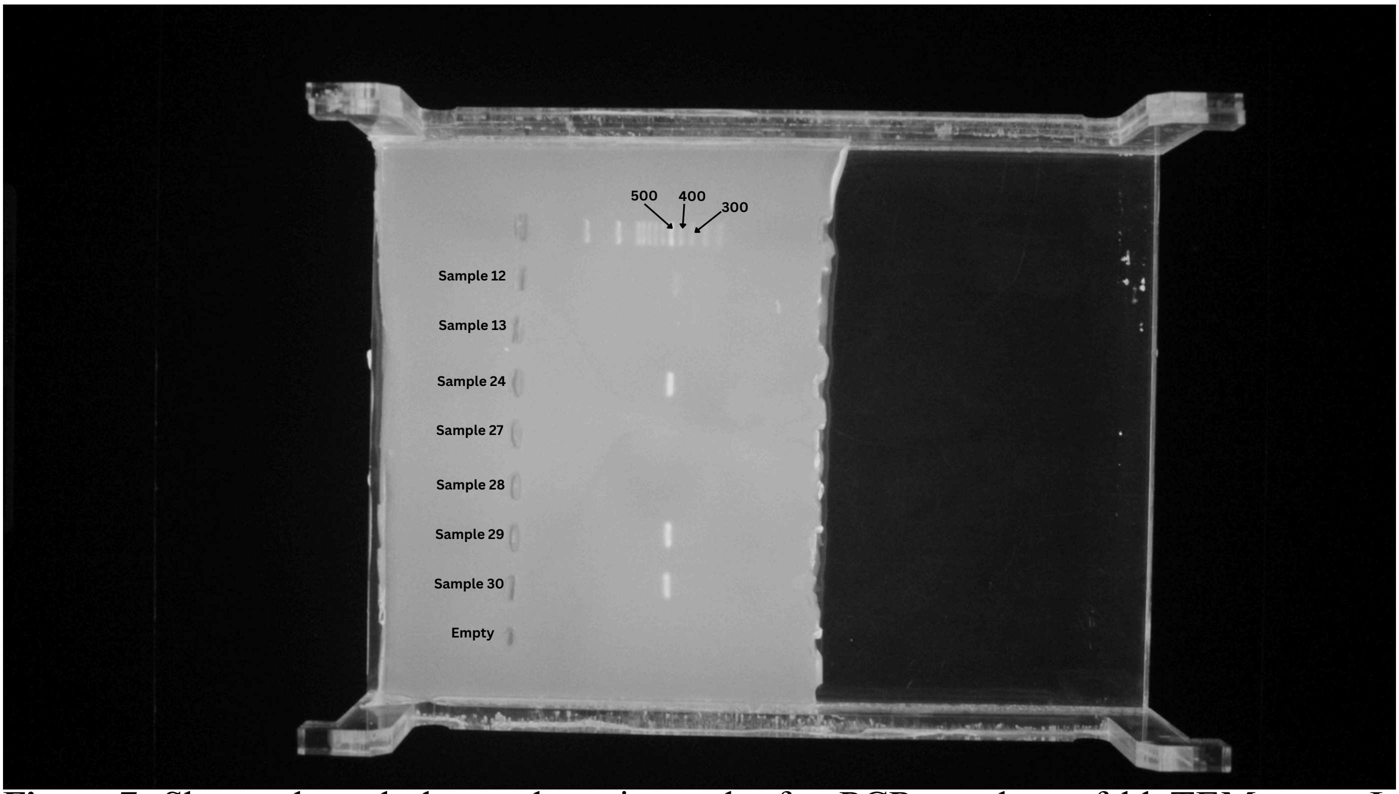

Figure 7: Shows the gel electrophoresis results for PCR product of blaTEM gene. It shows that sample 12,27, and 28 are negative for blaTEM genes, while the rest of samples are positive. The bands are at the expected size of 403 bp

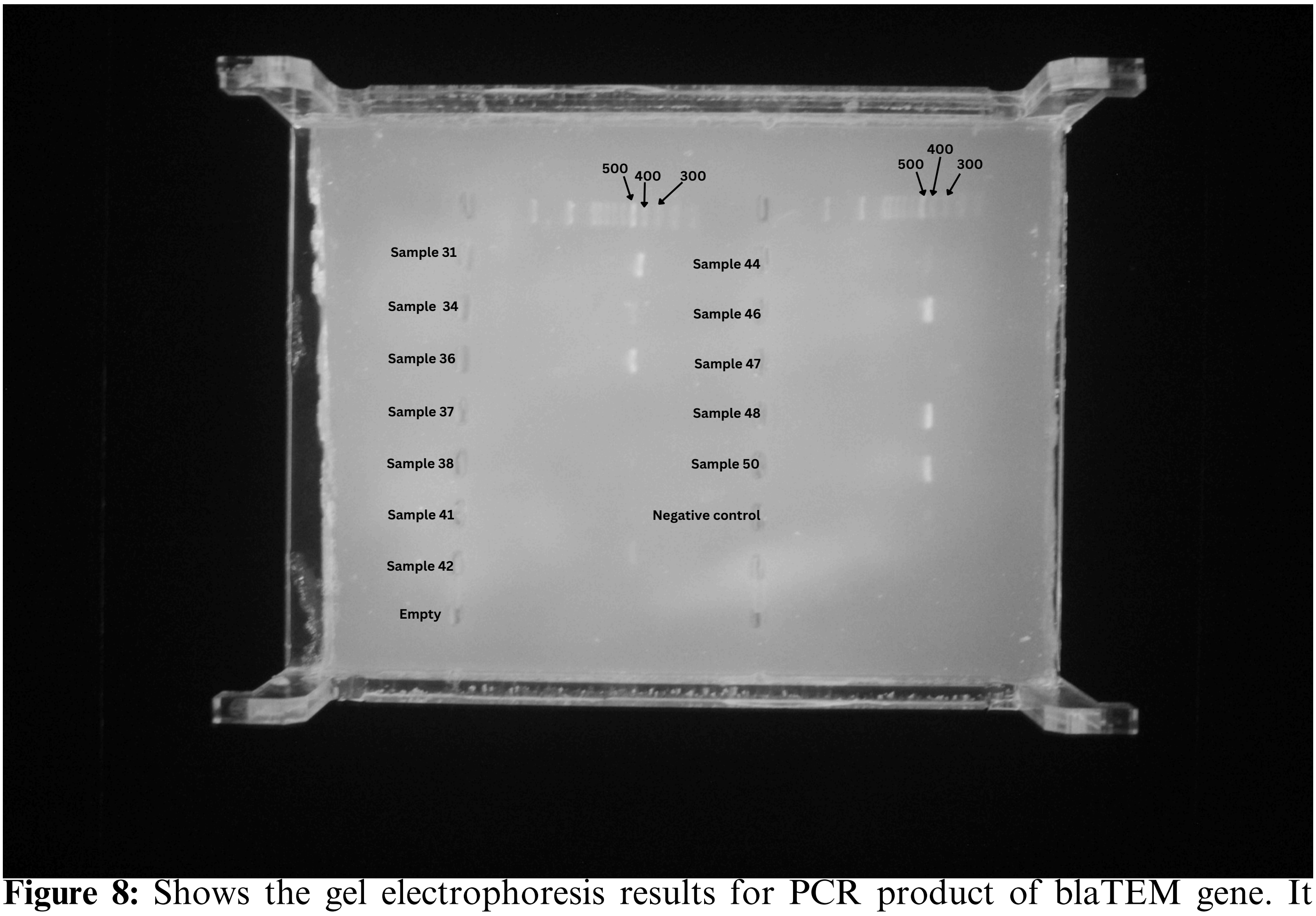

Figure 8: Shows the gel electrophoresis results for PCR product of blaTEM gene. It shows that sample 37, 38, 41, and 47 are negative for blaTEM genes, while the rest of samples are positive. The bands are at the expected size of 403 bp

16s rRNA

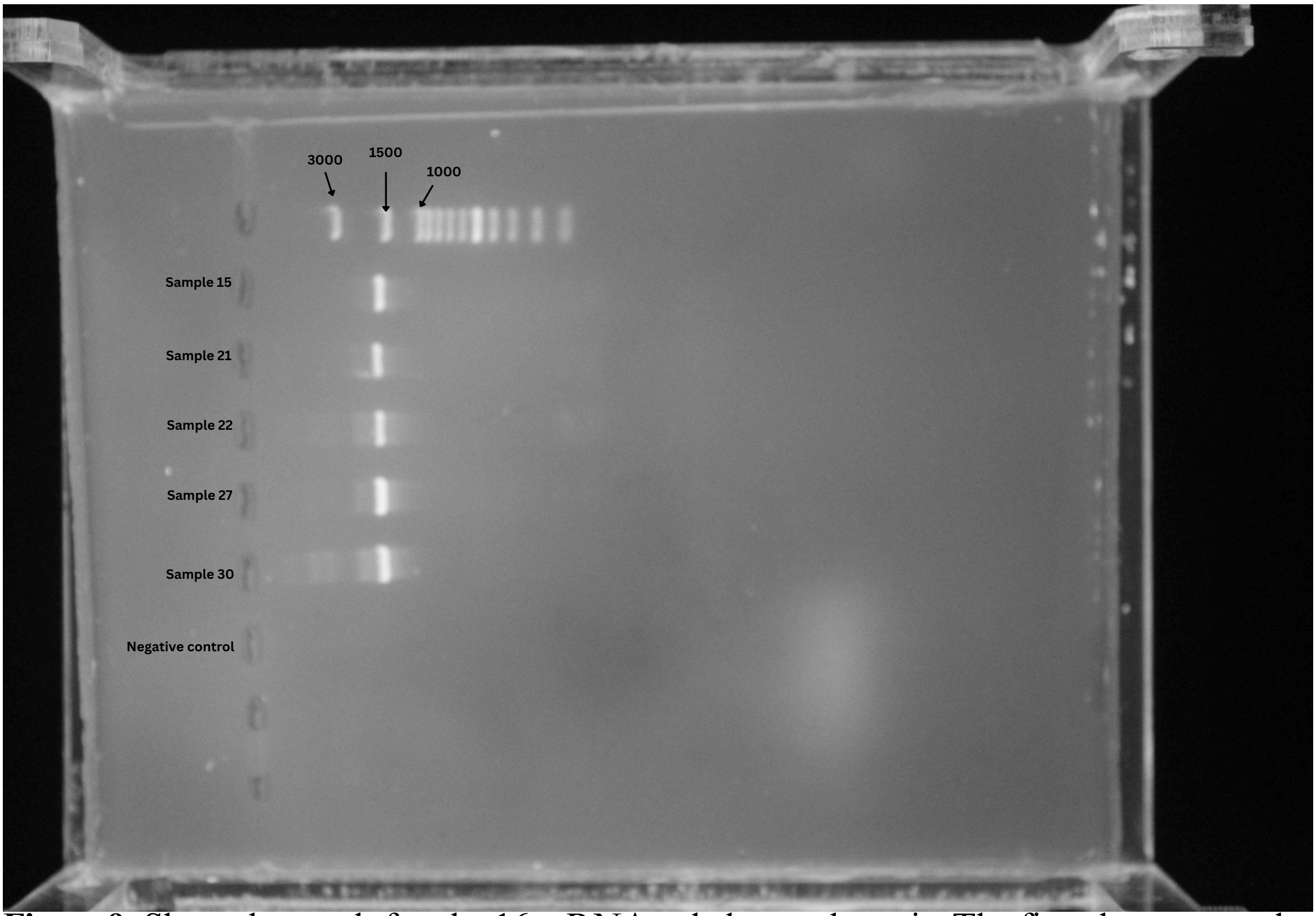

Figure 9: Show the result for the 16s rRNA gel electrophoresis. The five chosen samples all show clear visible bands at the expect band size of 1500bp.
