# Supplementary figures and images for: Exploring the Relationship Between Biofilm Formation and Antibiotic Resistance Genes in Clinically Isolated Klebsiella pneumoniae
Source: Int J Microbiol. 2025 Oct 16;2025:3833882. doi: 10.1155/ijm/3833882 (PMC12549196; doi:10.1155/ijm/3833882)

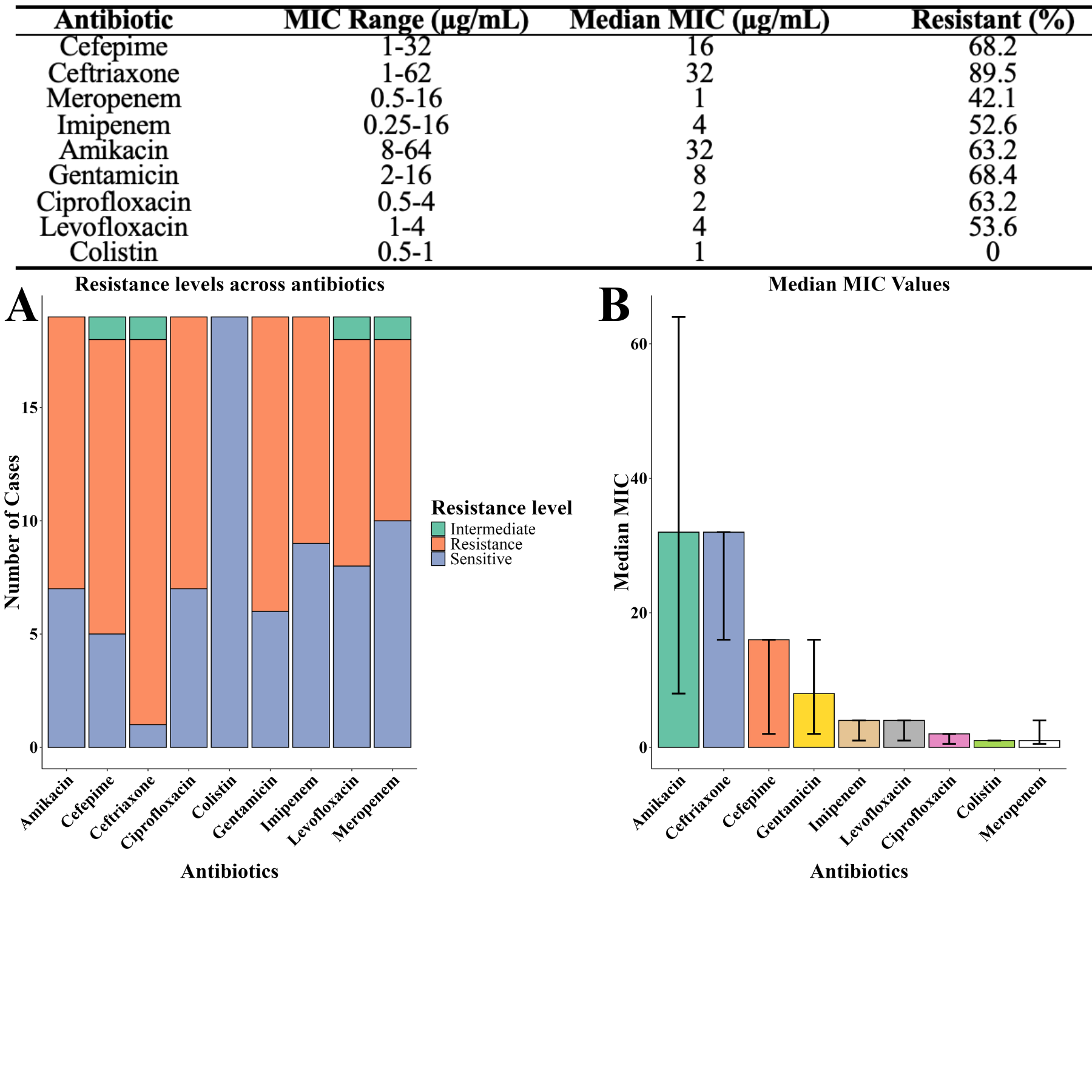

Supplement: Supporting Information — Additional supporting information can be found online in the Supporting Information section. The following supporting information is provided to support the findings and reproducibility of this research. (1) README: instructions on how to use the data and run the analysis code. (2) Folder1_Data: contains the raw data in CSV format. (3) Folder2_Scipts: contains the code for the analysis. (4) Folder3_Outputs: figures and plot generated for the study. [file 3833882.f1.zip › Data-analysis/Folder3_Outputs/Resistance_levels.png.png]

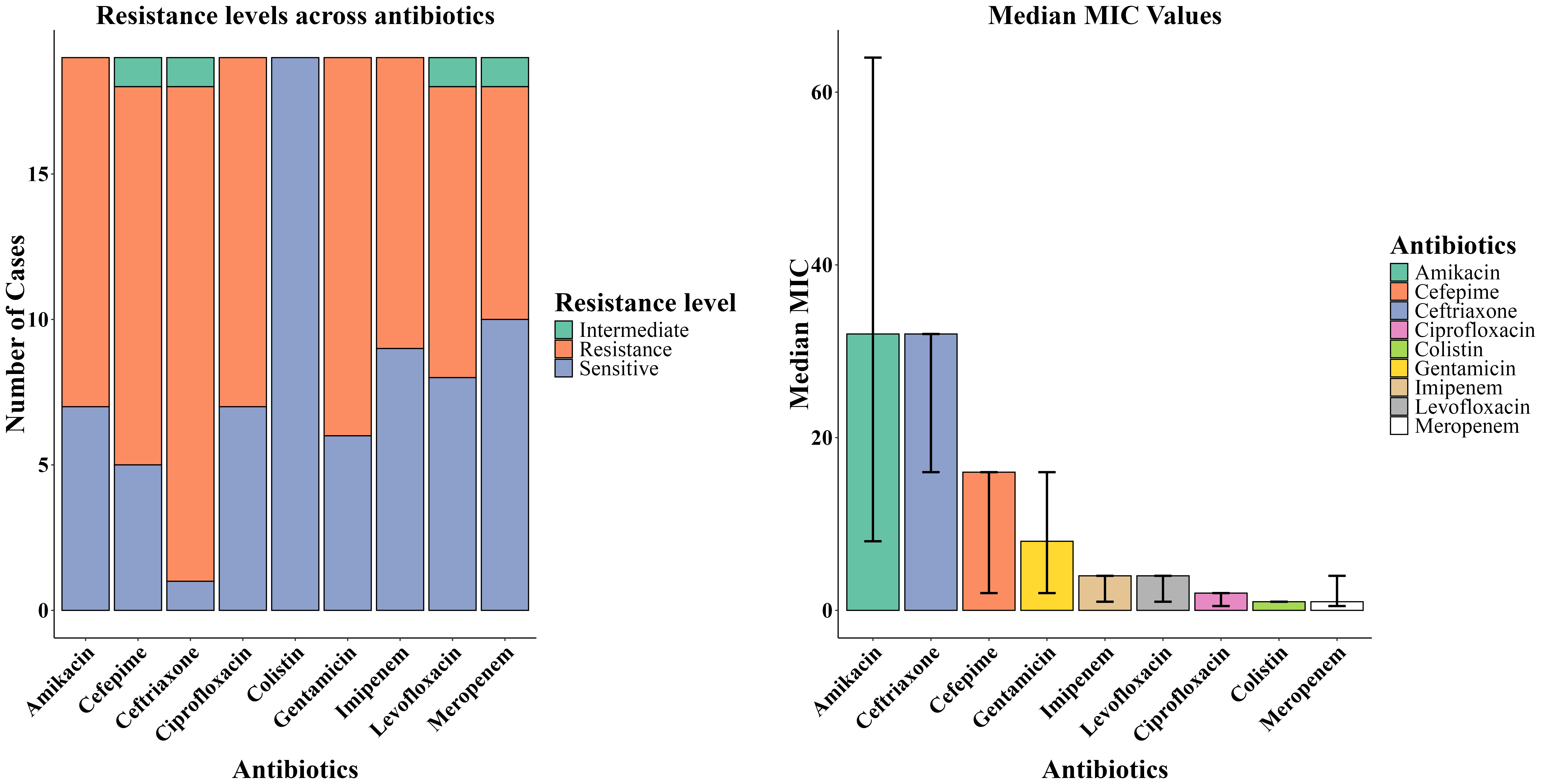

Supplement: Supporting Information — Additional supporting information can be found online in the Supporting Information section. The following supporting information is provided to support the findings and reproducibility of this research. (1) README: instructions on how to use the data and run the analysis code. (2) Folder1_Data: contains the raw data in CSV format. (3) Folder2_Scipts: contains the code for the analysis. (4) Folder3_Outputs: figures and plot generated for the study. [file 3833882.f1.zip › Data-analysis/Folder3_Outputs/gridplot_resistance_levels.png]

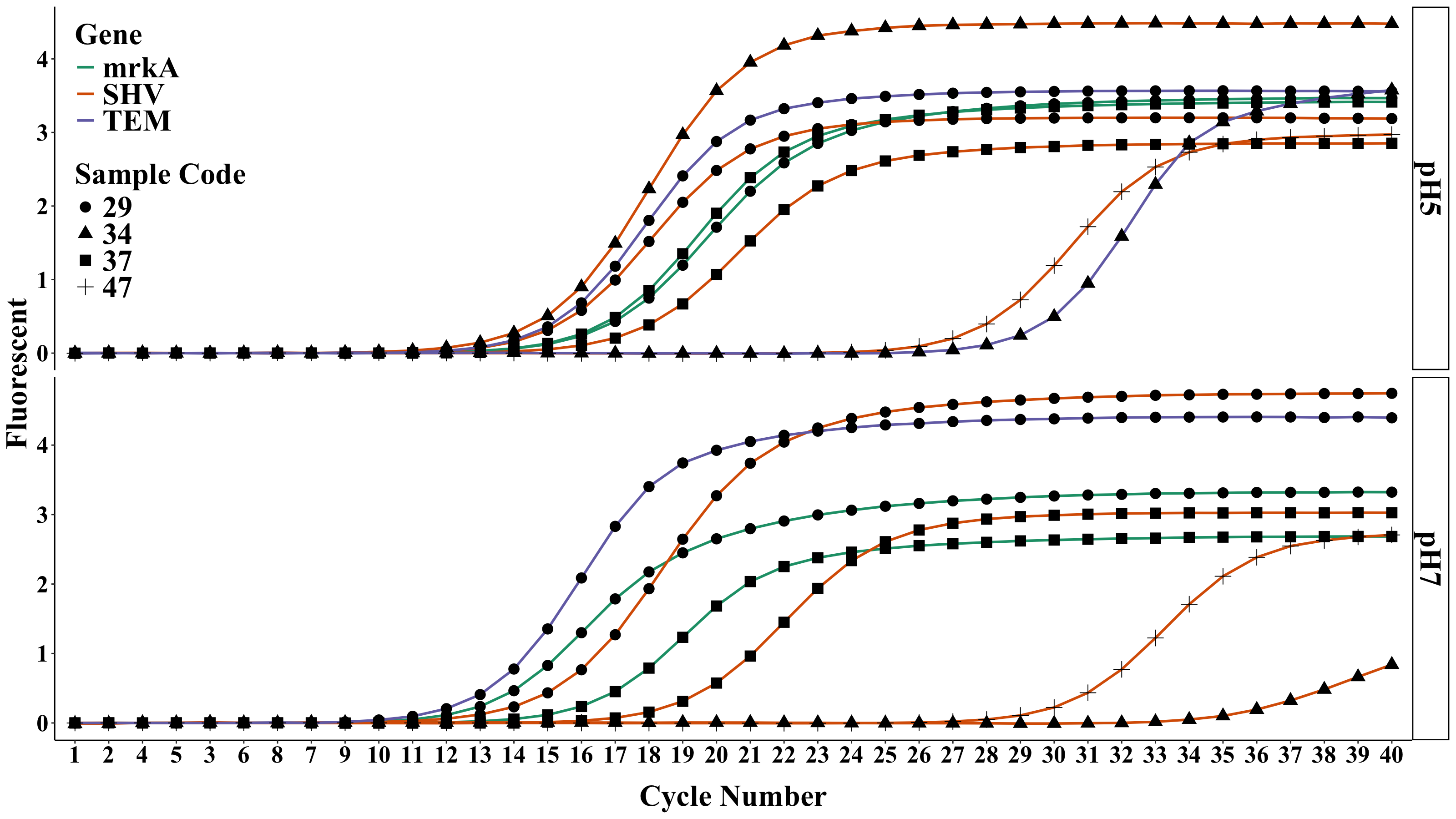

Supplement: Supporting Information — Additional supporting information can be found online in the Supporting Information section. The following supporting information is provided to support the findings and reproducibility of this research. (1) README: instructions on how to use the data and run the analysis code. (2) Folder1_Data: contains the raw data in CSV format. (3) Folder2_Scipts: contains the code for the analysis. (4) Folder3_Outputs: figures and plot generated for the study. [file 3833882.f1.zip › Data-analysis/Folder3_Outputs/RT-qPCR.png]

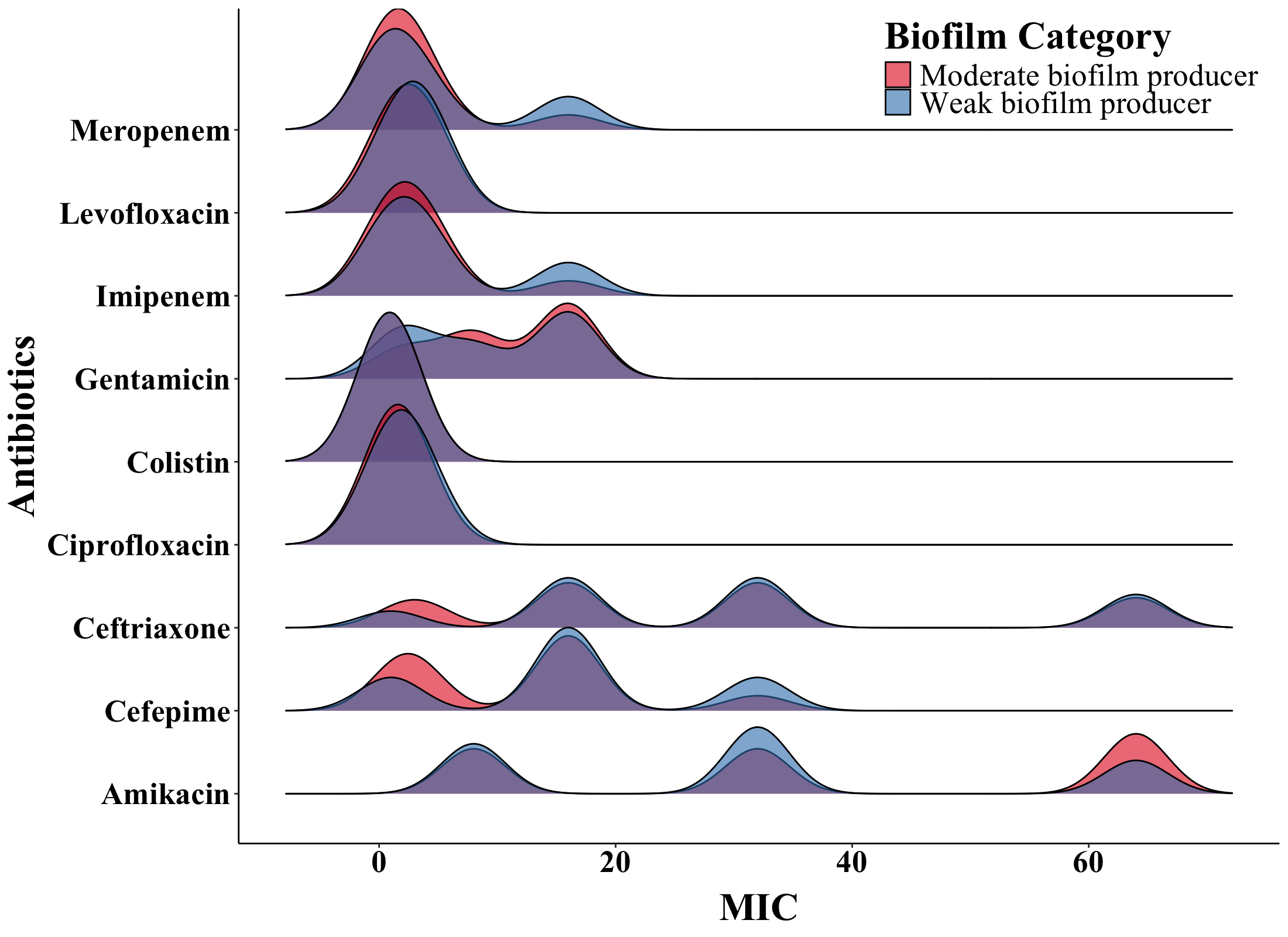

Supplement: Supporting Information — Additional supporting information can be found online in the Supporting Information section. The following supporting information is provided to support the findings and reproducibility of this research. (1) README: instructions on how to use the data and run the analysis code. (2) Folder1_Data: contains the raw data in CSV format. (3) Folder2_Scipts: contains the code for the analysis. (4) Folder3_Outputs: figures and plot generated for the study. [file 3833882.f1.zip › Data-analysis/Folder3_Outputs/density_ridges.png]

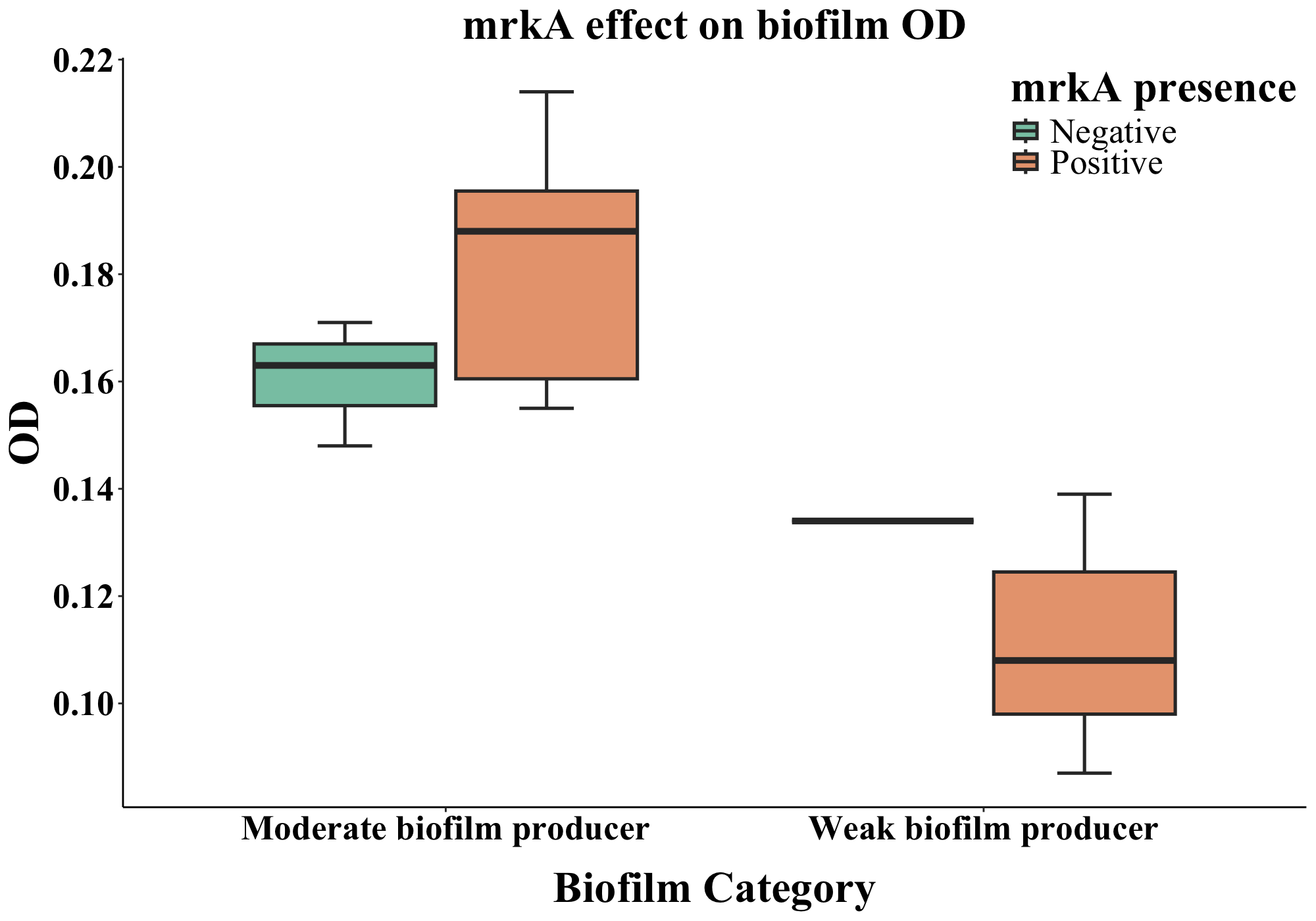

Supplement: Supporting Information — Additional supporting information can be found online in the Supporting Information section. The following supporting information is provided to support the findings and reproducibility of this research. (1) README: instructions on how to use the data and run the analysis code. (2) Folder1_Data: contains the raw data in CSV format. (3) Folder2_Scipts: contains the code for the analysis. (4) Folder3_Outputs: figures and plot generated for the study. [file 3833882.f1.zip › Data-analysis/Folder3_Outputs/boxplot mrkA.png]

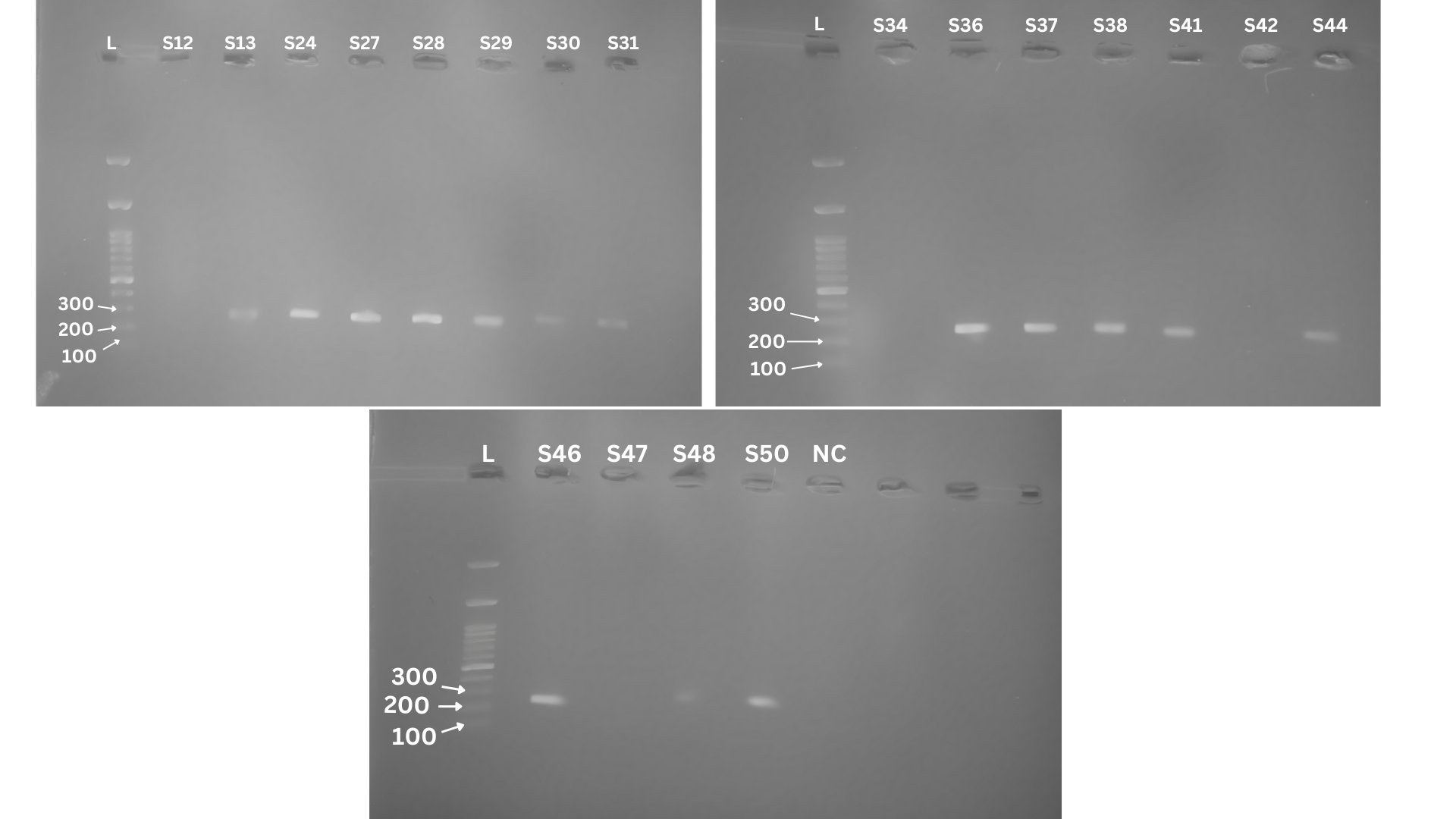

Supplement: Supporting Information — Additional supporting information can be found online in the Supporting Information section. The following supporting information is provided to support the findings and reproducibility of this research. (1) README: instructions on how to use the data and run the analysis code. (2) Folder1_Data: contains the raw data in CSV format. (3) Folder2_Scipts: contains the code for the analysis. (4) Folder3_Outputs: figures and plot generated for the study. [file 3833882.f1.zip › Data-analysis/Folder3_Outputs/mrkA_three_images.png]

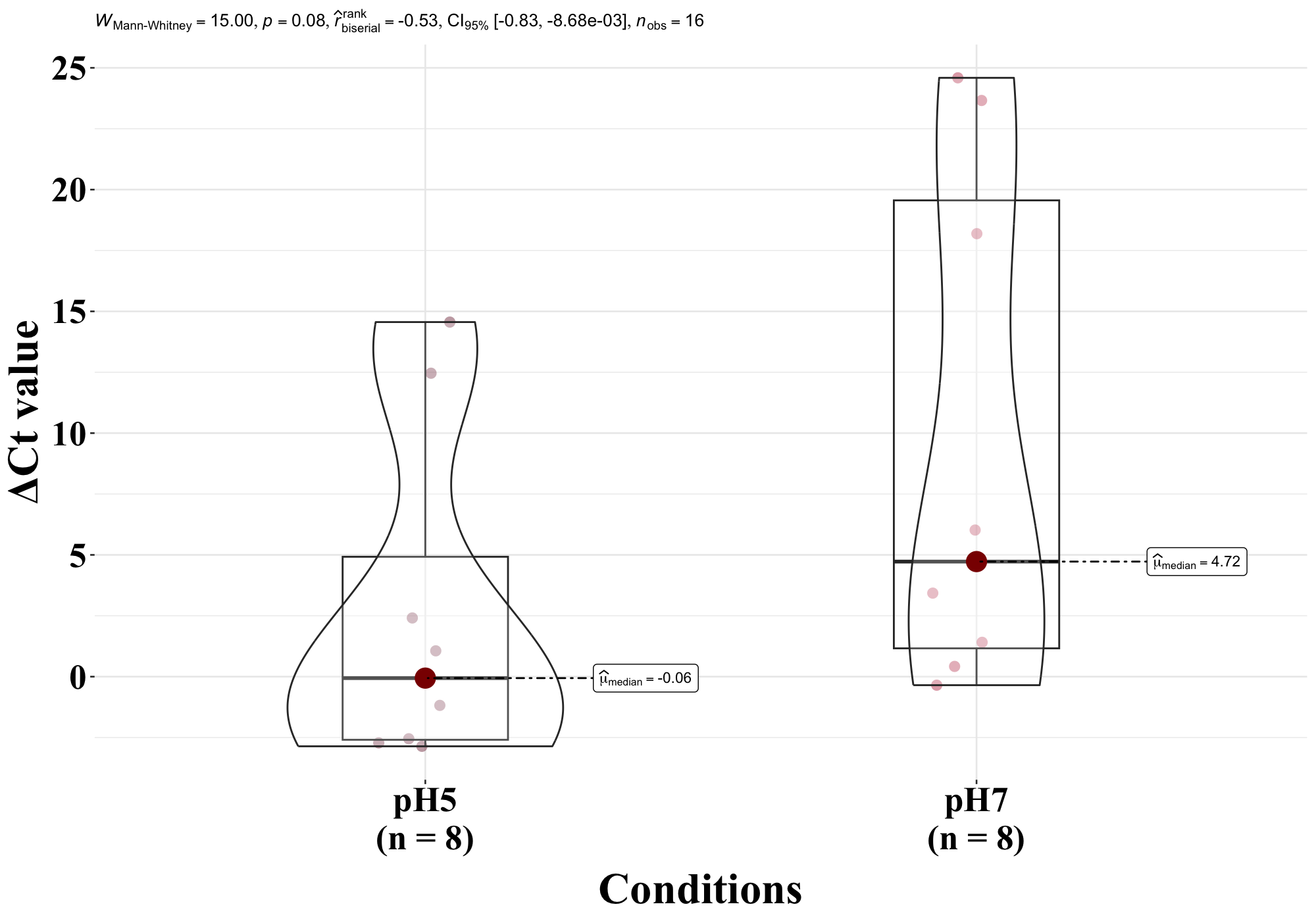

Supplement: Supporting Information — Additional supporting information can be found online in the Supporting Information section. The following supporting information is provided to support the findings and reproducibility of this research. (1) README: instructions on how to use the data and run the analysis code. (2) Folder1_Data: contains the raw data in CSV format. (3) Folder2_Scipts: contains the code for the analysis. (4) Folder3_Outputs: figures and plot generated for the study. [file 3833882.f1.zip › Data-analysis/Folder3_Outputs/box_violion.png]

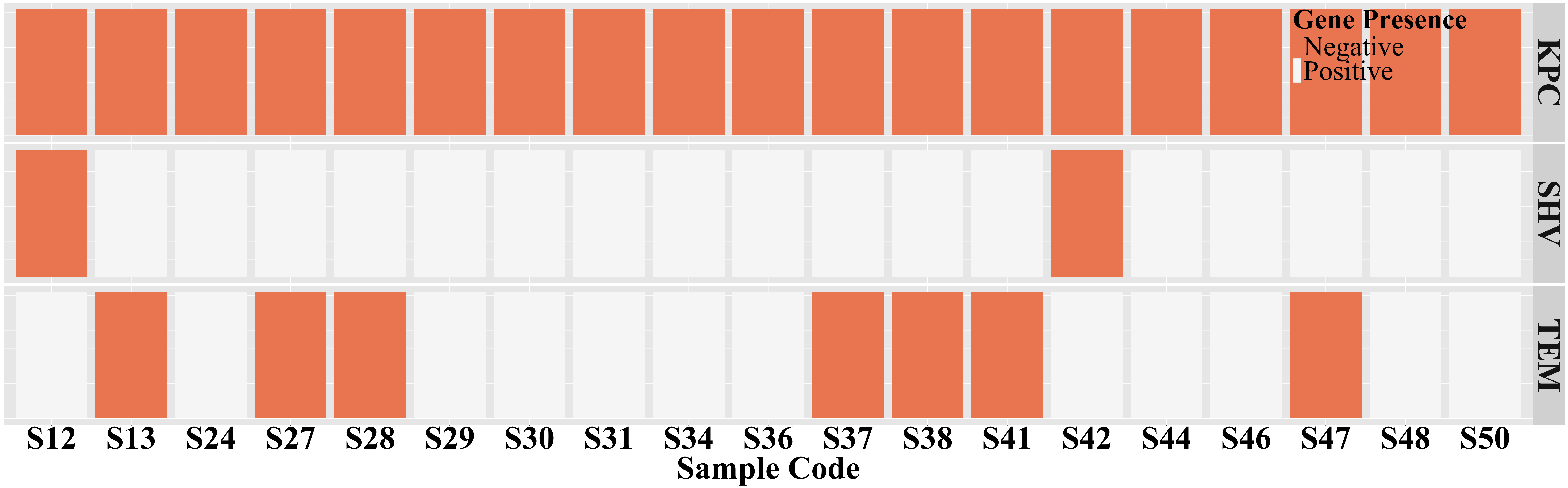

Supplement: Supporting Information — Additional supporting information can be found online in the Supporting Information section. The following supporting information is provided to support the findings and reproducibility of this research. (1) README: instructions on how to use the data and run the analysis code. (2) Folder1_Data: contains the raw data in CSV format. (3) Folder2_Scipts: contains the code for the analysis. (4) Folder3_Outputs: figures and plot generated for the study. [file 3833882.f1.zip › Data-analysis/Folder3_Outputs/Bars for gene presence.png]

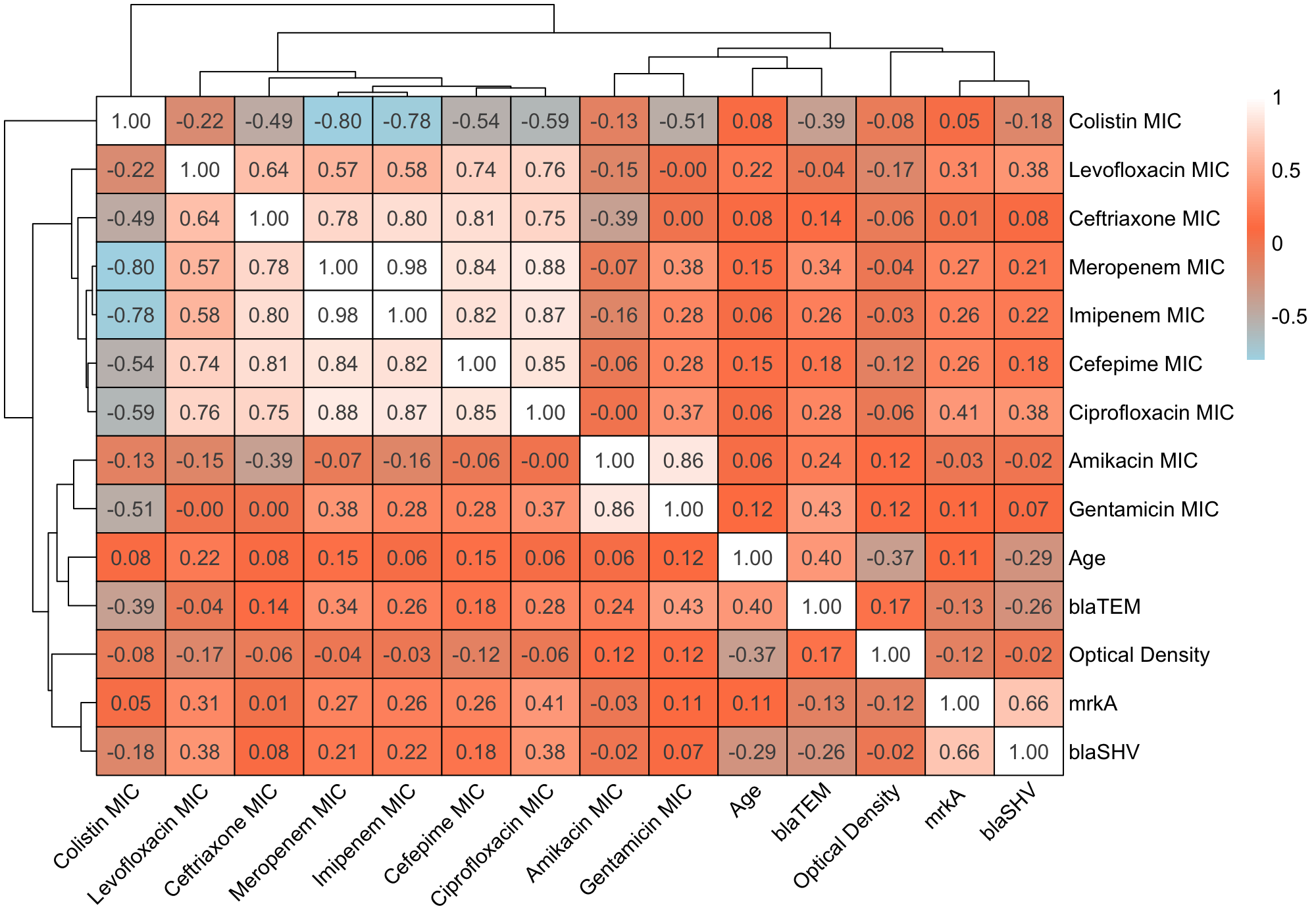

Supplement: Supporting Information — Additional supporting information can be found online in the Supporting Information section. The following supporting information is provided to support the findings and reproducibility of this research. (1) README: instructions on how to use the data and run the analysis code. (2) Folder1_Data: contains the raw data in CSV format. (3) Folder2_Scipts: contains the code for the analysis. (4) Folder3_Outputs: figures and plot generated for the study. [file 3833882.f1.zip › Data-analysis/Folder3_Outputs/matrix.png]

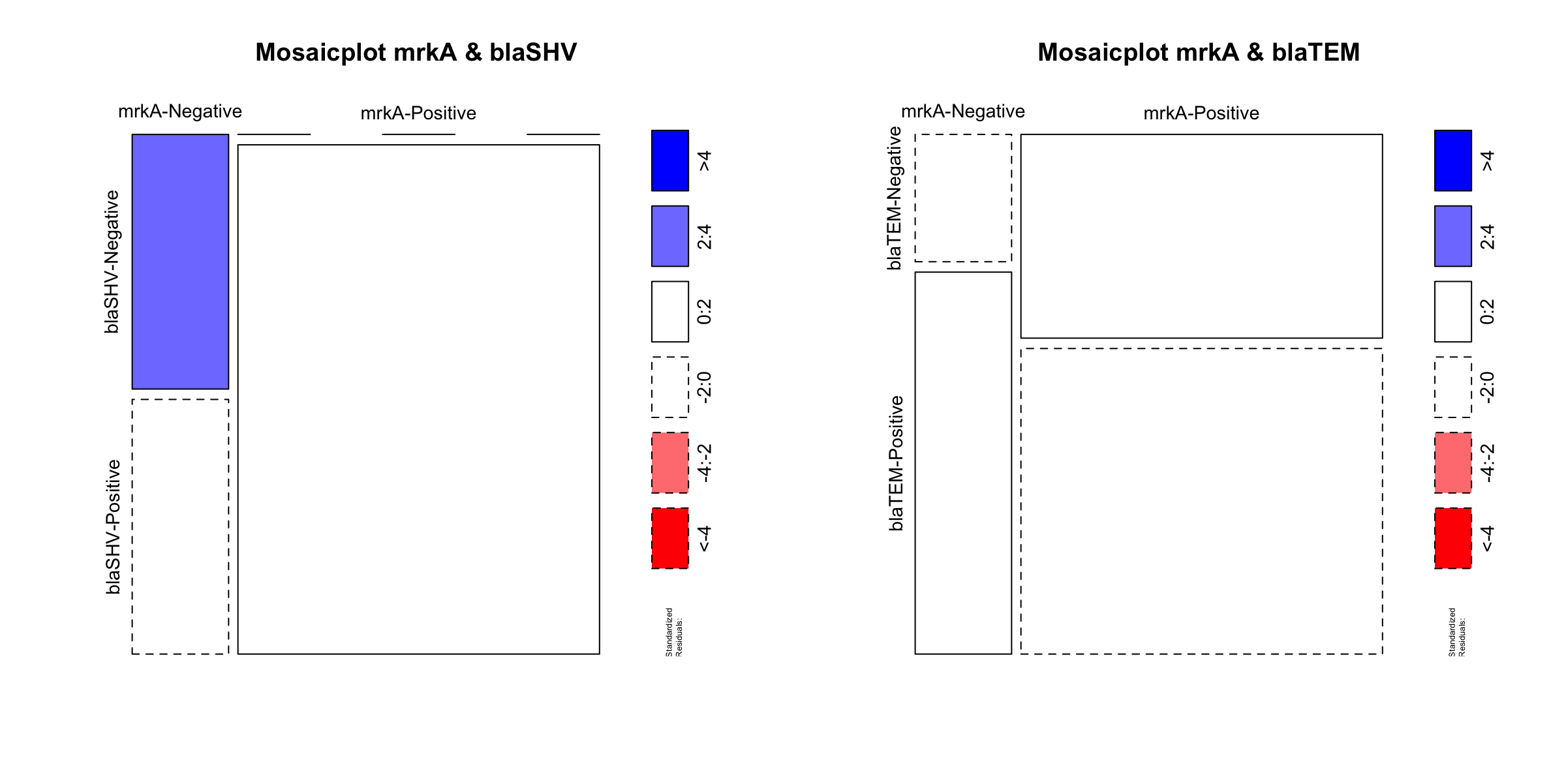

Supplement: Supporting Information — Additional supporting information can be found online in the Supporting Information section. The following supporting information is provided to support the findings and reproducibility of this research. (1) README: instructions on how to use the data and run the analysis code. (2) Folder1_Data: contains the raw data in CSV format. (3) Folder2_Scipts: contains the code for the analysis. (4) Folder3_Outputs: figures and plot generated for the study. [file 3833882.f1.zip › Data-analysis/Folder3_Outputs/moasicplot.png]

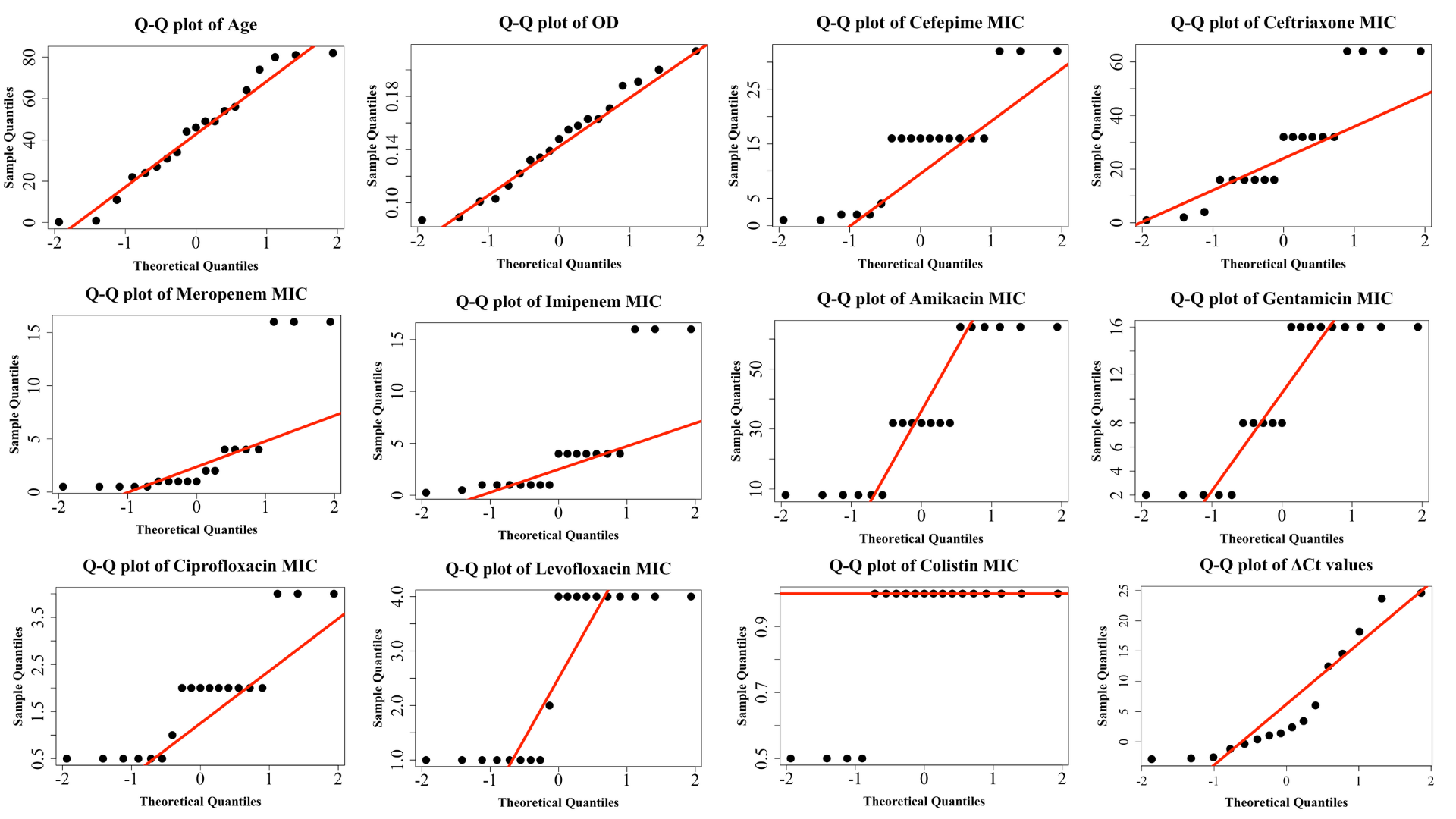

Supplement: Supporting Information — Additional supporting information can be found online in the Supporting Information section. The following supporting information is provided to support the findings and reproducibility of this research. (1) README: instructions on how to use the data and run the analysis code. (2) Folder1_Data: contains the raw data in CSV format. (3) Folder2_Scipts: contains the code for the analysis. (4) Folder3_Outputs: figures and plot generated for the study. [file 3833882.f1.zip › Data-analysis/Folder3_Outputs/Q-Qplots.png]

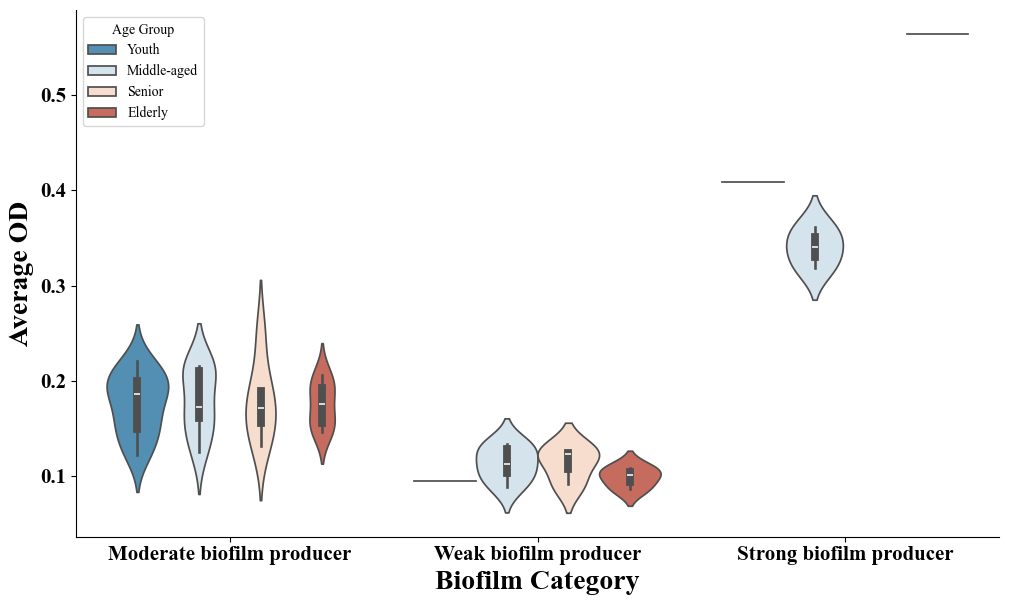

Supplement: Supporting Information — Additional supporting information can be found online in the Supporting Information section. The following supporting information is provided to support the findings and reproducibility of this research. (1) README: instructions on how to use the data and run the analysis code. (2) Folder1_Data: contains the raw data in CSV format. (3) Folder2_Scipts: contains the code for the analysis. (4) Folder3_Outputs: figures and plot generated for the study. [file 3833882.f1.zip › Data-analysis/Folder3_Outputs/Biofilm_age_distribution.png]

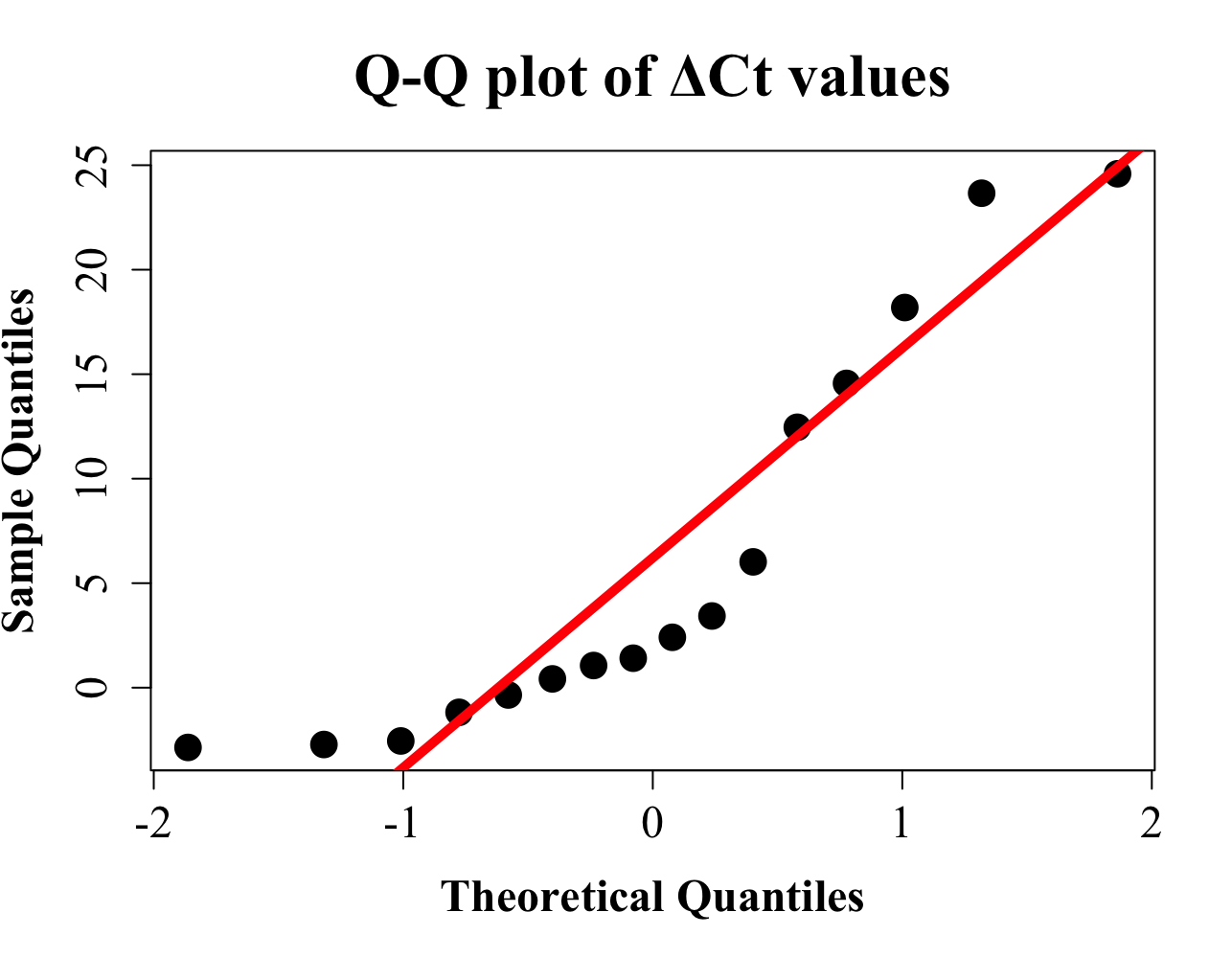

Supplement: Supporting Information — Additional supporting information can be found online in the Supporting Information section. The following supporting information is provided to support the findings and reproducibility of this research. (1) README: instructions on how to use the data and run the analysis code. (2) Folder1_Data: contains the raw data in CSV format. (3) Folder2_Scipts: contains the code for the analysis. (4) Folder3_Outputs: figures and plot generated for the study. [file 3833882.f1.zip › Data-analysis/Folder3_Outputs/Individual Q-Qplot/Q-Q12.png]

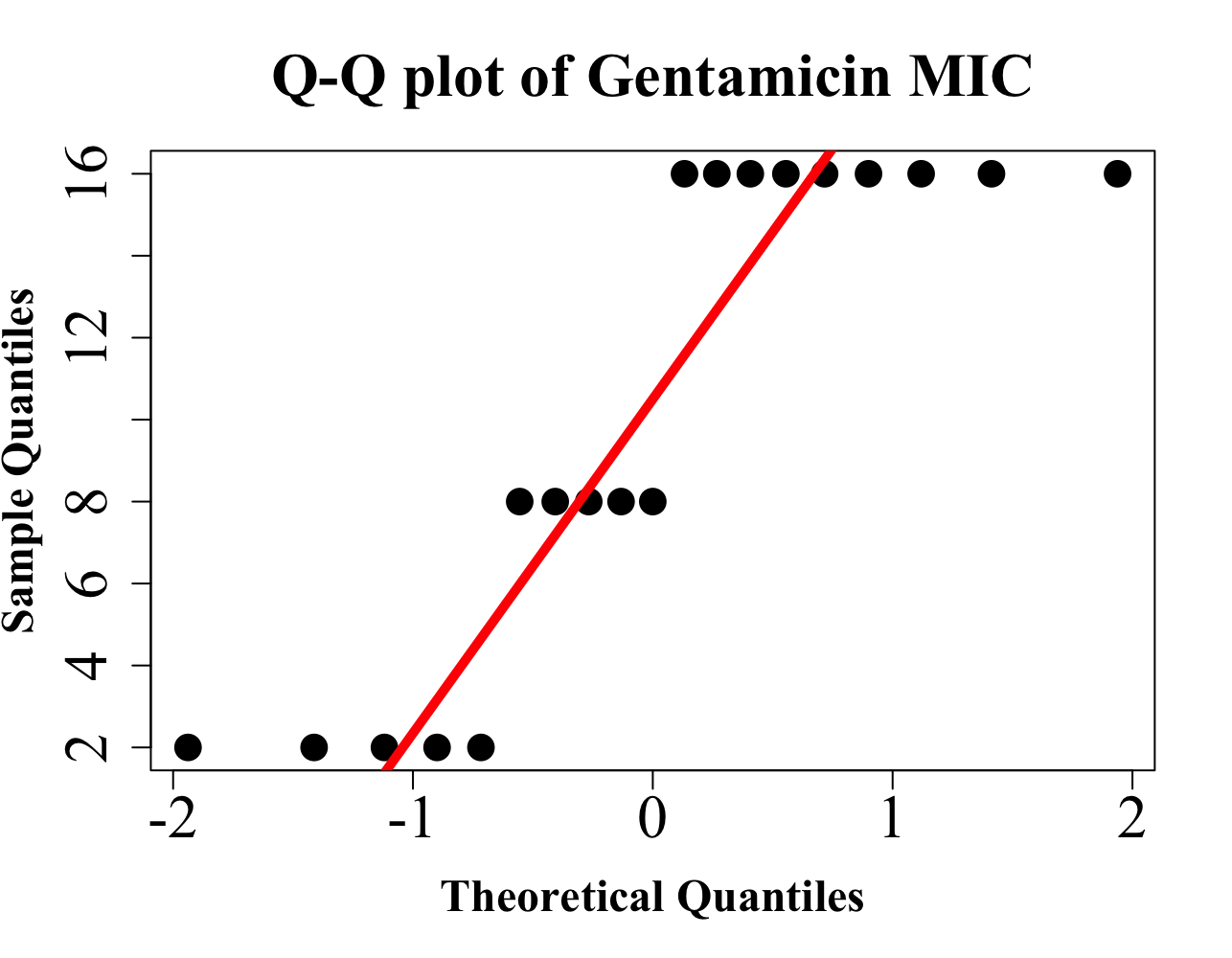

Supplement: Supporting Information — Additional supporting information can be found online in the Supporting Information section. The following supporting information is provided to support the findings and reproducibility of this research. (1) README: instructions on how to use the data and run the analysis code. (2) Folder1_Data: contains the raw data in CSV format. (3) Folder2_Scipts: contains the code for the analysis. (4) Folder3_Outputs: figures and plot generated for the study. [file 3833882.f1.zip › Data-analysis/Folder3_Outputs/Individual Q-Qplot/Q-Q8.png]

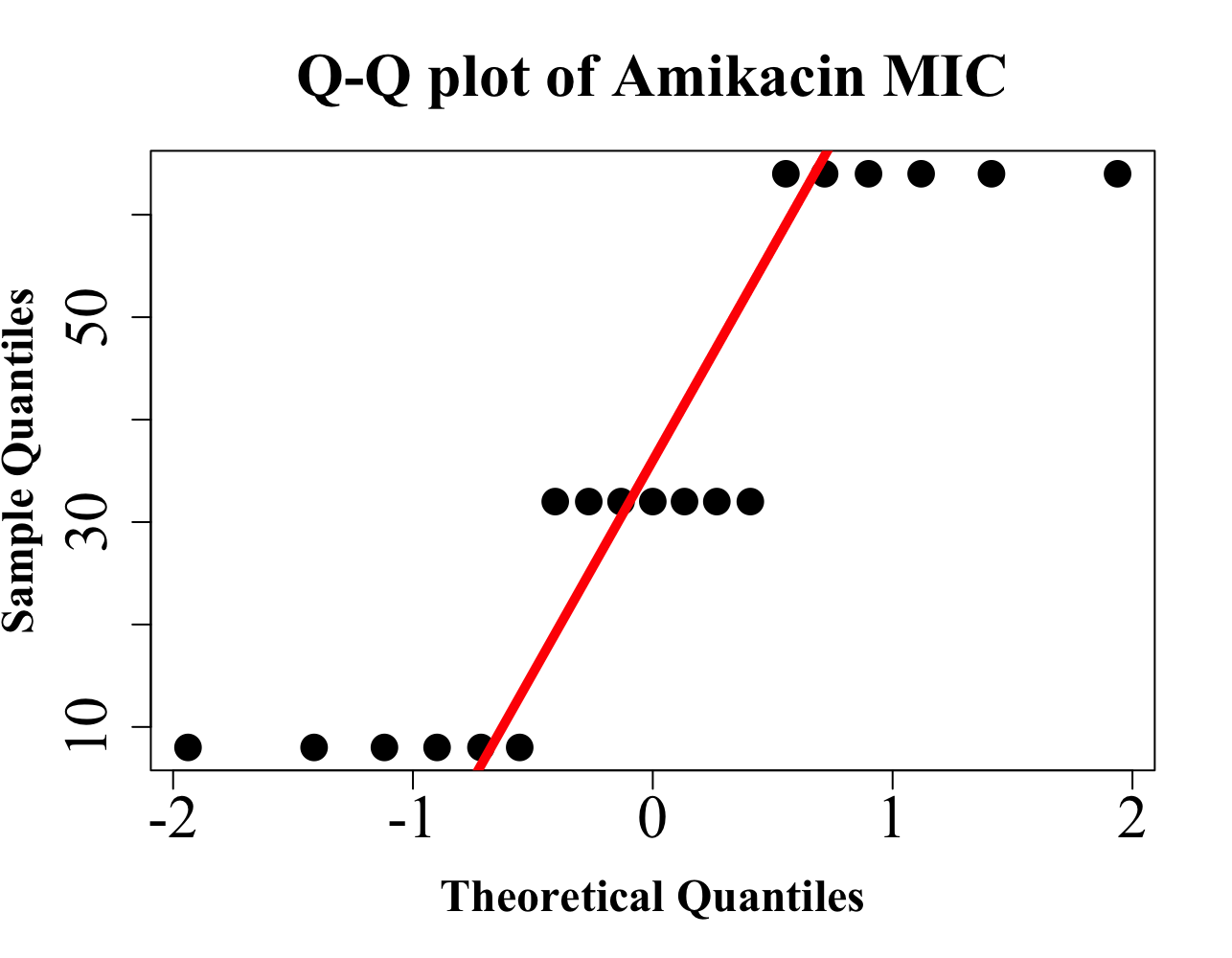

Supplement: Supporting Information — Additional supporting information can be found online in the Supporting Information section. The following supporting information is provided to support the findings and reproducibility of this research. (1) README: instructions on how to use the data and run the analysis code. (2) Folder1_Data: contains the raw data in CSV format. (3) Folder2_Scipts: contains the code for the analysis. (4) Folder3_Outputs: figures and plot generated for the study. [file 3833882.f1.zip › Data-analysis/Folder3_Outputs/Individual Q-Qplot/Q-Q7.png]

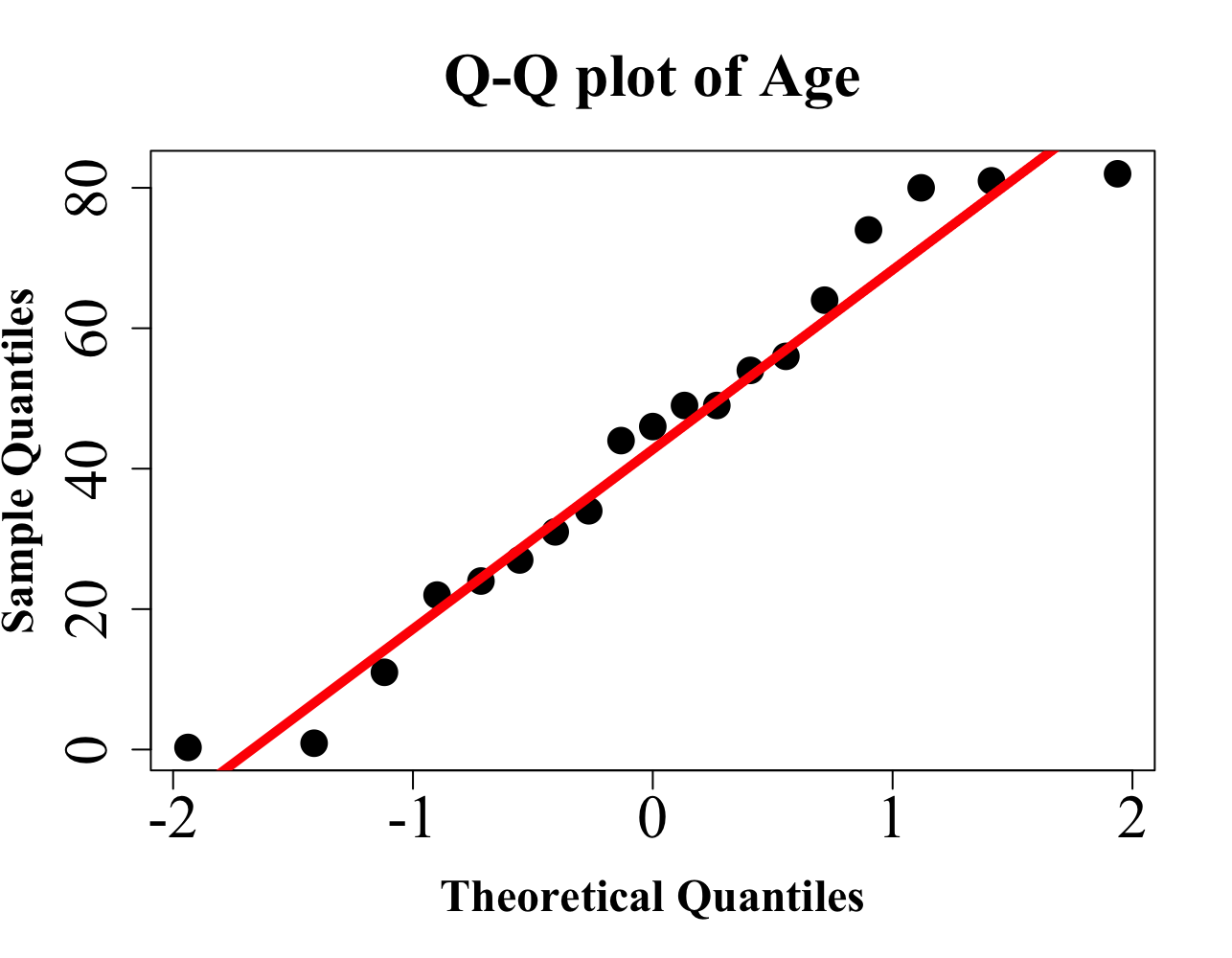

Supplement: Supporting Information — Additional supporting information can be found online in the Supporting Information section. The following supporting information is provided to support the findings and reproducibility of this research. (1) README: instructions on how to use the data and run the analysis code. (2) Folder1_Data: contains the raw data in CSV format. (3) Folder2_Scipts: contains the code for the analysis. (4) Folder3_Outputs: figures and plot generated for the study. [file 3833882.f1.zip › Data-analysis/Folder3_Outputs/Individual Q-Qplot/Q-Q1.png]

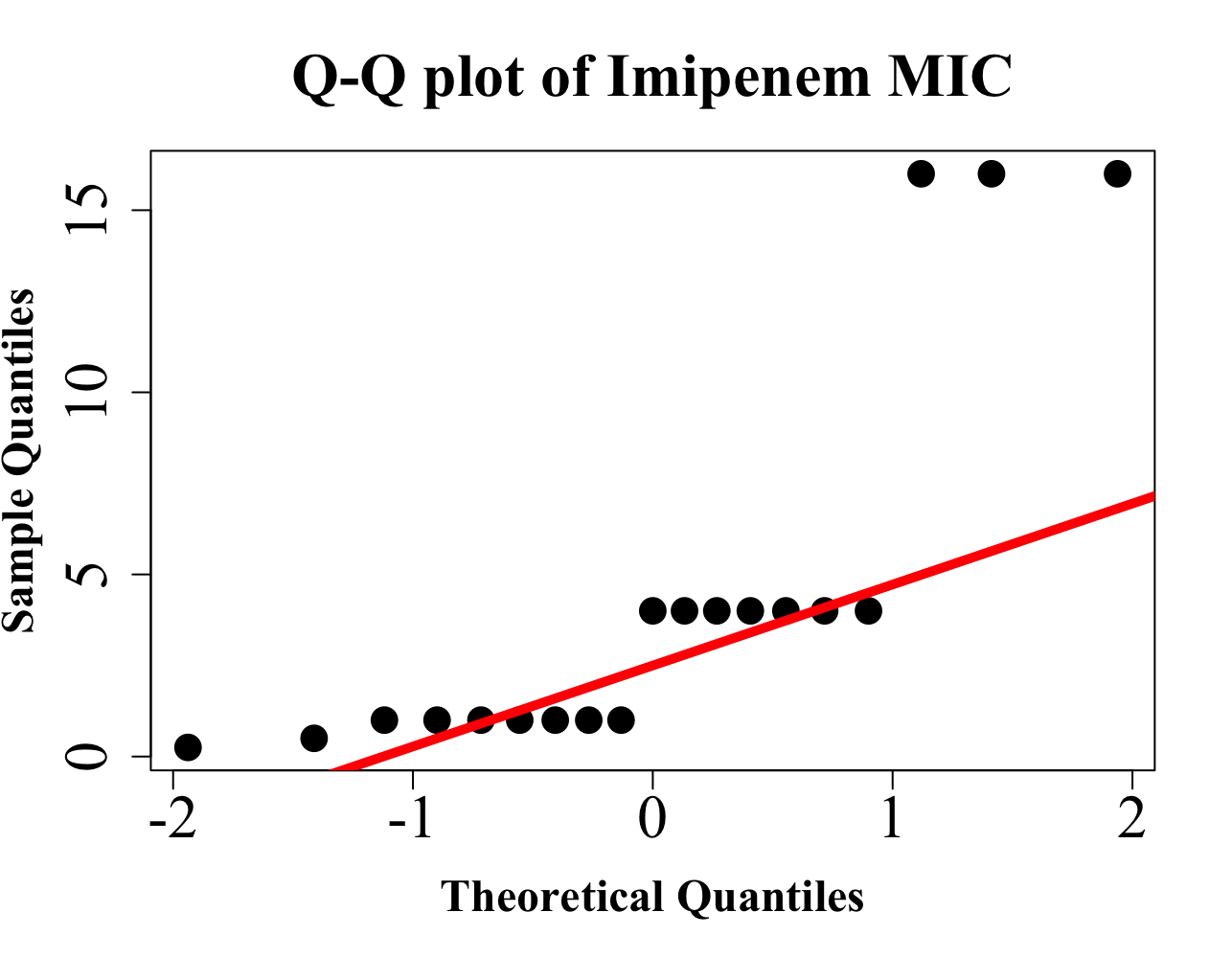

Supplement: Supporting Information — Additional supporting information can be found online in the Supporting Information section. The following supporting information is provided to support the findings and reproducibility of this research. (1) README: instructions on how to use the data and run the analysis code. (2) Folder1_Data: contains the raw data in CSV format. (3) Folder2_Scipts: contains the code for the analysis. (4) Folder3_Outputs: figures and plot generated for the study. [file 3833882.f1.zip › Data-analysis/Folder3_Outputs/Individual Q-Qplot/Q-Q6.png]

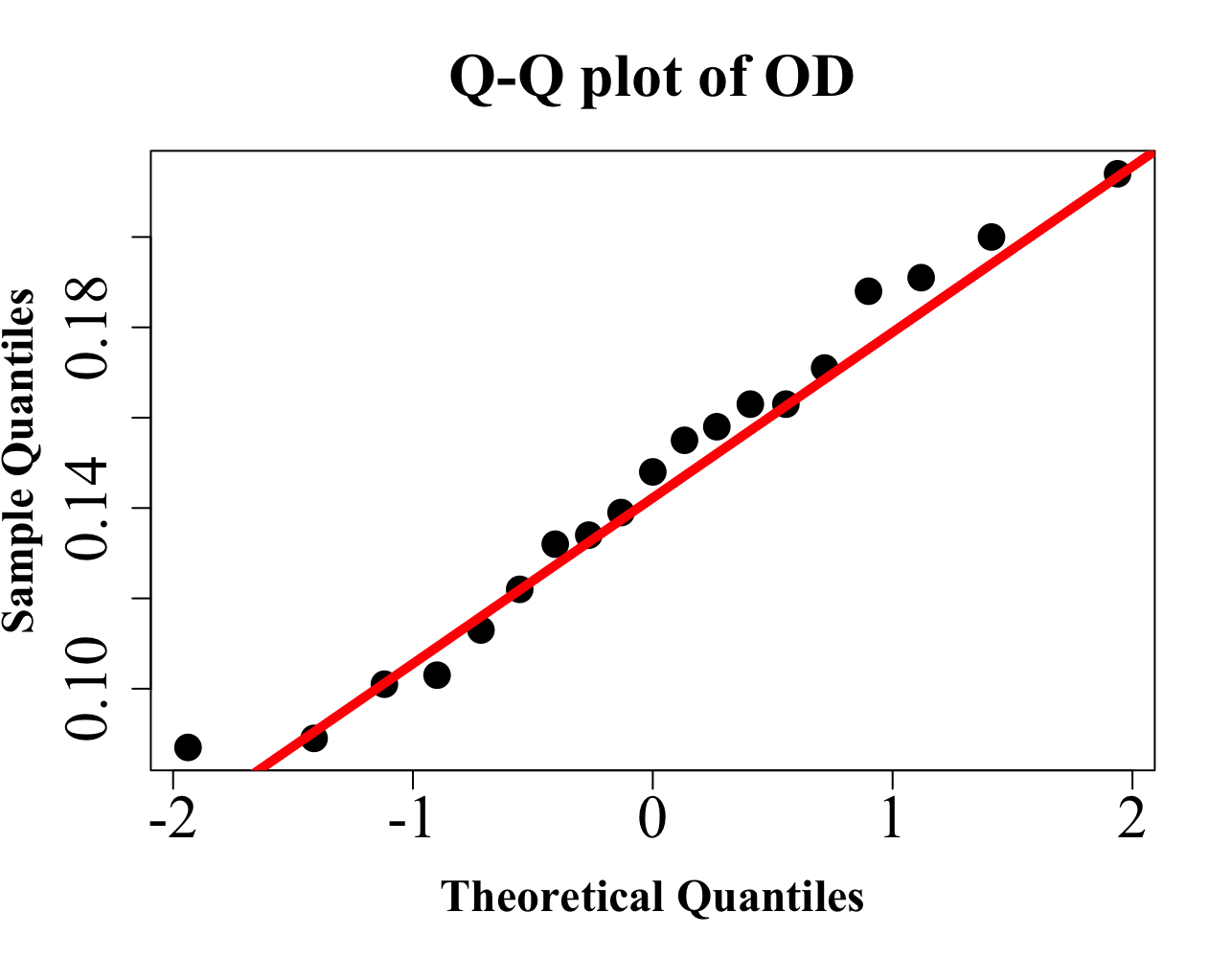

Supplement: Supporting Information — Additional supporting information can be found online in the Supporting Information section. The following supporting information is provided to support the findings and reproducibility of this research. (1) README: instructions on how to use the data and run the analysis code. (2) Folder1_Data: contains the raw data in CSV format. (3) Folder2_Scipts: contains the code for the analysis. (4) Folder3_Outputs: figures and plot generated for the study. [file 3833882.f1.zip › Data-analysis/Folder3_Outputs/Individual Q-Qplot/Q-Q2.png]

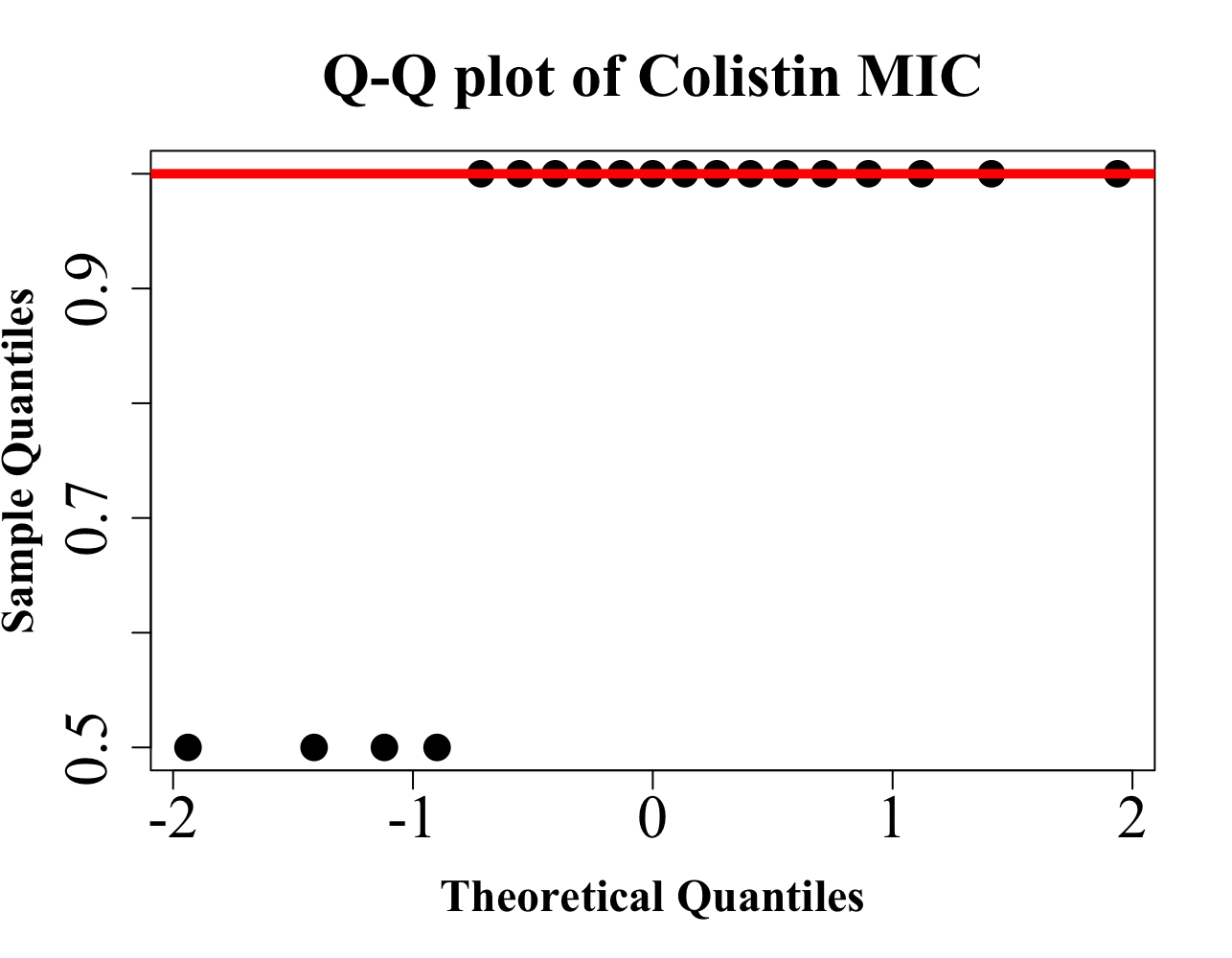

Supplement: Supporting Information — Additional supporting information can be found online in the Supporting Information section. The following supporting information is provided to support the findings and reproducibility of this research. (1) README: instructions on how to use the data and run the analysis code. (2) Folder1_Data: contains the raw data in CSV format. (3) Folder2_Scipts: contains the code for the analysis. (4) Folder3_Outputs: figures and plot generated for the study. [file 3833882.f1.zip › Data-analysis/Folder3_Outputs/Individual Q-Qplot/Q-Q11.png]

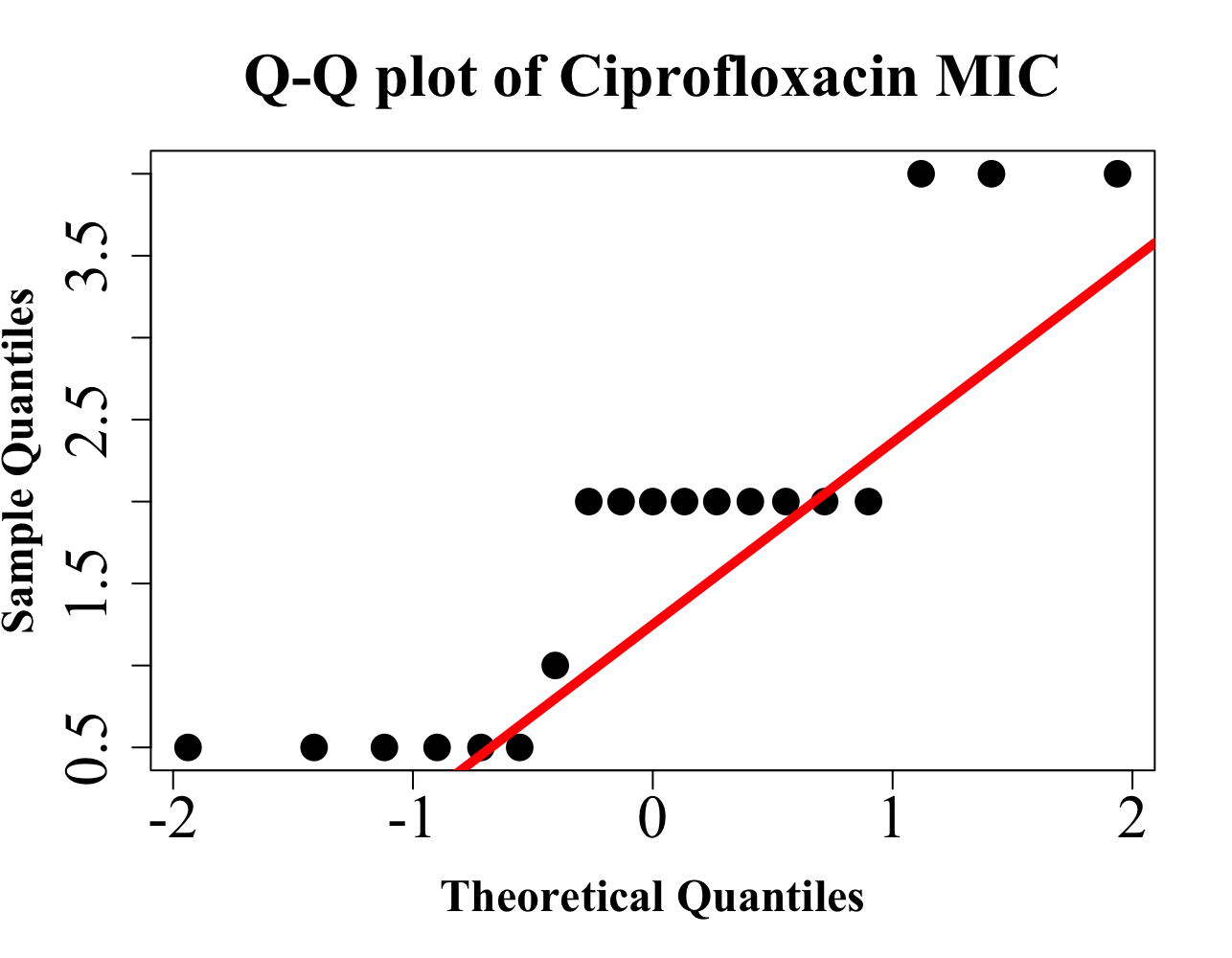

Supplement: Supporting Information — Additional supporting information can be found online in the Supporting Information section. The following supporting information is provided to support the findings and reproducibility of this research. (1) README: instructions on how to use the data and run the analysis code. (2) Folder1_Data: contains the raw data in CSV format. (3) Folder2_Scipts: contains the code for the analysis. (4) Folder3_Outputs: figures and plot generated for the study. [file 3833882.f1.zip › Data-analysis/Folder3_Outputs/Individual Q-Qplot/Q-Q9.png]

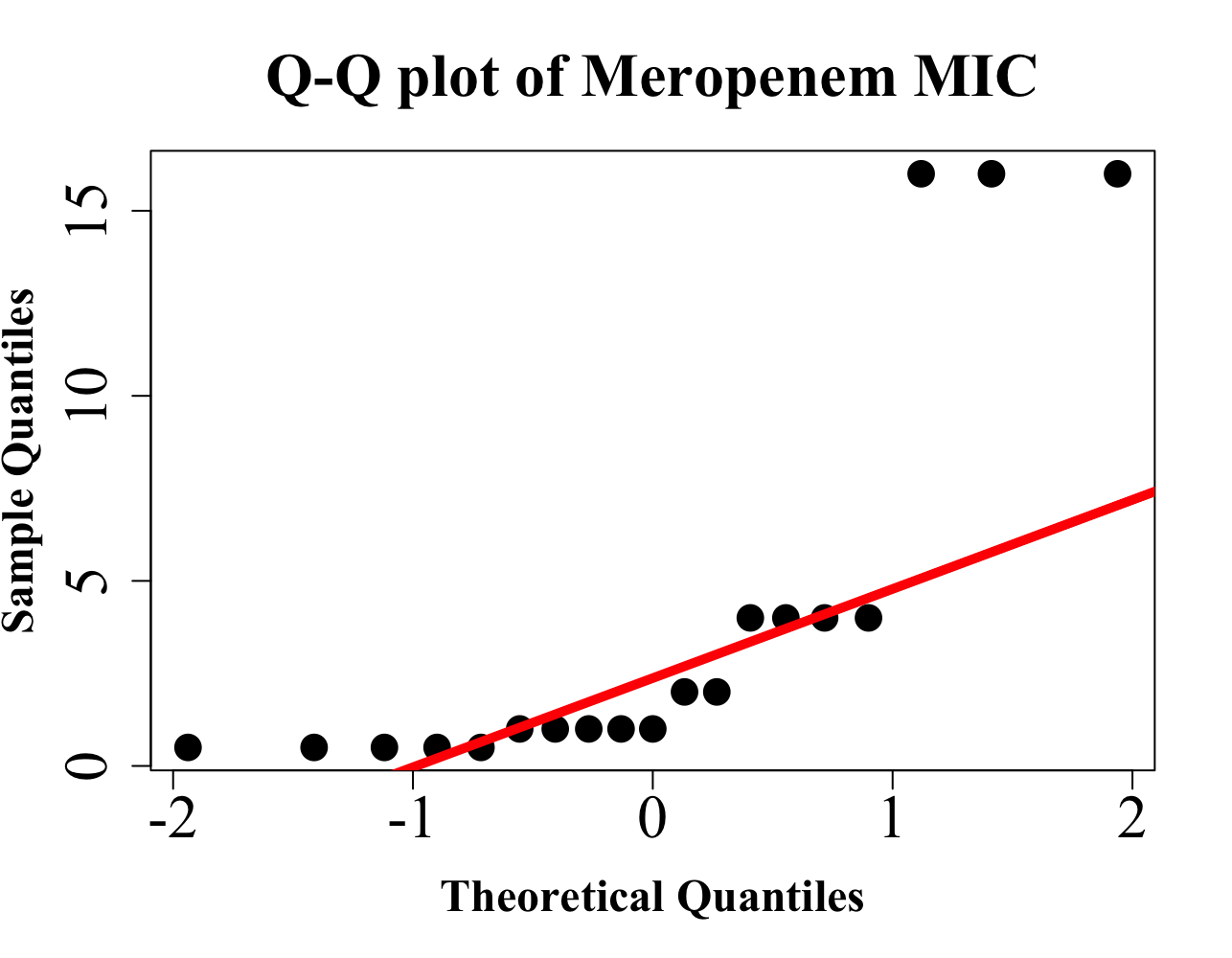

Supplement: Supporting Information — Additional supporting information can be found online in the Supporting Information section. The following supporting information is provided to support the findings and reproducibility of this research. (1) README: instructions on how to use the data and run the analysis code. (2) Folder1_Data: contains the raw data in CSV format. (3) Folder2_Scipts: contains the code for the analysis. (4) Folder3_Outputs: figures and plot generated for the study. [file 3833882.f1.zip › Data-analysis/Folder3_Outputs/Individual Q-Qplot/Q-Q5.png]

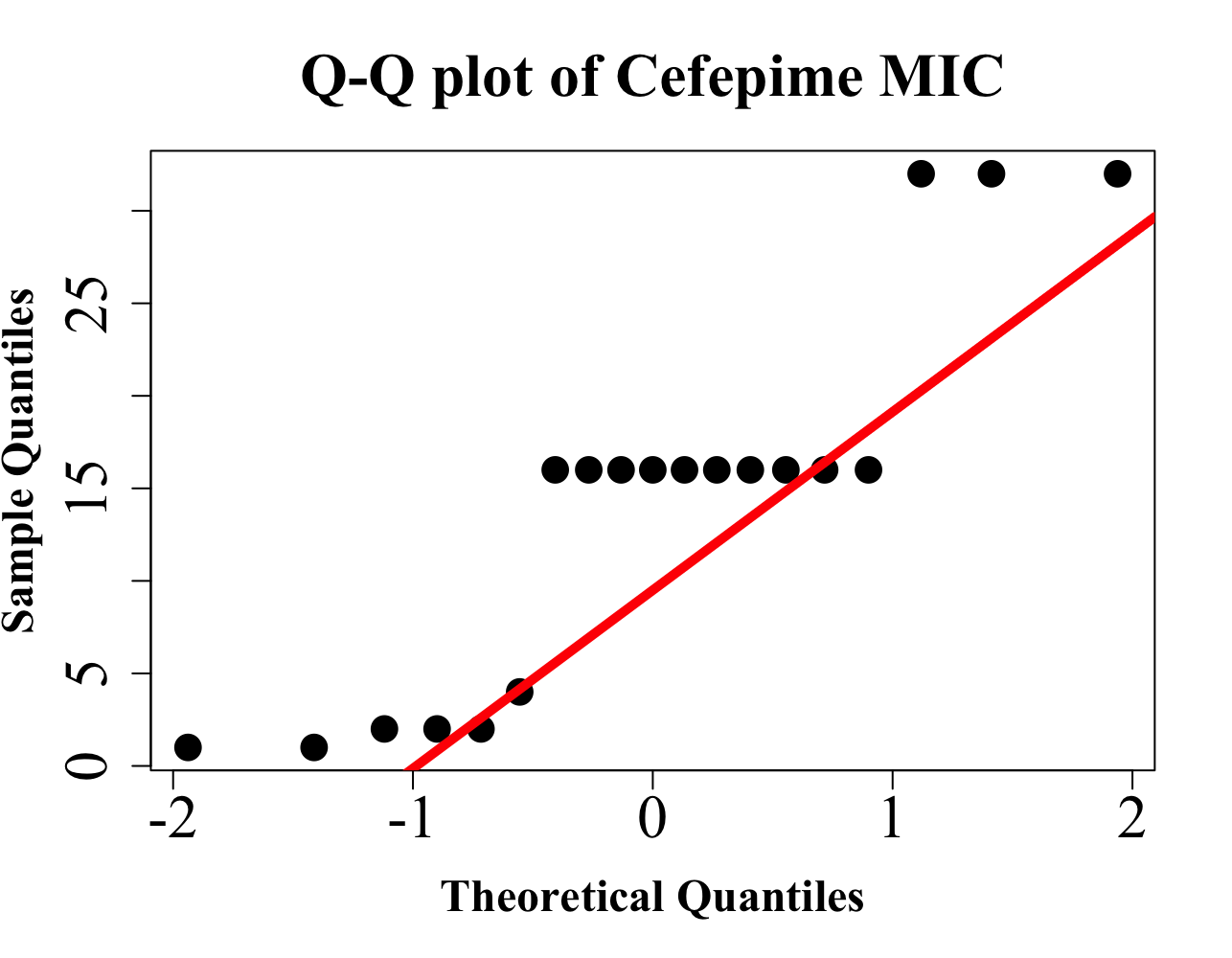

Supplement: Supporting Information — Additional supporting information can be found online in the Supporting Information section. The following supporting information is provided to support the findings and reproducibility of this research. (1) README: instructions on how to use the data and run the analysis code. (2) Folder1_Data: contains the raw data in CSV format. (3) Folder2_Scipts: contains the code for the analysis. (4) Folder3_Outputs: figures and plot generated for the study. [file 3833882.f1.zip › Data-analysis/Folder3_Outputs/Individual Q-Qplot/Q-Q3.png]

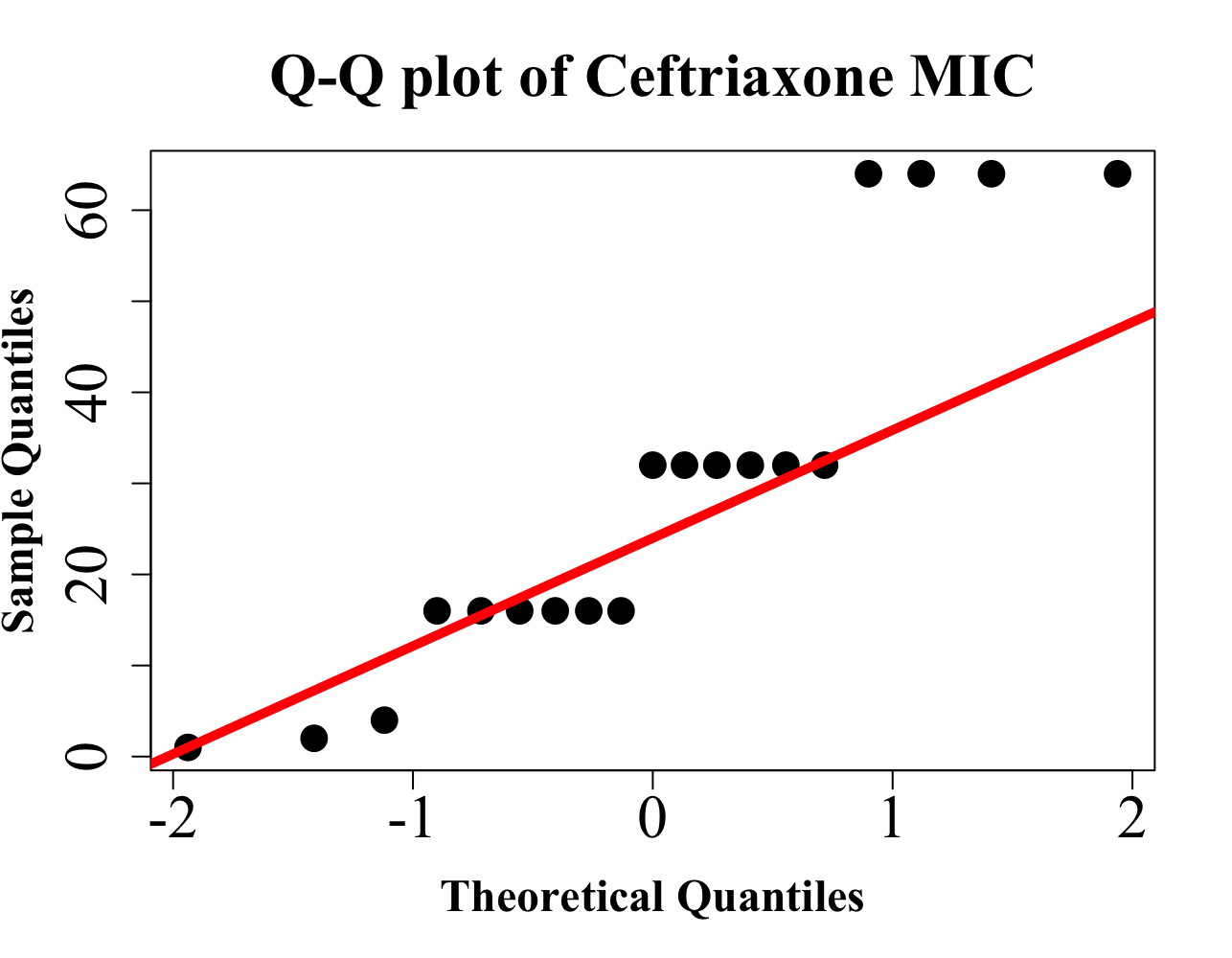

Supplement: Supporting Information — Additional supporting information can be found online in the Supporting Information section. The following supporting information is provided to support the findings and reproducibility of this research. (1) README: instructions on how to use the data and run the analysis code. (2) Folder1_Data: contains the raw data in CSV format. (3) Folder2_Scipts: contains the code for the analysis. (4) Folder3_Outputs: figures and plot generated for the study. [file 3833882.f1.zip › Data-analysis/Folder3_Outputs/Individual Q-Qplot/Q-Q4.png]

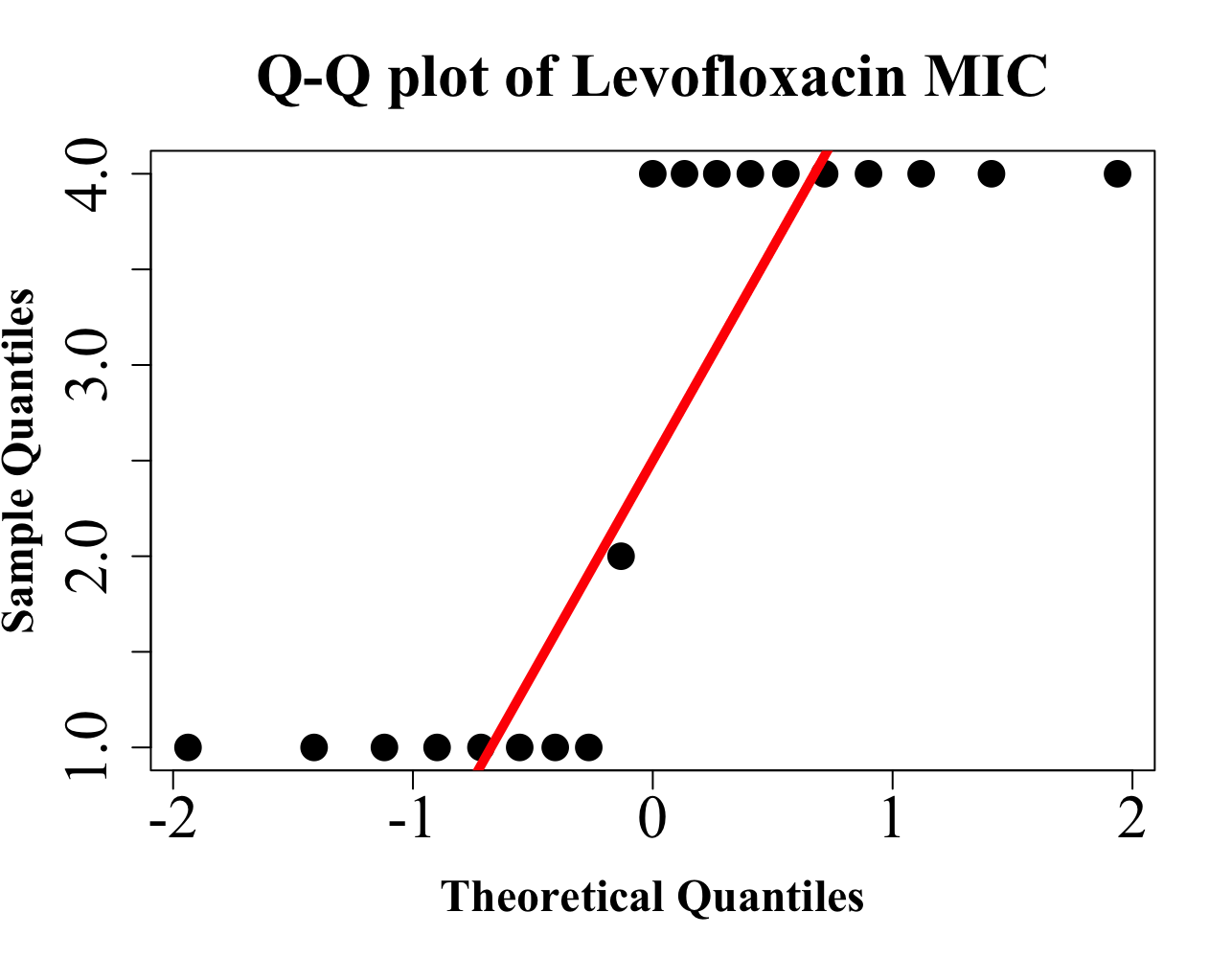

Supplement: Supporting Information — Additional supporting information can be found online in the Supporting Information section. The following supporting information is provided to support the findings and reproducibility of this research. (1) README: instructions on how to use the data and run the analysis code. (2) Folder1_Data: contains the raw data in CSV format. (3) Folder2_Scipts: contains the code for the analysis. (4) Folder3_Outputs: figures and plot generated for the study. [file 3833882.f1.zip › Data-analysis/Folder3_Outputs/Individual Q-Qplot/Q-Q10.png]

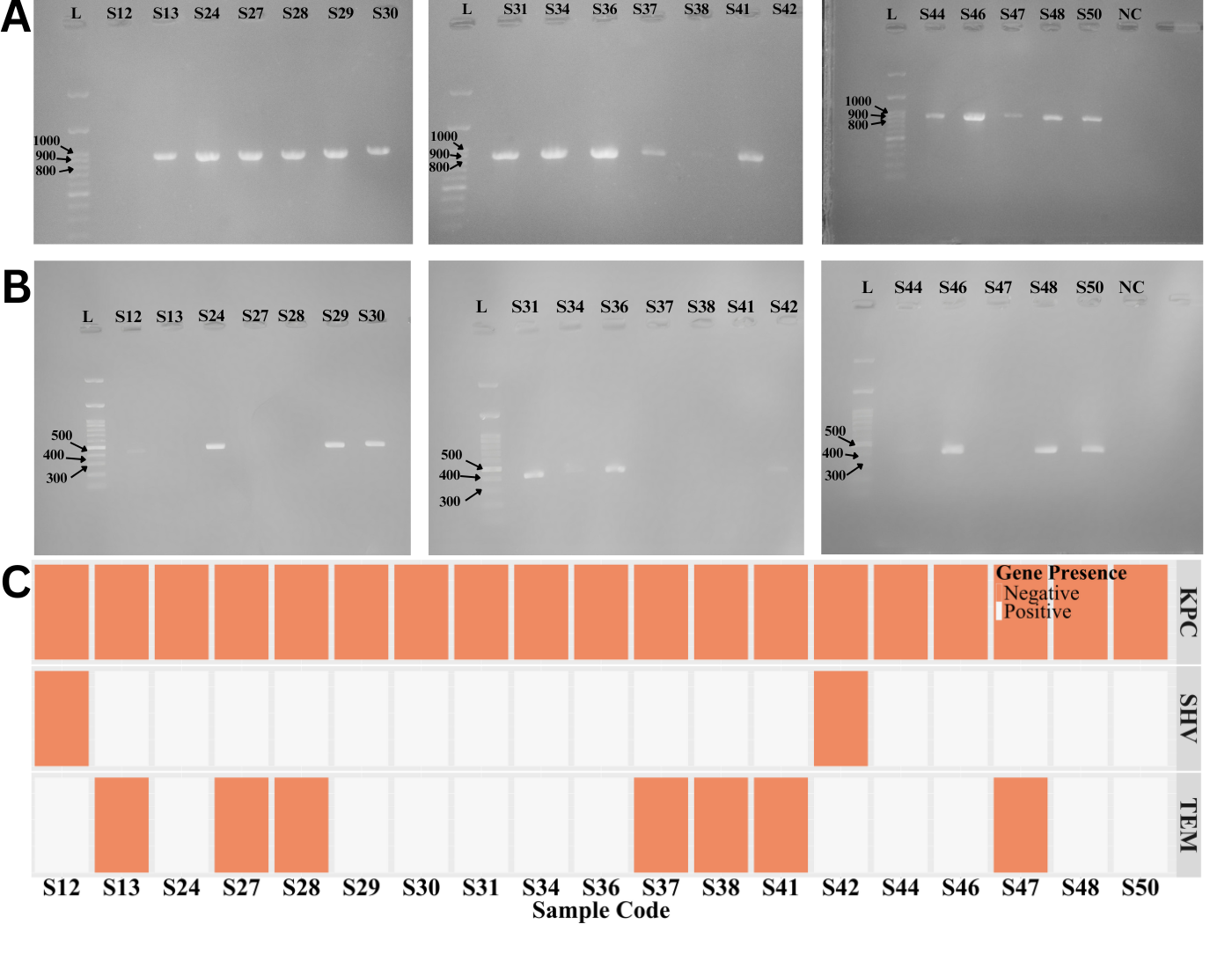

Supplement: Supporting Information — Additional supporting information can be found online in the Supporting Information section. The following supporting information is provided to support the findings and reproducibility of this research. (1) README: instructions on how to use the data and run the analysis code. (2) Folder1_Data: contains the raw data in CSV format. (3) Folder2_Scipts: contains the code for the analysis. (4) Folder3_Outputs: figures and plot generated for the study. [file 3833882.f1.zip › Data-analysis/Folder3_Outputs/ARG_gel_images.png]
